# Supplementary material for: Nonmyeloablative pentostatin-cyclophosphamide preconditioning improves rates of engraftment in adults undergoing haploidentical HCT for sickle cell disease
Source: PLoS One. 2026 Mar 23;21(3):e0332282. doi: 10.1371/journal.pone.0332282 (PMC13008046; doi:10.1371/journal.pone.0332282)
Supplement: S1 Table — Estimated GFR (eGFR) was calculated before each dose of pentostatin, with the dose adjusted as noted above. The GFR was calculated based on cystatin-C using the Chronic Kidney Disease Epidemiology Collaboration (CKD-EPI) GFR calculation; in the absence of cystatin-C data, the Cockcroft-Gault formula was used. (PDF) [file pone.0332282.s001.pdf]

| Pentostatin Dose Administration Based on eGFR |
| --- |
| 4mg/$m^2$ if eGFR >60 |
| 3mg/$m^2$ if eGFR 40-60 |
| 2mg/$m^2$ if eGFR 20-39 |
| 1mg/$m^2$ if eGFR <20 mL/min/1.73$m^2$. |

S1 Table: eGFR-based Pentostatin Dosing. Estimated GFR (eGFR) was calculated before each dose of pentostatin, with the dose adjusted as noted above. The GFR was calculated based on cystatin-C using the Chronic Kidney Disease Epidemiology Collaboration (CKD-EPI) GFR calculation; in the absence of cystatin-C data, the Cockcroft-Gault formula was used.
