# Supplementary material for: Nonmyeloablative pentostatin-cyclophosphamide preconditioning improves rates of engraftment in adults undergoing haploidentical HCT for sickle cell disease
Source: PLoS One. 2026 Mar 23;21(3):e0332282. doi: 10.1371/journal.pone.0332282 (PMC13008046; doi:10.1371/journal.pone.0332282)
Supplement: S2 Table — 1 Pentostatin will not be dose-adjusted based on ALC/ANC values. 2 For ANC values <1000, decrease in cyclophosphamide dosing only – patients will not receive G-CSF. 3 Cyclophosphamide dose indicated will be continued daily until the next ALC/ANC measurements. (PDF) [file pone.0332282.s002.pdf]

| Cyclophosphamide dose adjustment based on absolute lymphocyte count (ALC) and absolute neutrophil count (ANC) values | | | | | | |
|---|---|---|---|---|---|---|
| Day of cycle[1] | | ALC value at time of evaluation | | ANC value at time of evaluation[2] | | Cyclophosphamide dose[3] |
| -21 | | Any | | > 1000 | | 200 |
| | | | | | | |
| -17 | | ≥ 400 | | > 1000 | | 200 |
| | | 200-399 | | 500-999 | | 100 |
| | | < 200 | | < 500 | | 0 |
| | | | | | | |
| -13 | | ≥ 200 | | > 1000 | | 200 |
| | | 100-199 | | 500-999 | | 100 |
| | | < 100 | | < 500 | | 0 |
| | | | | | | |
| -9 | | ≥ 100 | | > 1000 | | 200 |
| | | 50-99 | | 500-999 | | 100 |
| | | < 50 | | < 500 | | 0 |

S2 Table: Protocol for cyclophosphamide dose adjustment

[1] *Pentostatin will not be dose-adjusted based on ALC/ANC values.*

[2] *For ANC values <1000, decrease in cyclophosphamide dosing only – patients will not receive G-CSF.*

[3] *Cyclophosphamide dose indicated will be continued daily until the next ALC/ANC measurements.*
