# Supplementary material for: Nonmyeloablative pentostatin-cyclophosphamide preconditioning improves rates of engraftment in adults undergoing haploidentical HCT for sickle cell disease
Source: PLoS One. 2026 Mar 23;21(3):e0332282. doi: 10.1371/journal.pone.0332282 (PMC13008046; doi:10.1371/journal.pone.0332282)
Supplement: S3 Table — HCT: hematopoietic cell transplant, IVIG: intravenous immunoglobulin. (PDF) [file pone.0332282.s003.pdf]

Immunohematologic complications	Time post-HCT at onset	Chimerism at onset	Therapies attempted	Outcome
**Haplo-1 Protocol— no immunohematologic complications noted**				
**Haplo-PC Protocol**				
Evans Syndrome	6.4 months	Chimerism: Myeloid 100%/CD3 76%	• steroids, rituximab<br>• eltrombopag, avatrombopag<br>• daratumumab<br>• fostamatinib<br>• eculizumab<br>• abatacept<br>• high-dose cyclophosphamide<br>• splenectomy	death
Evans Syndrome	9.3 months	95% myeloid/ 67% CD3	• steroids & IVIG<br>• fostamatinib<br>• rituximab x 4; prednisone<br>• daratumumab<br>• erythropoietin, eculizumab<br>• high-dose cyclophosphamide<br>• splenectomy	death
Autoimmune Hemolytic anemia	2.4 years	80% myeloid/ 98% CD3	IVIG, steroids, rituximab	resolved
Autoimmune Hemolytic anemia	2.6 years	100% myeloid/ 99% CD3	IVIG, steroids, rituximab	resolved

S3 Table: Summary of immune-mediated hematologic complications after HCT on Haplo-1 and Haplo-PC protocols. HCT: hematopoietic cell transplant, IVIG: intravenous immunoglobulin.
