# Supplementary material for: Nonmyeloablative pentostatin-cyclophosphamide preconditioning improves rates of engraftment in adults undergoing haploidentical HCT for sickle cell disease
Source: PLoS One. 2026 Mar 23;21(3):e0332282. doi: 10.1371/journal.pone.0332282 (PMC13008046; doi:10.1371/journal.pone.0332282)
Supplement: S1 Fig — a)Cumulative incidence of graft failure at last follow-up in patients receiving 50 mg/kg PT-Cy on haplo-1 and all haplo-PC patients (who also received 50 mg/kg PT-Cy) b) Cumulative incidence of graft failure at last follow-up in patients receiving 100 mg/kg PT-Cy on haplo-1 and all haplo-PC patients. (PDF) [file pone.0332282.s004.pdf]

a)

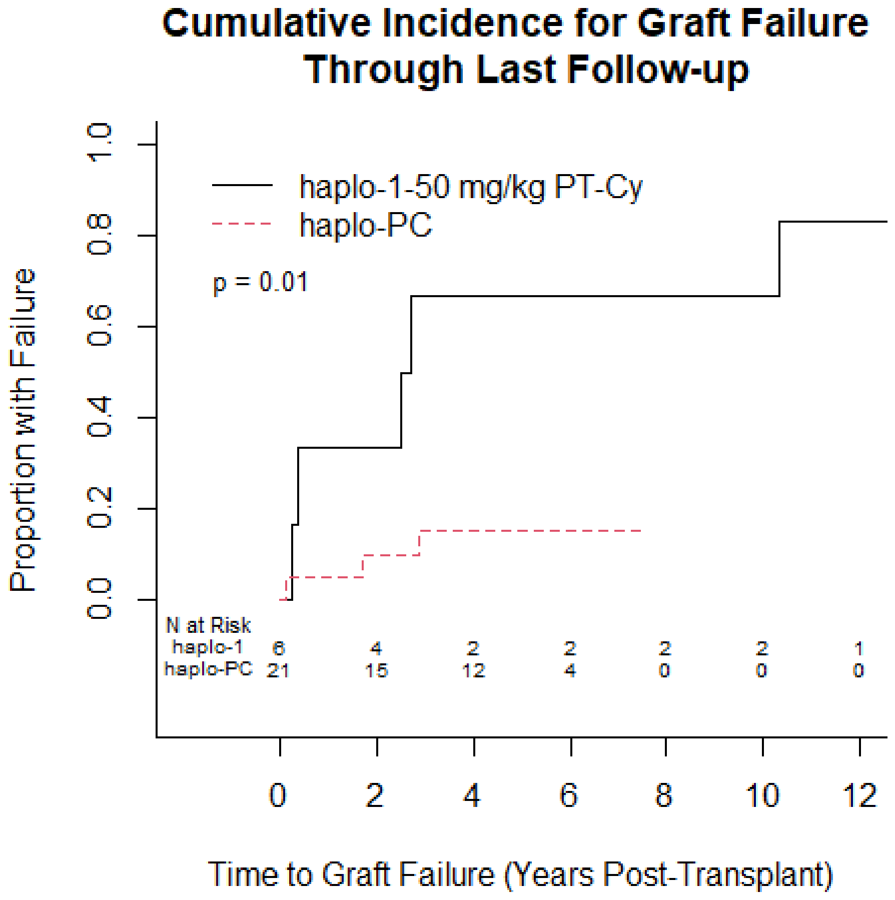
Cumulative Incidence for Graft Failure
Through Last Follow-up
haplo-1-50 mg/kg PT-Cy
haplo-PC
p = 0.01
Proportion with Failure
0.0
0.2
0.4
0.6
0.8
1.0
N at Risk
haplo-1 6 4 2 2 2 2 1
haplo-PC 21 15 12 4 0 0 0
0
2
4
6
8
10
12
Time to Graft Failure (Years Post-Transplant)

b)

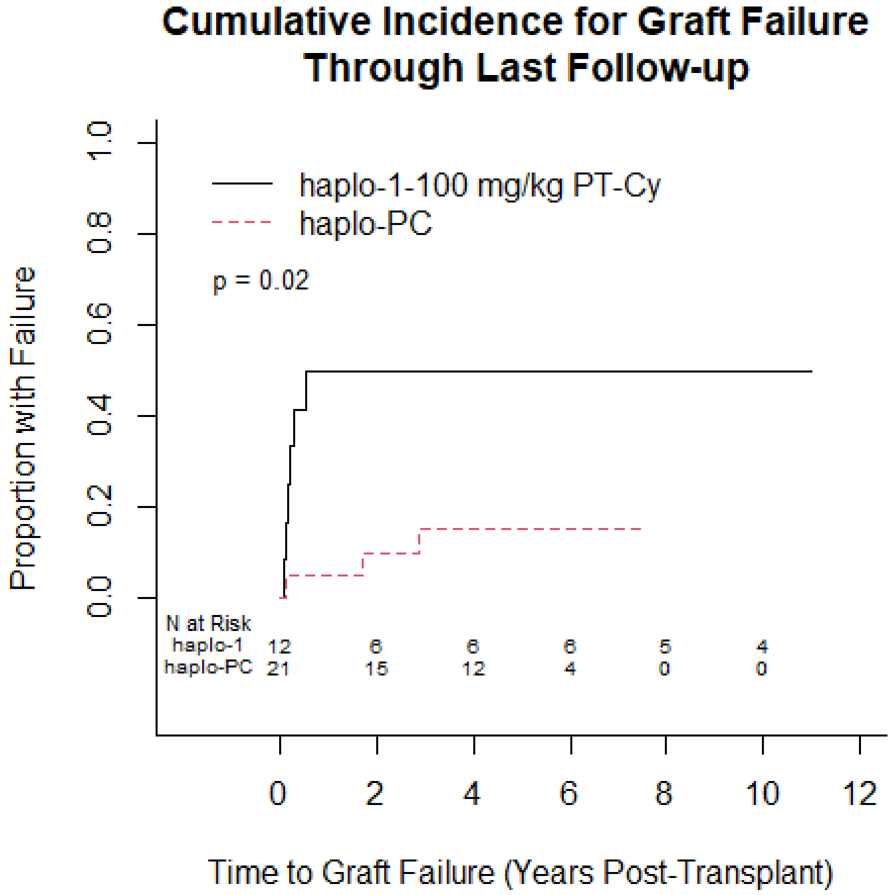

S1 Figure: Graft failure at last follow-up a)Cumulative incidence of graft failure at last follow-up in patients receiving 50 mg/kg PT-Cy on haplo-1 and all haplo-PC patients (who also received 50 mg/kg PT-Cy) b) Cumulative incidence of graft failure at last follow-up in patients receiving 100 mg/kg PT-Cy on haplo-1 and all haplo-PC patients
