# Supplementary material for: Nonmyeloablative pentostatin-cyclophosphamide preconditioning improves rates of engraftment in adults undergoing haploidentical HCT for sickle cell disease
Source: PLoS One. 2026 Mar 23;21(3):e0332282. doi: 10.1371/journal.pone.0332282 (PMC13008046; doi:10.1371/journal.pone.0332282)
Supplement: S3 Fig — (PDF) [file pone.0332282.s006.pdf]

CLINICAL RESEARCH PROTOCOL

Project: # 17-H-0069
Drugs: Sirolimus /Alemtuzumab/Cyclophosphamide /Pentostatin /Hydroxyurea
IND: Exempt

Date: 15 JUN 2024

Title: Nonmyeloablative Haploidentical Peripheral Blood Mobilized Hematopoietic Precursor Cell Transplantation for Sickle Cell Disease

Other underlying words: Peripheral blood stem cells, host-donor chimerism, graft-versus-host disease, graft- versus-marrow, Sirolimus (Rapamune®), low-dose irradiation, alemtuzumab (Campath®), cyclophosphamide (Cytoxan®), donor apheresis, pentostatin (Nipent®)

Principal Investigator: Courtney D. Fitzhugh MD, CMTB, NHLBI (E)

Subjects of study:	Number	Sex	Age range
Patients	44 - 49	either	≥ 18 years
Donors	44 - 49	either	2-80 years

Accrual Ceiling: 98
Target enrollment up to 98 evaluable subjects to allow for: screen fails, transplant failures, withdrawals, or PI's discretion to terminate the subject from the study at any time (regardless of subject's consent).

**Project involves ionizing radiation?**	Yes
**Off-site project?**	No
**Multi-Institutional project?**	No
**DSMB**	Yes

Investigational Agents and/or Devices:

Device:	MRI: Image reconstruction software - Philips<br>MRI: Image analysis software - VUMC<br>MRI: Research MRI pulse sequences - VUMC
IND Number:	NSR IDE
Sponsor:	NHLBI OCD

PRECIS

Nonmyeloablative allogeneic peripheral blood stem cell (PBSC) transplants are currently being investigated in phase I/II trials assessing engraftment, efficacy, and toxicity at a number of transplant centers. Our ongoing protocol for patients with severe congenital anemias, particularly sickle cell disease (SCD), and an HLA-matched sibling donor has had excellent preliminary results. None of the patients who engrafted had sickle-related events or any evidence of graft versus host disease (GVHD). There was no significant toxicity associated with the conditioning regimen. An additional protocol is ongoing for patients with high risk of graft rejection which employs pentostatin and oral cyclophosphamide (PC) pre-transplant to further deplete recipient lymphocytes in an attempt to decrease the rate of graft rejection. Four of 4 patients transplanted remain free of SCD.

Our main limitation has been a lack of HLA-matched sibling donors in the majority of patients. We performed a study in which patients with severe SCD who lacked a suitable donor underwent a search for a matched unrelated donor or umbilical cord donor. The vast majority of patients were not found to have an appropriate alternative donor. We therefore seek to develop a safe nonmyeloablative regimen to be applied to the haploidentical setting so that family members can serve as donors and greatly expand the donor pool.

We developed a nonmyeloablative haploidentical PBSC transplant protocol which included 3 cohorts, with stopping rules built in for regimen failure, defined as graft rejection or severe GVHD. All included 400 cGy total body irradiation (TBI) in divided doses 1 and 2 days prior to transplant, alemtuzumab, and sirolimus. The first cohort included no cyclophosphamide. The 2nd included one dose of cyclophosphamide given at 50mg/kg on day 3 post-transplant, and the 3rd included 100mg/kg cyclophosphamide given in divided doses on days 3 and 4 post-transplant. The engraftment rate and percentage of patients who remained free of SCD improved with each successive cohort. However, the graft rejection rate in the 3rd cohort remained high at 50%. To attempt to reduce the rate of graft rejection in the haploidentical setting, this protocol will add PC to the conditioning regimen.

In this protocol, we propose PBSC transplantation in patients with SCD considered at high risk for complications from or ineligible for standard bone marrow transplantation, with allogeneic peripheral blood stem cells from a haploidentical donor using a novel immunosuppressive regimen without myeloablation in an attempt to further decrease the transplant-related morbidity/mortality. The low intensity nonmyeloablative conditioning regimen will consist of a relatively low radiation dose for therapeutic radiation, Alemtuzumab (Campath®), Sirolimus (Rapamune®), Cyclophosphamide (Cytoxan®), and pentostatin (Nipent®) as a strategy to provide adequate immunosuppression to allow sufficient engraftment for clinical remission with a lower risk of GVHD development. T-cell replete, donor-derived, granulocyte colony-stimulating factor (G-CSF)-mobilized PBSC will be used to establish hematopoietic and lymphoid reconstitution.

The primary endpoint of this study is the percentage of patients at 100 days post-transplant who have not rejected their grafts, and who are without severe GVHD (defined as grade 3 and higher acute GVHD and moderate to severe chronic GVHD). Other endpoints include degree of donor-host chimerism necessary for long-term graft survival and disease amelioration, incidence of acute and chronic GVHD, incidence of graft rejection, transplant-related morbidity, as well as disease-free and overall survival.

Contents

OBJECTIVES 7
BACKGROUND..7
SCIENTIFIC AND CLINICAL JUSTIFICATION 10
STUDY DESIGN..17
PATIENT SELECTION 18
Inclusion criteria- recipients (must fulfill one disease category in 5.1.1 and allof 5.1.2)..18
Exclusion criteria –recipient (any of the following would exclude the subject from participating) 18
Inclusion criteria - donor 19
Exclusion criteria- donor 19
TREATMENT PLAN 19
HLA typing 19
Exchange Transfusion (See Appendix C) 19
Collection of Autologous Peripheral Blood Stem Cells (PBSC, see Appendix D) 20
Preparative regimen..20
Central venous line placement 23
Supportive Care..23
Peripheral blood progenitor cell transplant (see Appendix C)..24
GVHD prophylaxis 24
Nutrition 24
Contraindication Listings 24
6.11 Bone Marrow Harvest 24
STUDY PARAMETERS 25
Primary endpoint 25
Secondary endpoints..25
RESEARCH STUDIES..26
Chimerism studies 26
Bone Marrow Samples 26
Hematopoietic Stem Cells 26
Transthoracic Echocardiography..27
Cardiac MRI..27
Dual X-ray Absorptiometry (DEXA Scan) 27
Right heart catheterization..27
Assessment of Quality of Life 27
Neuropsychologic testing 27
Immune reconstitution..28
Assessment of cytokines and lymphocyte function..28
Evaluation for markers of graft rejection 28
Pharmacokinetic studies 29
Pharmacogenomic studies 29
HLA antibodies and C1q testing……………………………………………………………29
Functional Fluidics collaboration to study red blood cell biology……………………………………..29
Evaluation of the effect of curative therapy on inflammatory and coagulation markers………………30
Brain MRI/MRA with CBF and OEF…………………………………………………………30

HUMAN SPECIMEN USE, DISPOSITION, TRACKING, STORAGE OF SAMPLES & DATA........................31
CLINICAL EVALUATION AND PLAN – SCD PATIENT 33
Screening evaluation 33
Baseline evaluations………………………………………………………………………….....32
Inpatient monitoring...35
Follow-up to day 100: outpatient 36
Beyond day 100...36
CLINICAL EVALUATION AND PLAN – DONOR 37
Pre-study consult and evaluation...37
MANAGEMENT OF SCD PATIENT COMPLICATIONS 37
Viral reactivation...38
Acute GVHD...38
Chronic GVHD 38
Graft rejection 38
Relapse of disease 38
Use of Immunosuppressive drugs 38
DATA AND SAFETY MONITORING...38
Safety Monitoring..38
Assessment of Safety 38
Documentation of data capture..38
Grading of adverse events 39
Attribution of Adverse Events..39
NIH Intramural IRB and CD reporting 39
HUMAN SUBJECT PROTECTIONS 40
Rationale for subject selection 40
Participation of children 40
Exclusion – Cognitively Impaired……………………………………………………….......40
Exclusion – Pregnant and Nursing Women…………………………………………………41
Hazards and discomforts- recipient 41
Non-significant Risk (NSR) Device Determination…………………………………………..49
Risks in relation to benefit…………………………………………………………………….49
Informed consent...48
COMPENSATION………………………………………………………………………….…51
STATISTICAL CONSIDERATIONS 51
ON STUDY DATE: Date of consent signing 55
OFF STUDY CRITERIA 55
PHARMACEUTICALS..55
Off-Label Use of Drugs..56
Alemtuzumab 56
Cyclophosphamide..56
Pentostatin 56
Filgrastim 57
6 Hydroxyurea..57
18.7 Sirolimus 57
Center for International Blood and Marrow Transplant Research (CIBMTR)...54

REFERENCES..58

APPENDIX A: ECOG PERFORMANCE STATUS SCALE
APPENDIX B: TRANSFUSION OF RED CELLS TO RECIPIENTS OF ABO INCOMPATIBLE MARROW
APPENDIX C: TRANSFUSION MEDICINE GUIDELINES FOR APHERESIS PROCEDURES IN SICKLE CELL PATIENTS RECEIVING NONMYELOABLATIVE ALLOGENEIC PBSC TRANSPLANTS
APPENDIX D: COLLECTION OF AUTOLOGOUS PERIPHERAL BLOOD STEM CELLS
APPENDIX E: SAMPLE CALENDAR
APPENDIX F: REPRODUCTIVE QUESTIONNAIRES IN PATIENTS WITH SEVERE CONGENITAL ANEMIAS BEFORE AND AFTER TRANSPLANT
APPENDIX G: SCHEDULE OF EVENTS
APPENDIX H: POWER ANALYSIS OF ABILITY TO DETECT A DIFFERENCE IN PRIMARY OUTCOME BY RADIATION REGIMEN
APPENDIX I : REPRODUCTIVE / FERTILITY QUESTIONNAIRES

SEE ALSO: TRANSPLANT AND CELLULAR THERAPY (TCT) CONSORTIUM GUIDELINES
Marrow stem cell transplant section
CMTB, NHLBI
http://intranet.cc.nih.gov/bmt/

OBJECTIVES

Using a nonmyeloablative preparative regimen followed by an allogeneic haploidentical granulocyte colony- stimulating factor (filgrastim, G-CSF)-mobilized peripheral blood stem cell transplant (PBSCT) in a population of patients with sickle cell disease (SCD) at increased risk for complications, with or ineligible for standard myeloablative allo-transplantation, we hope to assess the following primary objectives:

Determine if the regimen exhibits 70% or better success without acute grade III or higher GVHD or moderate to severe chronic GVHD.

Evaluate the 1 year engraftment success probability in SCD patients who have not been transfused in the previous 3 months.

Examine the level of chimerism required to maintain both graft survival as well as hematologic normalcy using a novel nonmyeloablative conditioning regimen.

The following secondary objectives will also be evaluated:

Evaluate the safety, efficacy, and toxicity, including disease-free survival, overall survival, and transplant-related mortality, of this novel nonmyeloablative conditioning regimen.

Evaluate the incidence of viral reactivation and disease.

Evaluate biomarkers such as cytokines and immunophenotypic cells associated with early graft rejection and tolerance induction.

Evaluate the incidence and severity of regimen failure when various haploidentical donors (i.e. mother versus father, parent versus sibling, and parent versus child) are employed.

Evaluate whether cyclophosphamide pharmacokinetics and/or germline allelic variants in drug metabolizing/transporting genes differ in patients with and without renal insufficiency and in patients who do and do not experience graft rejection.

Evaluate whether pentostatin pharmacokinetics differ in patients who do and do not experience graft rejection.

BACKGROUND

Introduction

Allogeneic bone marrow (and peripheral blood) transplantation (BMT) is the only available cure for patients with sickle cell disease (SCD), but has been infrequently pursued due to its associated complications. The majority of patients who are otherwise eligible for BMT do not have a suitable donor. In addition, the unacceptable risk of death from conventional BMT renders many patients, especially those with nonmalignant disorders, ineligible for what may otherwise be curative therapy. Recently however, in both malignant and non-malignant disorders, it has been shown that these high intensity regimens are not necessary for engraftment and survival, and many centers are currently exploring nonmyeloablative conditioning regimens in order to reduce the toxicity associated with this treatment modality. While successful engraftment has been reported in the majority of patients conditioned with reduced intensity regimens, these regimens still carry significant toxicity and have not significantly reduced the risk of graft-versus-host disease (GVHD). For patients with SCD, the replacement of abnormal erythroid cells with normal donor- derived erythroid cells is required for disease amelioration. SCD constitutes an ideal situation for a nonmyeloablative conditioning regimen as only a proportion of normal cells will need to engraft given the survival and proliferative advantage of the donor-derived erythroid cells as compared to the host cells. Further, as a nonmyeloablative regimen should allow autologous recovery with a low risk of adverse consequences to the recipient if the graft should fail, graft failure is preferable to the development of severe GVHD. As such, we propose the development of an immunosuppressive but nonmyeloablative transplant

regimen consisting of Alemtuzumab, low-dose irradiation, Sirolimus, Cyclophosphamide, and Pentostatin in patients with SCD.

Sickle Cell Disease

Sickle cell disease (SCD) is a well described genetic disorder associated with significant morbidity and mortality. It affects one of every 600 African-Americans in the United States alone . The disease is characterized by recurrent vaso-occlusive crises as a consequence of abnormal hemoglobin polymerization in areas of low oxygen tension. As a result, patients develop functional asplenism leading to a high risk of infections from encapsulated organisms, recurrent pain crises, acute chest syndrome, pulmonary hypertension, kidney failure, and neurologic events, as well as sudden death as the most serious consequences of this disease1. More recently, sickle hepatopathy and iron overload have been discovered to increase mortality in patients with SCD, as patients with ferritin >1000 ug/L or direct bilirubin >0.4mg/dL led to significantly decreased survival as compared to patients with ferritin <1000ug/L and direct bilirubin <0.4 mg/dL (see Figures 1 and 2).

Figure 1:

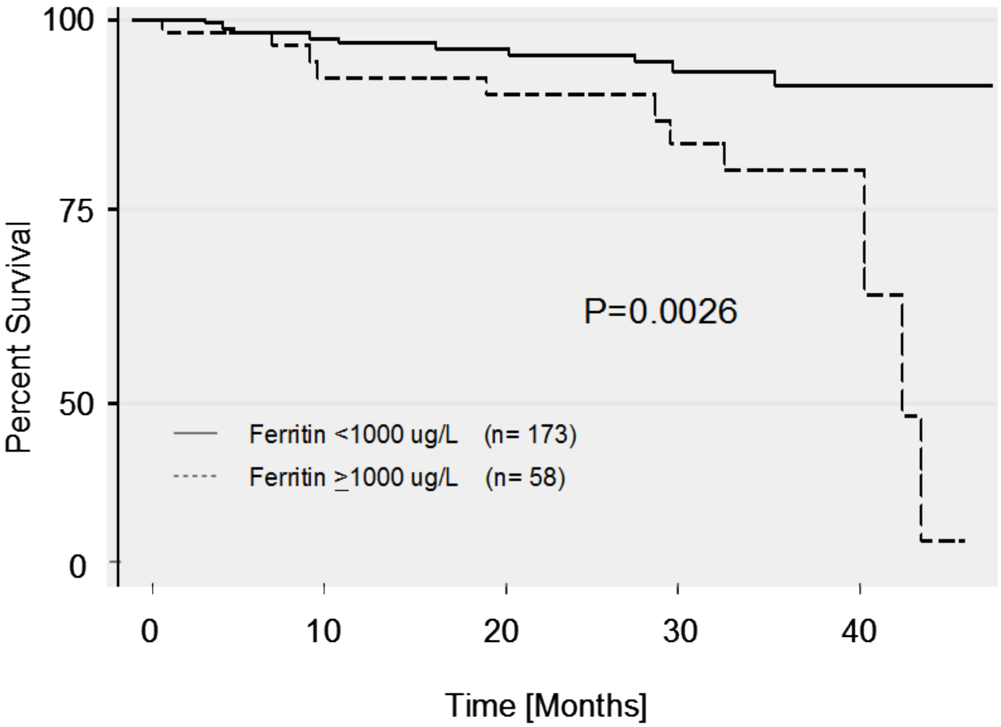

Figure 2:

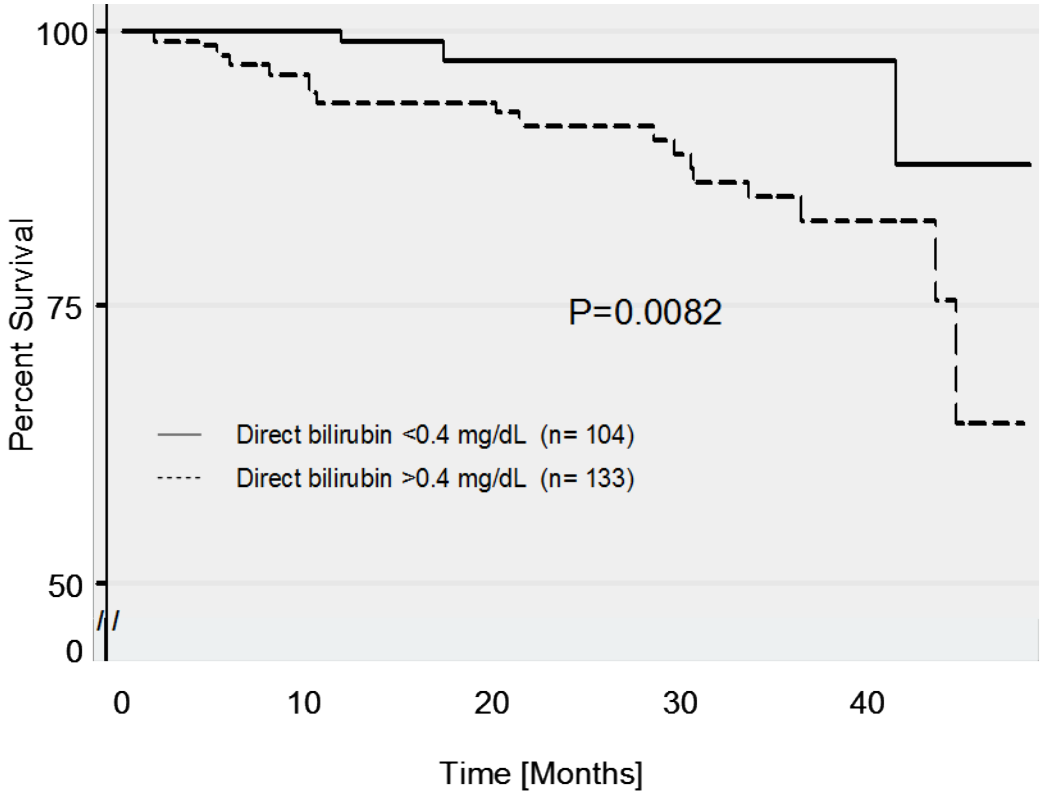

In addition, patients with platelet counts in the lowest quartile of the cohort (<267,000/ul) had a 2.7-fold increase in direct bilirubin after controlling for WBC, to exclude the effect of generalized bone marrow suppression as a cause for thrombocytopenia (OR 2.70 95% CI 1.11-6.56, p=0.029).

The medical costs of this disease are enormous, with estimates of $40,000 per patient per year (year 2000 figures) for chronic transfusion therapy and chelation alone, but do not include the impact on quality of life of those with the disease[3]. An overlapping symptom complex also occurs in patients with the double heterozygous forms of SCD, such as sickle-C, and sickle β-thal^0 disease, and in fact, these patients cannot always be differentiated clinically but only by means of a laboratory test. While transfusions can prevent further neurologic events in patients at risk, iron overload is common, resulting in significant end-organ toxicity. The most common form of treatment in SCD had been erythrocyte transfusions, and more recently hydroxyurea[4-6]. Hydroxyurea results in a significant reduction in the number of painful crises per year and a decreased frequency of acute chest syndrome[5], and has become the treatment of choice for many individuals with SCD. Unfortunately, hydroxyurea is not curative, and does not appear to reverse established end-organ damage.

Several important interventions have led to an improvement in the overall life expectancy of patients with SCD, most notable among these are the use of pneumococcal vaccines and the prophylactic use of penicillin during childhood. Hydroxyurea has also been suggested to improve survival in patients with SCD[7]. However, life expectancy remains significantly shortened compared to the national average with that of an affected male being 47 years versus the national average of 72[1]. There are no factors to predict better survival among patients, further complicating the decision to proceed with higher risk treatments, especially during childhood when such treatments may be better tolerated. In one study of 3,764 patients, 18 percent of the patients died with overt organ failure, and early mortality was highest among patients with symptomatic disease. Importantly however, another 33

percent who appeared to be clinically free of organ failure died during an acute sickle crisis[1].

The only established cure for patients with SCD remains allogeneic bone marrow transplantation; however, the procedure has only been applied to highly selected children[8-10]. In adults, the higher burden of accumulated end- organ damage would be expected to result in higher transplant-associated morbidity and mortality, beyond that reported in children, including seizures and intracranial hemorrhage. As a result, this method has traditionally only been offered to those patients less than the age of 16 with either end-organ damage or symptomatic disease due to their demonstrated higher early mortality rate.

We currently have an ongoing protocol for adult patients with severe congenital anemias and a 6/6 human leukocyte antigen (HLA)-matched sibling donor using a conditioning regimen consisting of Alemtuzumab, 300 cGy total body irradiation (TBI), and Sirolimus for GVHD prophylaxis. The results have been very encouraging, with 87% of transplanted patients being free of their SCD. However, this protocol has been limited by the availability of HLA- matched sibling donors. Of 59 patients with disease severe enough to be eligible for our protocol, only 13 patients (22%) had a 6/6 HLA-matched sibling donor. Due to the inheritance pattern of SCD, the chance that an HLA-matched sibling will be SCD-free further limits the possibility of an appropriate donor. Conversely, the vast majority of patients will have a haploidentical donor as parents, children, and haploidentical-matched siblings can serve as donors. Therefore, two major goals in the application of BMT are to develop a safer conditioning regimen and to increase the donor pool.

SCIENTIFIC AND CLINICAL JUSTIFICATION

In disorders such as hematologic malignancies, the curative effect of bone marrow transplant has been ascribed to the use of myeloablative chemo-radiotherapy and the antileukemic effect of the transplant (the graft-versus- leukemia (GVL) effect)[11]. Unlike patients who undergo allogeneic peripheral blood stem cell (PBSC) transplantation for malignant indications, patients with non-malignant disorders such as SCD do not require full and/or rapid donor engraftment to cure their disease[9, 12-14]. While it is generally accepted that GVHD is less severe in patients conditioned with low intensity preparative regimens[15], graft rejection is preferable to the development of lethal GVHD in the setting of severe SCD.

Our group currently has an ongoing protocol which uses a combination of 300 cGy TBI, alemtuzumab, and sirolimus to transplant high risk patients with sickle cell disease and β-thalassemia who are 4 years of age and older. To optimize our approach, we use peripheral blood stem cells (PBSC) from sibling donors. Preliminary studies, including our own experience with PBSC transplants and low intensity preparative regimens, indicate that transplant-related mortality and severe acute GVHD is uncommon when matched family donors are used[15]. Furthermore, we have established the safety of PBSC mobilization in individuals with sickle cell trait (SCT), likely to represent a sizable fraction of sibling donors for patients with SCD[16]. Additionally, for our patients with SCD, we maintain higher platelet counts[17-19]. Patients with SCD who are not routinely transfused for their therapy undergo exchange transfusion prior to transplant to lower their hemoglobin S to less than 30%[8].

As of August 2, 2016, forty-three patients with SCD have been transplanted. Thirty-eight patients (88%) had long- term stable engraftment and are free from SCD. None of the patients who have maintained their grafts have experienced any sickle cell-related events. No patients experienced GVHD.

We have identified in the HLA-matched sibling setting that female recipients with male donors have a higher graft rejection rate as compared to the overall graft rejection rate of 10-15% (success rate of 85-90%); 0% in sex-matched pairs, 14% in female-into-male recipient, and 50% in male-into-female transplant combination (P=0.0164 Fisher's exact test, male-to-female compared to all other combinations together). A higher graft rejection rate in the setting of male-into-female pairs has been

observed in murine and human transplant settings[20, 21]. Thus with our low intensity approach using alemtuzumab, 300 cGy TBI, and sirolimus, antigens on the Y chromosome and perhaps other minor HLA antigens may represent an important immunologic barrier to overcome. Therefore, pre-conditioning with pentostatin and oral cyclophosphamide (PC) was added (protocol 14-H-0077), and to date, 4 female recipients with male donors remain successfully engrafted.

The main limitation for our HLA-matched sibling protocol has been donor availability. Two hundred eighty seven patients with SCD severe enough to be eligible for the protocol were screened, and, only 102 patients (36%) had a 6/6 histocompatible sibling donor. However, many of those patients were referred to the NIH because they were already known to have an HLA-matched sibling. Nineteen more patients did not qualify due to major ABO mismatch. Our goal is to increase the donor pool so that more patients will have access to this potentially curative therapy. We previously initiated an IRB-approved protocol to establish the feasibility of matched unrelated donor (MUD) and umbilical cord blood (UCB) HSCT. Ten patients who met all study criteria on full screening for our sibling matched HSCT protocol but who did not have a suitable donor were selected for alternative donor searching in the National Marrow Donor Program (NMDP) and Bone Marrow Donors Worldwide (BMDW). We found that only one patient had a greater than 1% probability of having a 6/6 HLA match according to haplogic. Also, only a median of one suitable (>2.5 x 10^7 total nucleated cells per kilogram body weight and ABO-matched) UCB unit was available per patient[22]. As our sibling-matched HSCT protocol has been successful, we are now interested in developing a safe haploidentical protocol for adult patients with SCD so that parents, children, and haploidentical-matched siblings can serve as donors.

Haploidentical HSCT is invariably associated with an increased risk of graft rejection and GVHD. Therefore, as SCD is generally a chronic illness, this modality has only rarely been reported[23-27], as previously stated the risks of transplantation in the haploidentical setting has been felt to outweigh the benefits. However, since adult sickle cell patients with risk factors such as pulmonary hypertension[28, 29], sickle-associated liver disease[2], and frequent hospitalizations for pain crises[1, 30], experience early mortality, and we have had no evidence of GVHD, significant morbidity, or mortality in patients with a matched sibling donor, we believe that the potential benefits outweigh the risks. Given the lower toxicities seen with nonmyeloablative regimens, the possibility of cure with mixed white blood cell chimerism, and the potential improvement of SCD after transplant with even autologous recovery, the extension of low-intensity allogeneic peripheral stem cell transplants to those with severe SCD who would normally be offered a standard transplant, but are considered at a higher risk due to their age (i.e. 16 years of age or older) or other comorbidities is justified. Our conditioning regimen was designed for slow engraftment and tolerance induction resulting in partial to full white blood cell chimerism with reduced rates ofGVHD.

In order to develop a regimen with potential application to individuals with varying degrees of organ dysfunction such as those with SCD, we have sought conditioning which could be applied in such a context, avoiding renally excreted drugs, and relying on the immunosuppressive effects of TBI as the basis for such an approach. Patients with SCD who have been frequently transfused may be at an increased risk for graft rejection as compared to patients with hematologic malignancies because frequent exposure to blood products may lead to donor HLA sensitization. A modest increase in the dosage of TBI from 300 cGy used in our HLA-matched sibling protocol to 400 cGy may increase both the degree of myelosuppression and immunosuppression without significantly altering the side effect profile.

As in our HLA-matched sibling protocol, immunosuppression with the lymphocyte depleting agent Alemtuzumab will be employed. Alemtuzumab is a humanized monoclonal antibody directed against CD52 (which is abundantly expressed on all human lymphocytes), and causes T cell activation *in vitro* as well as complement mediated lysis and antibody dependent cellular toxicity. As a result, it depletes both T and B cells efficiently *in vivo*. It is currently being used in clinical trials as

monotherapy for certain autoimmune disorders including rheumatoid arthritis and multiple sclerosis[31-34], T and B cell malignancies[35], treatment of solid organ rejection[36-38], and has been approved for use in chronic lymphocytic leukemia, a B cell malignancy, as a result of its profound immunosuppressive properties[39-41]. More recently, alemtuzumab has been used prospectively to prevent graft rejection in human allotransplantation[42-46]. Thirty- one patients have been transplanted using 20 mg of alemtuzumab on Day 0 and +1 of transplantation in combination with low-dose CSA, which has been shown to be ineffective when used alone. At 21 months follow-up, only two grafts were lost to rejection[38]. Further, data suggest that the use of alemtuzumab, as compared to fludarabine, reduces the risk of GVHD, even in the unrelated donor setting[45]. In one study of 44 patients, including eight patients receiving unmanipulated marrow from matched unrelated donors who would therefore be at very high risk for developing GVHD, only two patients had acute GVHD, both of which were grade 2. Only one patient developed chronic GVHD[42]. Follow-up to this study has included a further 39 patients undergoing unrelated BMT (including patients having failed a prior transplant and/or having a mismatch in either HLA class I or II alleles) for a total of 47 patients, with only three patients developing Grade III to IV acute GVHD, and none developing chronic extensive GVHD[45]. Other studies have shown that as compared to regimens employing such agents as antithymocyte globulin, calcineurin inhibitors, and methotrexate, alemtuzumab- containing regimens were associated with a decreased risk of severe GVHD[47-49]. In another study of 12 high risk patients undergoing haploidentical BMT, one patient experienced Grade II and one patient developed Grade III GVHD; none developed Grade IV GVHD[46]. This reduced risk of GVHD, as well as its immunosuppressive properties, appears to be due to an in vivo T cell depleting effect on the incoming graft. Unlike ATG, which is a nonspecific antibody directed against lymphocytes and is also used in conditioning regimens; alemtuzumab is also better tolerated and has no risk of causing serum sickness.

To improve the odds for graft acceptance without GVHD, we will employ sirolimus instead of the conventional agent, CSA, based on sirolimus' distinct properties as a tolerogenic agent[50]. Sirolimus is an immunophilin drug similar to CSA; however, unlike CSA, which inhibits the phosphatase calcineurin and therefore prevents the production of interleukin 2 (Il-2), sirolimus prevents translation of mRNAs encoding cell-cycle regulators. As a result, sirolimus only inhibits the ability of lymphocytes to proliferate in response to Il-2. Powell et al demonstrated that cells cultured and stimulated in the presence of sirolimus became anergic, while cells cultured in the presence of CSA did not[50]. These results were also confirmed in our lab using an in vivo model. In a series of experiments performed by Hale et al., sirolimus also proved superior to CSA at prolonging skin graft survival in Class I and class II disparate, fully mismatched, and xenogeneic recipients, and the use of sirolimus was superior to CSA when added to antilymphocyte globulin and bone marrow in a murine model. Further, mice receiving sirolimus accepted a second same donor skin graft, but rejected third party grafts, demonstrating the development of tolerance[51-53]. Sirolimus has also been employed in the bone marrow transplant setting, with matched and mismatched sibling and unrelated donors[54, 55]. One study involving 41 patients with hematologic malignancies used sirolimus, tacrolimus, and methotrexate as GVHD prophylaxis. All evaluable patients engrafted, and grades 0-I, II, III, and IV acute GVHD occurred in 75%, 13%, 8%, and 5% of patients, respectively[54]. Another study included 14 evaluable patients with hematologic malignancies, and used sirolimus and mycophenolate mofetil as GVHD prophylaxis. All patients engrafted, and grades II-IV acute GVHD occurred in 21%, and chronic GVHD in 30% ofpatients[55].

Sirolimus also has less renal toxicity as compared to CSA. A randomized trial comparing the addition of sirolimus at either 2 or 5 mg vs. azathioprine to CSA and prednisone for prophylaxis of renal allograft rejection showed a significantly lower rate of acute rejection at both doses of the sirolimus as compared to azathioprine (16.9% and 12.0% vs. 29.8%)[56]. In a similar study comparing sirolimus vs. CSA as adjuncts to azathioprine and prednisone, there were similar rates of graft survival and incidence of biopsy confirmed graft rejection (98% vs. 90% and 41% vs. 38%, respectively), but significantly lower serum creatinines in the sirolimus group[57]. Moreover, in renal transplant studies,

sirolimus has been shown to be equally effective in preventing graft rejection and has been approved as an alternative to CSA.

Cyclophosphamide given from two to three days after bone marrow transplantation has been shown to facilitate engraftment and prevent the development of GVHD by targeting activated lymphocytes[58-61]. Colson *et al* have shown that post-transplantation high dose cyclophosphamide (200mg/kg) facilitated engraftment of MHC- incompatible marrow grafts in mice after nonmyeloablative conditioning[60, 61]. Post-transplant cyclophosphamide has been shown to facilitate engraftment in a partially HLA-mismatched setting. Thirteen patients with high-risk hematologic malignancies were conditioned with fludarabine, 200 cGy TBI, cyclophosphamide 50 mg/kg on day +3, mycophenolate mofetil, and tacrolimus[58]. Eight patients (62%) experienced sustained donor cell engraftment, as compared to 3% when a similar regimen in which cyclosporine replaced tacrolimus in the absence of post- transplant cyclophosphamide was used. Post-transplant cyclophosphamide has also been reported to decrease the incidence of GVHD in a mismatched major histocompatibility complex murine model, in which median survival increased from 25 to 145 days in mice that did not versus did receive post-transplant cyclophosphamide, respectively[59]. Further, an ongoing haploidentical SCT protocol at Johns Hopkins Hospital for patients with nonmalignant diseases including severe SCD employs pre-transplant cyclophosphamide, fludarabine, 200cGy TBI, 50mg/kg cyclophosphamide which is administered 3 and 4 days post-transplant (100mg/kg cumulative dose), and mycophenolate mofetil and tacrolimus or sirolimus for GVHD prophylaxis[62, 63]. To date, 14 patients with SCD have undergone haploidentical SCT, and 7 have experienced reversal of their SCD. Because of the inherent increased risk of GVHD associated with haploidentical donors as compared to HLA-matched sibling donors, we explored post-transplant cyclophosphamide in a dose escalating fashion (protocol 09-H-0225, see below).

To determine whether post-transplant cyclophosphamide is beneficial in the setting of low-dose TBI and T cell depletion, we embarked upon a series of experiments in the murine model. We first sought to determine the optimal irradiation dose to explore post-transplant cyclophosphamide. We applied our regimen using mismatched strains, where Balb/C mice served as donors and C57Bl6 mice were recipients. Recipient mice received conditioning with a pan-lymphocyte suppressive agent, anti-thymocyte serum, rapamycin, and doses of TBI ranging from 100 to 400cGy to determine the level of irradiation necessary for engraftment. From this experiment, we determined that donor white blood cell chimerism levels increased from 10% in mice that received 100cGy to as high as 50% in mice that received 400cGy. We therefore increased the TBI dose from 300cGy to 400cGy in order to increase the myelosuppression and immunosuppression that may be needed in the haploidentical setting.

Since post-transplant cyclophosphamide is thought to prevent graft rejection and GVHD by deleting alloreactive lymphocytes, we next sought to determine whether cyclophosphamide would be effective in the setting of lymphocyte depletion. Therefore, mice received a lymphocyte-depleting agent, thy-1.2 monoclonal antibody, 400cGy TBI, rapamycin, and doses of post-transplant cyclophosphamide ranging from 0 to 200mg/kg. The level of engraftment did not vary significantly between mice that received cyclophosphamide from 0 to 100mg/kg, with donor myeloid chimerism levels of about 55%. However, mice that received 200mg/kg only achieved donor myeloid chimerism levels of 35% at 35 weeks post-transplant. Further, mortality increased at cyclophosphamide doses above 50mg/kg. In our next series of experiments, we evaluated whether lymphocyte proliferation was sufficiently decreased by rapamycin to inhibit cyclophosphamide's effects post-transplant. We found that rapamycin and post-transplant cyclophosphamide are synergistic, since mice that received 200cGy and either rapamycin or cyclophosphamide failed to engraft, while all mice that received both agents engrafted, with donor white blood cell chimerism levels ranging from 10-60%[64]. Further, since we demonstrated that rapamycin and post-transplant cyclophosphamide are synergistic, but may not be beneficial in the setting of profound lymphocyte depletion, which occurs with alemtuzumab, we added cyclophosphamide in a dose-escalating fashion if the first cohort of patients experienced graft

rejection or severe GVHD. The treatment protocol therefore included 400cGy TBI, alemtuzumab, sirolimus, and if necessary due to the development of graft rejection or severe GVHD in our patients, post-transplant cyclophosphamide in a dose-escalating fashion.

Pharmacokinetic evaluation of cyclophosphamide and its metabolites, such as 4-hydroxycyclophosphamide and 2- dechloroethylcyclophosphamide, have been performed[65-69]. However, sufficient data do not exist in patients who receive cyclophosphamide post-PBSCT. Various genotyping assays for single nucleotide polymorphisms in CYP450 genes (for example CYP3A4/5, CYP2B6, and CYP2C9) and in MDR1/ABCB1 (for example C3435T) have been shown to affect the clearance of cyclophosphamide[66, 67, 70]. Differences in cyclophosphamide clearance in African-American as compared to Caucasian patients are thought to be related to genotypic differences[67]. Further, studies performed in patients with renal insufficiency show that decreased kidney function alters cyclophosphamide clearance[66, 71]. Lastly, studies have not evaluated whether cyclophosphamide clearance affects the incidence of graft rejection in patients who administer cyclophosphamide post-transplant.

As of August 17, 2016, 23 subjects have been enrolled on the parent haploidentical transplant study (09-H-0225) as part of 3 sequential cohorts. All subjects received alemtuzumab, 400 cGy TBI in divided doses 1 and 2 days before transplant, and sirolimus. The first 3 subjects transplanted did not receive cyclophosphamide per protocol. One of the 3 subjects engrafted, and the 3rd subject lost her graft by 7 months post-transplant (see Table). No subject experienced viral reactivation or fungal infection peri- transplant. One subject died 5 years post-transplant from an infectious surgical complication. Stopping rules for graft rejection were met and the study was advanced to the 2nd cohort where 1 dose of cyclophosphamide was added at 50 mg/kg IV on day 3 post-transplant, and 8 subjects were transplanted (including 2 with β-thalassemia). Five subjects engrafted, and 2 remain free of SCD long-term. One experienced Grade I acute GVHD. One subject experienced disseminated adenovirus at approximately 15 months post-transplant with positive nasopharyngeal wash, transaminitis, and detection of adenovirus in the blood. However, the adenovirus quickly cleared spontaneously. One subject received a platelet transfusion that was contaminated with bacteria and died of sepsis at approximately 6 months post-transplant. Two subjects developed pulmonary lesions that were suspicious for fungus, and they were managed well with anti-fungal therapy. And one subject experienced acute EBV infection and post-transplant lymphoproliferative disorder which was effectively treated with rituximab. Stopping rules for graft rejection were again met, and the study was advanced to the 3rd cohort where 2 doses of cyclophosphamide were given each at 50 mg/kg IV on days 3 and 4 post-transplant. Twelve subjects were transplanted, and 10 engrafted. Six of the 12 subjects remain free of SCD. One subject experienced Grade I acute GVHD, and one other limited chronic GVHD. Two subjects have experienced chronic EBV viremia without symptoms and have not required treatment. Five (including 1 who experienced EBV viremia) have experienced CMV reactivation (including 1 with CMV disease) and all were successfully treated with foscarnet. One subject was treated for a presumed fungal lesion. One subject who rejected his graft died 3 years post-transplant from pulmonary hypertension and diastolic dysfunction. Stopping rules for the 3rd cohort were reached, and accrual to the protocol has ceased. While the engraftment rate and success rates have improved with each successive cohort, the graft rejection rate remains unacceptably high, Therefore, we seek to add additional immunosuppressive therapy.

**Cohort**	**Engraftment Rate**	**Success Rate**	**GVHD**		**Viral Reactivation**	**Viral Disease**
			Acute	Chronic		
1 (no PT-Cy)	1/3 (33%)	0/3 (0%)	None	None	0	0
2 (50mg/kg PT-Cy)	5/8 (63%)	2/8 (25%)	1 (Grade 1)	None	1	1

3 (100mg/kg PT-Cy)	10/12 (83%)	6/12 (50%)	1 (Grade 1)	1 (limited)	5	1

While the engraftment and success rates improved with the addition of post-transplant cyclophosphamide, the graft rejection rate remains unacceptably high. Therefore, additional immunosuppression is likely necessary to overcome the stronger host-versus-graft barrier. Pentostatin is an FDA-approved medication that is primarily used for the treatment of hairy cell leukemia[72] and chronic lymphocytic leukemia[73, 74]. Pentostatin, which has a unique mechanism of action that involves inhibition of the enzyme, adenosine deaminase (ADA), that is deficient in a large proportion of patients with severe combined immune deficiency (SCID), also mediates significant immune suppression and immune depletion[75]. The immune modulating effects of pentostatin, which comprise a significant side effect in cancer therapy, can be harnessed in the transplantation setting to prevent graft rejection. An initial study by Pavletic et al demonstrated the lymphodepleting effects and safety of pentostatin (a total of 12 mg/m^2 over three days) when administered in sequence with nonmyeloablative doses of TBI (200 cGy) in the context of allogeneic HSCT[76]. In another study, pentostatin (a total of 8 mg/m^2 over two days) was safely administered in sequence with 600 cGy of TBI prior to allogeneic HSCT[77]. As such, pentostatin can represent an effective component to host conditioning regimens for the purpose of preventing graft rejection. However, to date, pentostatin has been used less frequently than other immune modulating conditioning, which has primarily involved the purine analogue fludarabine.

In murine transplantation models, our NCI colleagues set out to compare the immune depleting and immune suppressive effects of pentostatin relative to fludarabine[78]. They found that pentostatin was advantageous relative to fludarabine because at a given level of host myeloid cell depletion, there was a significantly greater magnitude of host B and T cell depletion. In addition, host T cells that remained after pentostatin-based conditioning were significantly more immune suppressed relative to host T cells remaining after fludarabine-based conditioning. Importantly, they found that optimal host immune modulation was achieved when intermittently dosed pentostatin was administered in combination with daily dosing of cyclophosphamide. These results indicated that, pentostatin, which can have a biological half-life (in terms of inhibiting the ADA enzyme) of up to one week, is best administered in combination with low-dose therapy with DNA alkylators to achieve maximal immune depletion while minimizing myeloid cell depletion. Finally, they found that host recipients of pentostatin/cyclophosphamide (PC) conditioning were significantly less likely to reject a fully MHC-disparate hematopoietic cell transplant relative to recipients of fludarabine/cyclophosphamide conditioning.

Based on these murine results, a pilot clinical trial of PC conditioning in the setting of HLA-matched sibling allogeneic HCT was initiated at the NIH Clinical Center (NCI Protocol 08-C-0088; Low Intensity Allogeneic Hematopoietic Stem Cell Transplantation Therapy of Metastatic Renal Cell Carcinoma Using Early and Multiple Donor Lymphocyte Infusions Consisting of Sirolimus-Generated Donor Th2 Cells). In an attempt to maximize host immune depletion with relative sparing of host myeloid cells, they designed a 21-day pre-transplant host conditioning regimen consisting of a total of 12 mg/m^2 of pentostatin administered in equal doses on days 1, 8, and 15 of the 21- day regimen; and, in this context, patients received a flat, daily dose of cyclophosphamide at the relatively modest dose of 200 mg per day, each day of the 21-day regimen. However, to ensure sparing of host myeloid cells, they also designed a dose adjustment scheme whereby daily doses of cyclophosphamide would be held in the event that an ANC value were to be reduced. Using this regimen, in 12 consecutive patients, they achieved the protocol-defined target level of immune depletion (end of regimen ALC value of < 200) without a single case of significant neutropenia (no case of grade 3 toxicity). And, each of the 12 patients achieved prompt alloengraftment without any case of graft rejection. These results indicate that the PC regimen can be safely administered and is effective when used alone for the prevention of graft rejection.

In other murine models, the NCI team next evaluated the ability of the PC conditioning to abrogate host immune responses against foreign protein, namely the anti-cancer immunotoxin SS1P[79]. Immunogenicity of foreign protein therapies is a significant clinical obstacle, as many patients are limited to only a single course of protein therapy due to neutralizing antibody formation; such antibody formation is induced by B cells, with significant cooperativity from host T cells. As such, they hypothesized that the PC regimen might be particularly effective for preventing host immunogenicity to SS1P. Indeed, in these murine studies, they found that the PC regimen safely modulated host immunity and allowed for up to six cycles of immunotoxin therapy without induction of neutralizing antibody formation. Based on these results, they initiated a clinical translation of SS1P therapy preceded by host immune modulation with the PC regimen (NCI Protocol 11-C-0160; A Pilot Study of Pentostatin Plus Cyclophosphamide Immune Depletion to Decrease Immunogenicity of SS1P in Patients with Mesothelioma). To improve the feasibility of the regimen, this protocol is evaluating a somewhat truncated version of the PC regimen, with pentostatin currently being administered at a dose of 4 mg/m^2 (on days 1, 5, 9, and 13) with daily, dose-adjusted cyclophosphamide being administered on days 1 through 14 of the regimen. Initial results from the first 10 patients treated on this protocol indicate that the PC regimen indeed is safe and is effective in preventing or delaying host immune responses against the foreign protein therapy. In combination, the results of their studies in the allogeneic and autologous setting indicate that the dose-adjusted PC regimen can be safely administered and yield effective immune modulation. In sum, these results suggest that adding the PC regimen may represent an effective modality to reduce graft rejection in the setting of allogeneic HSCT in the population with SCD.

An important objective of the current protocol will be to evaluate the safety and efficacy of the PC regimen when administered over a 14-day interval. The patients with SCD and HLA-matched sibling donors (protocol 14-H- 0077) tolerated alemtuzumab, 300cGy TBI, and PC conditioning (with a pentostatin dose of 2 mg/m^2/day which has been shown to be effective in the lymphoid neoplasia setting[74]) well and did not experience viral disease. To maximize safety of the current protocol, the PC therapy will be administered separately and followed by the alemtuzumab and TBI. Because the patients tolerated PC at 2 mg/m^2/day well and the target ALC <100/uL was not reached, we will increase the pentostatin to 4 mg/m^2/day due to the more robust host-versus-graft barrier in the haploidentical setting. Further, the TBI dose will be increased to 400cGy to provide additional myelosuppression and immunosuppression. Lastly, because post-transplant cyclophosphamide increased engraftment in the haploidentical setting and potentially protected the patients from severe GVHD, 50 mg/kg cyclophosphamide will be given intravenously 3 days post- transplant. Because of the higher incidence of viral reactivation seen in cohort 3 of protocol 09-H-0225 where a total of 100 mg/kg cyclophosphamide was given post-transplant, a second dose of cyclophosphamide will not be administered. As alemtuzumab, pentostatin, pre- and post-transplant cyclophosphamide, and sirolimus will be given, the incidence of viral disease could potentially be higher as compared to the incidence seen in our previous protocol. Therefore, stopping rules have been included to stop the study if the incidence of viral disease is unacceptable (see section 15).

As of December 20, 2020, 19 patients have been transplanted on this protocol, and 18 have at least 100 days of follow-up. Of the 18 patients where the primary endpoint can be evaluated, 17 are alive and 16 engrafted. However, 4 of the 16 patients have had slowly falling donor chimerism levels over time. We recently reported that due to vast differences in red blood cell survival between donor and recipient, 20% donor myeloid chimerism (DMC) is sufficient to reverse SCD[80]. One patient with 16% DMC had return of his SCD with jaundice and hospitalization for an acute painful crisis at 2.5 years post-transplant. Another patient has a DMC level of 19% at 18 months post-transplant. His HbS is 57% (donor HbS 37%) and his hemoglobin is 9.2 g/dL. He does not have signs of hemolysis or acute manifestations of his SCD at this point but we expect that his symptoms will return soon. The other 2 patients are hovering above the 20% DMC threshold and have not yet experienced return of their SCD; both patients are >18 months post-transplant. Therefore, while acute graft rejection has

improved substantially on this protocol, suggesting that acute rejection was indeed due to insufficient immunosuppression on our old haploidentical protocol late graft failure remains a problem.

As the patients on this protocol are experiencing slowly falling donor chimerism levels over time, inadequate myelosuppression may be contributing. Indeed, 3 patients transplanted on our old haploidentical protocol (09-H-0025) or our HLA-matched sibling protocol (03-H-0170) and had falling donor chimerism levels with return of their SCD were re-transplanted with the same donor, under protocols 14-H-0111, 14-H-0130, and 03-H-0170 using busulfan (~10 mg/kg) to increase myelosuppression and half the dose of alemtuzumab (0.5 mg/kg). All 3 patients engrafted and with a follow-up of >3 years, all 3 patients have 100% DMC, close to 40% HbS, and are free from SCD. Patients with insufficient donor chimerism and return of SCD have the option to enroll on a repeat transplant protocol (19-H-0118) to be re-transplanted with the busulfan-containing regimen.

This protocol originally included 400cGy TBI given in 2 fractionated doses. In order to increase the donor stem cell competitive advantage and to inhibit recipient stem cell recovery, we are going to give 400cGy TBI in 1 fraction instead of 2. We do not believe this change will affect the primary endpoint which is evaluated at 100 days post-transplant. Recently, Bolanos-Meade and colleagues published a manuscript where they used the same regimen for patients undergoing haploidentical transplant except that they increased the TBI dose from 200cGy to 400cGy as a single dose in adults and children with SCD[81]. The graft rejection rate decreased from 43% with their original regimen[63] to 8%. The Vanderbilt Haploidentical Consortium also uses a 400cGy single dose of TBI in patients with beta thalassemia with similar efficacy[82].

As of August 16, 2021, 2 patients developed Evans syndrome that was refractory to multiple immunomodulatory agents and splenectomy. Both patients developed a severe hyperinflammatory reaction that led to multi-organ failure that was ultimately fatal. Due to the unexpected severity of this transplant-associated complication, the protocol has been amended (Amendment R) to enroll adults with either disease severe enough to be associated with early mortality or progressive cerebrovascular disease where the potential benefits of this protocol outweigh the risks. As we have already been targeting a population of patients with severe SCD, 16 of the first 19 patients transplanted would have been eligible for transplant even with these new eligibility criteria.

Patients with donor specific HLA-antibodies (DSA) have traditionally been excluded from our protocol because of the known increased risk of graft rejection in the haploidentical setting. Primary graft failure and delayed engraftment have been associated with DSA83. The incidence of primary poor graft function is higher in patients with DSAs ≥2,000 MFI compared to those with a DSA <2,000 MFI84. One hundred percent neutrophil engraftment was noted among the 316 transplant recipients with either negative DSA or DSA <2,000. Transplant recipients with <2,000 MFI DSA may therefore proceed with transplant without desensitization.

STUDY DESIGN

A haploidentical relative donor will receive filgrastim (G-CSF) 10 to 16 µg/kg/d subcutaneously or intravenously for up to 6 days with apheresis collections of peripheral blood hematopoietic progenitor cells (PBPC) after the 5th day (and after the 6th day if required). The product will be collected and frozen at least two weeks prior to the recipient beginning his/her conditioning.

Pentostatin will be given on days -21, -17, -13, and -9 and oral cyclophosphamide from days -21 to -8, with the intention to be administered in the outpatient setting. Each SCD patient will receive a preparative regimen of Alemtuzumab to be infused on days –7 to –3, followed by 400 cGy TBI delivered in one fraction on day -1. The PBPC graft (Appendix C) will be infused on day 0 and day +1 as needed. Cyclophosphamide will be given at 50 mg/kg on day +3. Sirolimus will be started at a loading dose of 5mg PO q4h x three doses at one day after the completion of cyclophosphamide (on day +4) and continued the following day at 5mg PO q24h to maintain trough levels between 5-15

ng/ml. On days +14 (or when subject starts to engraft), +30, +60, +100, 6 months, 12 months, 18 months, 24 months, and annually thereafter, the chimeric status of SCD patients will be assessed by microsatellite analysis of the peripheral blood. More frequent monitoring may be required.

The design of the study incorporates the following features:

This is a phase I/II pilot study to determine the safety and therapeutic potential of a new transplant approach (disease-free survival, overall survival) and to evaluate its toxicity profile (immediate toxicity, graft-versus-host disease, graft rejection, mortality, incidence of viral reactivation and disease) in a patient population with SCD.

The SCD patient cohort to be studied: Those patients with severe SCD who have risk factors for high mortality and morbidity related to their disease (see inclusion criteria in section 5.1)

Nonmyeloablative Transplant Conditioning Regimen - Immunosuppression without myeloablation: SCD patients will receive conditioning sufficient to allow donor lympho-hematopoietic engraftment without complete marrow ablation. If the graft is rejected, the patient should reconstitute autologous marrow function. We will use a combination of low-dose irradiation (400 cGy TBI), alemtuzumab, pentostatin, cyclophosphamide and sirolimus.

Peripheral blood hematopoietic progenitor cell (PBPC) transplant: An unmanipulated peripheral blood stem cell collection from a filgrastim (G-CSF) stimulated haploidentical relative donor should improve the chance of engraftment because of the high stem cell dose ($\geq 5 \times 10^6$/kg CD34+ cells) and the presence of donor lymphocytes. To further reduce the risk of graft rejection and GVHD, SCD patients will receive pentostatin and cyclophosphamide before the transplant and sirolimus and cyclophosphamide after the transplant.

PATIENT SELECTION

Inclusion criteria- recipients (must fulfill one disease category in 5.1.1 and all of 5.1.2)

Patients with any type of sickle cell disease who are at high risk for disease-related cerebrovascular morbidity or early mortality, defined by having severe end-organ damage (A, B, C, D, or E):

A neurologic event resulting in focal neurologic deficits that lasted ≥ 24 hours (classical clinical definition of stroke, not requiring imaging studies of the brain) **<u>OR</u>** a focal neurological event resulting in abnormalities on T2- weighted or FLAIR images using an MRI scan, indicative of an acute infarct, with no other reasonable medical explanation (definition of a stroke supported with MRI imaging scans of the brain), **<u>OR</u>** both. ; **OR**

Tricuspid regurgitant jet velocity (TRV) of ≥ 2.7 m/s[28, 29] at baseline (without vaso-occlusive crisis) and/or pulmonary hypertension; **OR**

Sickle hepatopathy defined as either ferritin >1000 mcg/L and platelet count < 250,000/uL (without vaso-occlusive crisis) OR direct bilirubin > 0.4 mg/dL and platelet count <250,000/uL (without vaso-occlusive crisis)[2]

Any acute chest syndrome episode resulting in intensive care admission requiring non-mechanical ventilatory support: simple nasal cannula, face mask that requires oxygen content (venti mask, non-rebreather), continuous positive airway pressure (CPAP), Bilevel positive airway pressure (BiPAP), high flow nasal cannula (HFNC) or invasive mechanical ventilatory support (delivered by endotracheal tube or tracheostomy)[85].

Silent cerebral infarct defined as an infarct-like lesion based on an MRI signal abnormality at least 3 mm in one dimension and visible in two planes on FLAIR or T2- weighted images (or similar image with 3D imaging) and documented neurological examination performed by a neurologist demonstrating the participant has a normal neurologic examination or an abnormality on examination that could not be

explained by the location of the brain lesion(s)[86, 87].

Non-disease specific:

Age ≥18 years

Haploidentical relative donor available

Ability to comprehend and willing to sign an informed consent

Negative serum β-HCG

Ejection fraction ≥ 35%

Glomerular filtration rate >60 mL/min/1.73m^2 by cystatin C-based or iothalamate or iohxol-based or other equivalent GFR testing (see 6.3)

Adjusted DLCO ≥ 35%

Exclusion criteria –recipient (any of the following would exclude the subject from participating)

Available 6/6 HLA-matched sibling donor

ECOG performance status of 3 or more (See Appendix A)

Evidence of uncontrolled bacterial, viral, or fungal infections (currently taking medication and progression of clinical symptoms) within one month prior to starting the conditioning regimen.

Patients with fever or suspected minor infection should await resolution of symptoms before starting the conditioning regimen.

Major anticipated illness or organ failure incompatible with survival from PBSC transplant

Pregnant or breast-feeding

Donor specific anti-HLA antibodies (DSAs) ≥2000 Mean Fluorescence Intensity (MFI)

Patients seronegative for EBV who have EBV seropositive donors

Inclusion criteria – donor

Haploidentical relative donor deemed suitable and eligible, and willing to donate, per clinical evaluations who are additionally willing to donate blood for research. Related donors will be evaluated in accordance with existing Standard NIH Policies and Procedures for determination of eligibility and suitability for clinical donation. Note that participation in this study is offered to all related donors, but is not required for a donor to make a stem cell donation, so it is possible that not all related donors will enroll onto this study.

Exclusion criteria- donor

(None)

TREATMENT PLAN

Sickle cell patients with pulmonary hypertension will meet with a Pulmonary Medicine Consult to determine appropriate management prior to SCT.

Hydroxyurea must be discontinued at least one day prior to initiating the PC regimen to avoid overlapping myelosuppression. Other sickle cell-specific therapy and iron chelation must be discontinued > 48 hours before initiating the conditioning regimen.

6.1 HLA Typing

A haploidentical donor shall be selected which shares one haplotype in common with the recipient

such that HLA compatibility will be a minimum of 5 out of 10 HLA loci matched. The HLA loci to be tested will be HLA A, B, Cw, DRB1, and DQB1. Decisions may be based on degree of HLA mismatch between donor and recipient. However, other criteria will be considered as well including donor age, number of pregnancies, CMV status, and blood type. Donor-recipient pairs will initially be typed molecularly to provide a low resolution typing (antigen-level) to aid in the selection of the potential donor. Upon review of the familial inheritance pattern, a qualified HLA staff member will review haplotype inheritance. After consultation with NHLBI physicians and qualified HLA personnel, a donor(s) will be selected for further testing. High resolution (allele-level) typing will be performed. Final selection of a donor will be in consultation with NHLBI physicians and qualified HLA personnel.

Exchange Transfusion (See Appendix C)

Prior to the plerixafor autologous back up collection and PC regimen, those SCD patients who are not routinely (exchange) transfused will undergo an exchange transfusion per Department of Transfusion Medicine (DTM) procedure for a target HbS <30% just prior to receiving both the plerixafor for back up stem cell collection and the preparative regimen in order to decrease the likelihood of neurologic and other sickling events that may be precipitated by the transplant procedure. Exchange transfusions may be deferred in patients where the risks of the procedure are deemed greater than the benefits as per PI discretion (ie: history of severe transfusion-associated hyperhemolysis or delayed hemolytic transfusion reaction).

Collection of Autologous Peripheral Blood Stem Cells (PBSC, See Appendix D)

Regardless of our low-intensity conditioning regimen, in patients transplanted as of November 2015, 5 of 21 patients with SCD did not recover their platelet count to >50K by 100 days post-transplant. Because of the risk of intracerebral hemorrhage in patients with SCD and severe thrombocytopenia, backup collection of autologous stem cells will be performed. G-CSF has been associated with extensive morbidity (including vaso-occlusive crises, acute chest syndrome, and multi-organ failure) and mortality in patients with SCD[88]. However, as of 4/1/16, >40 subjects with sickle cell trait have undergone mobilization with G-CSF at the NIH without any events that would suggest a vaso-occlusive crisis or any other sickle-related complication. Further, a recent study compared 12 subjects with sickle cell trait and 12 matched controls who received G-CSF[89]. There was no significant difference in the rate of adverse events between the 2 groups. Bone marrow harvest has not been studied well in patients with SCD. The risk of general anesthesia may be significant in patients with severe disease, and fluid shifts associated with removal of bone marrow may also lead to significant morbidity. A protocol involving plerixafor-mobilized PBSCs for patients with SCD recently completed enrollment (17-H-0124). 15 patients at the NIH and St. Jude's received 240 μg/kg plerixafor to collect autologous PBSC. With the exception of prolonged hospitalization for pain, the patients tolerated plerixafor well without any unexpected adverse events. Other reports have recently been published describing the efficacy and safety of plerixafor mobilization for patients with SCD[90, 91]. An additional study reported safety, but insufficient CD34 mobilization, likely due to concomitant hydroxyurea therapy[92]. Therefore, backup autologous cells will be collected in patients with SCD via bone plerixafor-mobilized PBSC collection on this protocol (see appendix D for details). If autologous cells are mobilized and collected under another NHLBI-approved protocol, these cells may be accepted and used per PI discretion for this protocol's purpose

Preparative Regimen

Hydroxyurea will be initiated or continued prior to the conditioning regimen. Hydroxyurea may not be initiated in those patients where the potential risks outweigh the benefits as determined by the PI or designee (for example, concern that hydroxyurea contributes to the development of leg ulcers). These patients will be managed by simple transfusions.

All drugs will be given intravenously if possible based on the dosing formulation. All other

concomitant medications or special procedures noted in the protocol shall follow standard NHLBI transplant protocols/procedures.

All transplant recipients will receive a study calendar prior to the start of the preparative regimen (See Appendix E).

The 14-day PC regimen will be administered just proximal to the alemtuzumab with the intent to be administered as an outpatient. As such, pentostatin will be administered at a dose of 4 mg/m^2 on days -21, -17, -13, and -9. A total pentostatin dose of 16 mg/m^2 will be utilized, which is equal to previous transplantation studies. On the day of and for 2 days after each pentostatin infusion, olanzapine 10 mg PO will be given; the dose may be decreased as needed to prevent or treat symptoms such as sedation. On the day of each pentostatin infusion, 8 mg IV or 16-24 mg PO of ondansetron will be administered 30-60 minutes prior to, and 1 L of 0.9% saline will be infused over 60-120 minutes prior to pentostatin infusion. Pentostatin, diluted in 500 mL of 0.9% sodium chloride, will then be infused IV over 60 +/- 10 minutes. Oral ondansetron, 8mg twice daily, may be administered throughout the 14-day PC regimen. Changes in the anti-emetics, hydration, or administration of pentostatin is allowed at the discretion of the PI or designee.

Although pentostatin is >90% renally excreted, there is no established consensus on how to dose in those with impaired renal functions. Page 2 of pentostatin package insert reports that after a dose of 4mg/m^2, the terminal half- life was measured to be 6 hours in patients with creatinine clearance (CrCl) >60 mL/min vs 18 hours in those with CrCl <50 (http://hemonc.org/docs/packageinsert/pentostatin.pdf). Therefore, subjects with GFR $\leq$60 mL/min will be excluded from the protocol.

Kidney function will be monitored while SCD patients receive PC since pentostatin can lead to renal impairment. Thus, serum creatinine and cystatin C levels will be obtained prior to each dose of pentostatin. Dose adjustment for renal dysfunction will occur as follows:

4 mg/m^2 if CrCl > 60 mL/min/1.73m^2

3 mg/m^2 if CrCl 40-60mL/min/1.73m^2

2 mg/m^2 if CrCl 20-39mL/min/1.73m^2

1 mg/m^2 if CrCl <20 mL/min/1.73m^2

Estimate of creatinine clearance is per cystatin C-based GFR using the CKD-EPI GFR calculation (below) $eGFR = 133 \times \min(S_{cys}/0.8, 1)^{-.0499} \times \max(S_{cys}/0.8, 1)^{-1.328} \times 0.996^{Age} \times$ [0.932 if female]

S_{cys} (standardized serum cystatin C) = mg/L
min = indicates the minimum of $S_{cys}/0.8$ or 1
max = indicates the maximum of $S_{cys}/0.8$ or 1

age = years

or by the Cockcroft-Gault formula (below):

eCcr = (140 – Age) x Mass (in kilograms)* x [0.85 if female] 72 x Serum Creatinine (in mg/dL)

**mass is ideal body weight, not actual*

Cockcroft-Gault formula will be used if cystatin-C based results are not available prior to pentostatin dosing.

Cystatin-C levels may be affected by systemic illnesses such as autoimmune disease[93]. Therefore, to determine eligibility, a more exact approach such as measuring iothalamate or iohexol plasma

clearance may be performed to more accurately measure GFR.

Cyclophosphamide will be administered at a daily dose of 200 mg per day on days -21 through -8 as per the schema detailed below. Cyclophosphamide will be either reduced or omitted depending on whether the lymphocyte depletion target has been achieved and whether there is any myeloid cell toxicity. The stated goal of the PC regimen is to enter the interval of alemtuzumab therapy with substantial immune depletion (absolute lymphocyte count [ALC] value < 100) and without grade 3 neutrophil toxicity (absolute neutrophil count [ANC] value <1000).

Cyclophosphamide dose adjustment based on ALC and ANC values					
Day of cycle[1]		ALC value at time of evaluation	ANC value at time of evaluation[2]		Cyclophosphamide dose[3]
-21		Any	> 1000		200
-17		≥ 400	> 1000		200
		200-399	500-999		100
		< 200	< 500		0
-13		≥ 200	> 1000		200
		100-199	500-999		100
		< 100	< 500		0
-9		≥ 100	> 1000		200
		50-99	500-999		100
		< 50	< 500		0

[1] *Pentostatin will not be dose-adjusted based on ALC/ANC values.*

[2] *For ANC values <1000, decrease in cyclophosphamide dosing only – patients will not receive G-CSF.*

[3] *Cyclophosphamide dose indicated will be continued daily until the next ALC/ANC measurements.*

Admission, exchange or simple transfusion if necessary.

Days –7 to –3 Alemtuzumab IV given in an escalating dose schedule over a total of 5 days as follows:
Day –7: Diphenhydramine 1mg/kg (maximum 50mg) I.V. and Acetaminophen 10-15mg/kg (maximum 650mg) P.O., then followed 30 minutes later by alemtuzumab 0.03mg/kg in 100mL normal saline infused over 2 hours
Day –6: Diphenhydramine 1mg/kg (maximum 50mg) I.V. and acetaminophen 10-15mg/kg (maximum 650mg) P.O., then followed 30 minutes later by alemtuzumab 0.1mg/kg in 100mL normal saline infused over 2 hours
Day -5 to Day -3: Diphenhydramine 1mg/kg (maximum 50mg) I.V. and acetaminophen 10-15mg/kg (maximum 650mg) P.O., then followed 30 minutes later by alemtuzumab 0.3 mg/kg (with a maximum dose of 30 mg), in 100mL normal saline infused over 2 hours
Day –1: Total Body Irradiation (TBI), dose of 400 cGy delivered as per the Department of Radiology standard of practice

Day 0 and day +1 as needed: Infusion of unmanipulated filgrastim - mobilized peripheral blood stem cells

Day +3 to Day +5: Cyclophosphamide 50 mg/kg/dose will be given on Day +3 by IV infusion over 60 minutes. Dosing will be based on ideal body weight with the exception that if the actual body weight is lower than the ideal body weight, the actual body weight will be utilized for the cyclophosphamide dose calculation. Mesna (which will also be dosed by ideal body weight) and intravenous hydration will be given concurrently with the cyclophosphamide dose as based on current TCT consortium guidelines. The start time of the cyclophosphamide infusion should be within 72 hours of the start time of the stem cell infusion on Day 0.

Sirolimus will be started at 5mg PO q4h x three doses at one day after the completion of cyclophosphamide (on day +4). The first dose of sirolimus should be at least 24 hours after the completion of the D+3 cyclophosphamide infusion. Sirolimus will be continued at 5mg PO q24h starting on day +5. Trough levels will be maintained between 5-15 ng/mL.

Sirolimus or other immunosuppressant may be weaned and discontinued no earlier than 1 year post-transplant if donor myeloid and donor CD3 chimerism levels are >95%. Donor/recipient chimerism levels will be checked periodically during and after the taper. If lymphoid and/or myeloid donor chimerism levels decrease by >20%, sirolimus and/or other immunosuppressant may be restarted as clinically indicated. Total body irradiation TBI will be delivered in one fraction of 400 cGy on day -1. Equally weighted opposed lateral beams will be used to encompass the total body with the SCD patient positioned supine. Treatment will be delivered at an SAD of 6 meters (or other distance depending on the treatment room configuration). The prescription point will be the midplane at the maximal hip separation. The dose rate to midplane will be no more than 15 cGy per minute. Head and neck compensation will be used to increase dose homogeneity. Adjustments to treatment technique but not dose prescription may be made at the discretion of the treating radiation oncologist if deemed necessary. Gonadal shielding will be used in males unless refused.

Central Venous Line Placement

A double or triple lumen central venous catheter will be placed by a surgeon, interventional radiologist, or vascular access device specialist prior to transplantation.

Supportive Care

Blood Product Support

Filtered and irradiated blood products will be used in all SCD patients, regardless of CMV status. Platelet counts will be maintained at or higher than 50,000/ul (which is higher than usually maintained for non-sickle cell patient transplants) throughout the transplant to diminish the risk of intracranial bleeding. Peri-transplant target hemoglobin will be kept between 9-10g/dL or as designated by PI or AI as clinically indicated.

Infection Prophylaxis and Treatment

Penicillin VK 250 mg PO BID (or equivalent in penicillin-allergic patients) from day 0 until pneumococcal vaccination is complete post-transplant.

CMV monitoring and treatment:

Subjects will be monitored for CMV PCR in the blood at baseline and then at least weekly until day 100. CMV monitoring will be continued if possible for a minimum of 6 months post- transplant. Thereafter, monitoring will be performed as clinically indicated. CMV reactivation/disease will be treated according to TCT consortium guidelines.

EBV, HHV6, and adenovirus monitoring and treatment:

SCD subjects will be monitored for EBV, HHV6, and adenovirus PCR in the blood at baseline, at least weekly until discharge from the hospital, and then at least every 2 weeks until day 100. Thereafter, monitoring will be performed as clinically indicated. EBV reactivation/PTLD and adenovirus disease will be treated according to TCT consortium guidelines. HHV6 reactivation will not be treated unless compelling evidence exists for clinical disease related to HHV6. Such patients may receive foscarnet upon the advice of NIH infectious disease consultants.

Family members, including donors, will be offered influenza vaccination, as seasonally indicated. SCD patients will be offered influenza vaccination, as seasonally indicated, when they are at least 6 months post-transplant per CDC HSCT guidelines.

Prophylaxis and treatment of infections will otherwise be administered according to TCT consortium guidelines.

Anti-emetics

Anti-emetics will be administered according to NIH Pharmacy Department guidelines or with consultation of a clinical pharmacist.

Peripheral blood progenitor cell transplant (see Appendix C).

The target collection number for progenitor cells is ≥10 x 10^6 CD34+ cells/kg. This product will be collected in advance and cryopreserved. The volume processed per apheresis procedure will be determined by DTM medical staff on the day of apheresis, based on peak CD34 cell mobilization response to filgrastim (G-CSF) and the CD34 cell dose needed, based on kilogram weight of the recipient. This will range from 15 to 35 liters processed per day for 2 to 3 days, not to exceed a total of 75 liters over 3 days. In pediatric subjects, defined as less than 40 kg, a maximum of 8 blood volumes will be processed per day, for up to 1-3 days. A day 3 apheresis procedure will only be performed if the minimum dose of 5 million per kg is not met after the first two day collection. The minimum dose for proceeding to transplant will be ≥ 5 x 10^6 CD34 cells/kg. In order to meet the minimum dose, the donor may undergo a second mobilization a minimum of 2 weeks later. If after two such attempts, an inadequate cell number has been collected, the SCD patient and donor will be withdrawn from the protocol, unless another donor is available. Minor ABO incompatible grafts will have plasma removed per Transfusion Medicine protocol.

GVHD prophylaxis

Sirolimus will be started on day +4 with a loading dose of 5mg PO q4h for three doses, then 5mg PO q24h starting on day +5. Dosing may be modified by the PI or designee if clinically required due to reasons such as potential drug-drug interactions. Sirolimus will then be titrated to trough levels of 5-15ng/ml.

Subjects will be advised not to take medication with grapefruit juice and not to take St. John's wort while on sirolimus. Subjects must also be advised about limiting exposure to sunlight and UV light due to an increased risk of skin cancer. Women of childbearing potential will be informed of the potential risks during pregnancy and that they should use effective contraception prior to initiation of drug.

Nutrition

Parenteral nutrition will be instituted per the discretion of the clinical nutritionist, principal investigator or designee, and/or the inpatient team managing the care of the SCD patient.

Contraindication Listings

Filgrastim (G-CSF) will be listed as a contraindication for all patients with sickling disorders and

will only be given with the consent of the principal investigator, lead associate investigator, or the attending protocol investigator.

Bone Marrow Harvest

To date, we have performed bone marrow harvests on 6 patients with SCD, either because of 2nd transplant in the setting of busulfan or under our gene therapy protocol (14-H-0155). No complications other than hospitalization for pain have occurred to date. If required for autologous stem cell collection, bone marrow harvest will be performed with collection of sufficient nucleated cells prior to conditioning according to NIH standard operating procedure (target TNC 1 x 108/kg and maximum volume 20cc/kg). The patient will sign a separate consent at the time of the bone marrow harvest. Patients will be transfused with a target hemoglobin S of <30% prior to bone marrow harvest. They will also be admitted to the hospital at least overnight to monitor for complications related to the bone marrow harvest.

STUDY PARAMETERS

Primary Endpoint

The percentage of SCD patients at 100 days (+/- 1 week) post-transplant who have not rejected their grafts and who are without severe graft-versus-host disease (defined as grade 3 and higher acute GVHD and moderate to severe chronic GVHD). Therefore, as seen in the table, regimen failure is defined as graft rejection and/or history of severe GVHD at 100 days post-transplant.

	No graft rejection	Graft rejection
No GVHD	Desired outcome	Regimen failure
Yes GVHD	Regimen failure	Regimen failure

Secondary Endpoints

The level of chimerism required to maintain both graft survival as well as hematologic normalcy. The chimeric status of recipients will be measured on days +14 (or when subject starts to engraft), +30, +60 and +100, 6 months, 12 months, 18 months, 24 months, and annually thereafter by microsatellite analysis of the peripheral blood. More frequent monitoring may be required.

Incidence of donor type hemoglobin at 1 year post-transplant in SCD patients who have not been transfused in the previous 3 months.

Incidence of acute and chronic GVHD

Incidence of viral reactivation and disease

Disease-free survival and overall survival

Relapse rate and graft rejection rate

Transplant-related mortality

Determine whether specific haploidentical donors (i.e. parent versus sibling versus child) will decrease the incidence of regimen failure

Determine whether cyclophosphamide pharmacokinetics and/or germline allelic variants in drug metabolizing/transporting genes differ in SCD patients who do and do not experience graft rejection

Evaluate whether pentostatin pharmacokinetics differ in SCD patients who do and do not experience graft rejection

Evaluate the effects of transplant on reproductive and gonadal organ function

Evaluate the effects of transplant on patient quality of life

These secondary endpoints may be achieved by monitoring the following parameters:

$CD34^+$ cell dose, $CD3^+$ cell dose

Degrees of donor-recipient lymphoid, myeloid, and erythroid chimerism by microsatellite PCR analysis and normal hemoglobin quantitation- either by gel electrophoresis or if necessary by flow analysis – using peripheral blood as appropriate.

Neutrophil recovery (defined as the first of 3 consecutive absolute neutrophil counts>500/uL).

Platelet recovery (defined as 7 days after the last platelet transfusion or the first of 3 consecutive labs showing a platelet count of >50,000 after nadir, whichever is later)

Red cell recovery (defined as 7 days after the last red blood cell transfusion)

Non-hematologic effects attributable to the preparative regimen

Transplant-related mortality by 1 year

Hemoglobin F and S levels on hemoglobin electrophoresis

Development of further neurologic disease

Gonadal organ function as reflected by hormone levels, normal menstrual cycle, and a voluntary reproductive health questionnaire (see appendix E).

Quality of life

Neuropsychologic testing

Assessment of immune reconstitution

Assessment of lymphocyte function

Assessment of biomarkers associated with graft rejection and tolerance induction

RESEARCH STUDIES

Up to 50 mL of donor (adult or pediatric) blood may be collected prior to cell donation (and prior to GCSF mobilization) for research.

The amount of blood that may be drawn from adult recipients for research purposes shall not exceed 10.5 mL/kg or 550 mL, whichever is smaller, over any eight week period.

For pediatric recipients, no more than 5 mL/kg may be drawn for research purposes in a single day, and no more than 9.5 mL/kg may be drawn over any eight-week period.

Once a standard leukapheresis product from a donor has been found to meet target cell dose, 1 mL of additional cells from the collection can be used for research under this protocol.

Chimerism Studies

We will use PCR analysis of microsatellites to identify the contribution of the donor marrow to post-transplant hematopoiesis and to detect donor lymphocytes in the circulation. Approximately 10 mL will be drawn around days 14 (or when subject starts to engraft), 30, 60, 100, and every 6-12 months thereafter (only if patient remains engrafted).

Bone Marrow Samples

A volume (up to 25 ml) of bone marrow aspirate will be collected for research studies at the pre-transplant evaluation, day 100 post-transplant (+/-2weeks) and/or when full donor erythroid chimerism is attained, 12 to 18 months post-transplant, or as clinically indicated. These will be used to help elucidate the contribution of the progenitor cells to the circulating component, explore mechanisms of graft rejection and tolerance, and evaluate bone marrow morphology and cytogenetics. Bone marrow core sample to be done at baseline and then only as clinically indicated.

Hematopoietic Stem Cells

Following collection of autologous "back-up" stem cells, at least 2 X 106/kg CD34 cells or 1 x 10^8/kg TNC will be cryopreserved and stored. The rest will be aliquoted and stored for gene therapy research involving genetic correction of DNA to produce normal hemoglobin. CD34- cells will be frozen for research testing as described in sections 8.11 and 8.12. Additional blood samples may be drawn before plerixafor administration and 2 hours after plerixafor to study cytokine levels, CD3, CD34, and total nucleated cell counts.

Transthoracic Echocardiography

Transthoracic echocardiography will be performed to assess TR jet velocity and markers of systolic and diastolic function at study onset, Day 100, 6 months, 18 months and yearly post transplantation, or more frequent as clinically indicated.

Cardiac MRI and myocardial fibrosis biomarkers

Cardiac MRI will be performed at baseline and 2 years year post-transplant to assess factors such as cardiac structure, cardiac volumes, left ventricular ejection fraction, iron deposition, myocardial fibrosis, and extracellular volume fraction with gadolinium enhanced methods where indicated. We will also measure circulating myocardial fibrosis biomarkers at baseline and 2 years, including C-terminal propeptide of procollagen type I, N-terminal propeptide of procollagen type III, Matrix metalloproteinase-9, Tissue inhibitor of metalloproteinases-1, microRNA-29b, Transforming growth Factor-β1, Galectin-3, Midregional pro-atrial natriuretic peptide, and IL-18. We will evaluate the effect of transplant on those factors. These blood samples will be derived from the same 44 mL blood samples described below in section 8.12.The cardiac MRI may also be completed under the NHLBI 000479 protocol for those patients enrolled on both protocols.

Dual X-ray Absorptiometry (DEXA Scan)

Through bone density scans recommended by routine endocrinology consults in 20 subjects with sickle cell disease, we discovered that more than half of our patients were osteopenic or osteoporotic before transplant. Therefore, to formally study the bone effects from sickle cell disease and to monitor the effect of transplant on bone disease, we would like to perform DEXA scans in all patients pre-transplant and every 1-2 years post-transplant depending on how severe their bone disease is.

Right Heart Catheterization

SCD patients who are diagnosed with pulmonary hypertension prior to transplant may undergo repeat right heart catheterization at least 1 year post-transplant to document whether the pulmonary hypertension has improved or resolved. Right heart catheterization will be performed in the NHLBI interventional catheterization suite under X- ray and/or MRI guidance, or right-heart catheterization via balloon flotation or X-ray in the NIH critical care or intermediate care units. The procedure will be performed by credentialed cardiologists or critical care physicians.

Assessment of Quality of Life

PROMIS (patient reported outcome instrument) is freely available and has been applied in many diseases and conditions, and will be a useful tool to assess the overall physical and mental health perception in our

SCD patients before and after BMT. The PROMIS 57 assesses various aspects of quality of life, including physical function, fatigue, social functioning, anxiety, and depression. It takes about 10 minutes to complete. The questionnaire will be administered either electronically or with pencil and paper. For patients that co-enroll onto the 000697 protocol the questionnaires will be completed electronically into the 000697 REDCap system. For patients that do not enroll onto the 000697 protocol, the questionnaires may be completed with pencil and paper. PROMIS questionnaires will be completed pre-BMT, day 100, one year, and 2 years post BMT. If possible, the questionnaire will also be completed on day 30, day 60, 6 months, three years, four years, and five years post BMT. After 5 years the PROMIS questionnaire remains optional and may be completed at a patient's annual visit. Compensation for patient's time will be provided if the questionnaires are completed.

Neuropsychologic Testing

Neuropsychologic testing, performed by our licensed psychologists or other members of the neuropsychology group (supervised by a licensed psychologist), will include but not be limited to the Wechsler Abbreviated Scale of Intelligence and several subtests of the Wechsler Adult Intelligence Scale – Fourth Edition at baseline, 1 year, and 2 years (+/- 6 months) post BMT. The comprehensive evaluation will take about 60-90 minutes to complete. In addition, a brief monitoring battery assessing selective domains such as attention, executive function, and processing speed will be administered at 100 days (+/- 7 days) post BMT. The monitoring battery will take about 45 minutes to complete. SCD patients will not be tested when they are febrile, and will be given breaks as needed.

Immune Reconstitution

Lymphocyte subpopulations and immunoglobulin levels (IgG, IgA, and IgM) will be quantified before transplant, and approximately 30 days, 60 days, 100 days, 6, 12, and 18 months post-transplant, and yearly thereafter until results have normalized. Approximately 7 mL of blood will be collected at each time point. Lymphocyte subpopulations and other immune cells may also be characterized by flow cytometry for specific markers associated with developmental stage, function and alloreactivity which would require an additional 1-2 blood samples (approx. 30 mL each). When feasible, samples may also be drawn at days +150 and +270 post-transplant.

Assessment of Cytokines and Lymphocyte Function

Donor serum and lymphocytes will be collected pre-transplant. These blood samples will be derived from the same 40ml of blood drawn from the samples described below in section 8.12. SCD patient serum and lymphocytes will be collected pre-transplant and at 6 and 12 months post-transplant. These blood samples will be derived from the same 44 mL blood samples described below in section 8.12. Engrafted patients who discontinue immunosuppression will have additional samples (30ml in EDTA tubes) collected just prior to stopping immunosuppression and 6 months after discontinuing immunosuppression (+/- 1month). Finally, in patients that develop graft rejection or GVHD, recipient serum and lymphocytes (30ml in EDTA tubes) will be collected once within 2 weeks of the onset of clinical symptoms, once just prior to discontinuing immunosuppression or at 12 months post-transplant, whichever is longer, and once 6 months after immunosuppression is discontinued or at 18 months post-transplant, whichever is longer. Serum tumor necrosis factor, interferon-gamma, and interleukin-17 will be quantified. Levels may continue to be followed every 6 to 12 months thereafter based on the results during the first 18 months.

Donor/host alloreactivity will be assessed at each time point by CFSE dilution assay in the presence and absence of sirolimus in vitro. We will also measure mTOR signaling in recipient T cell samples by flow cytometry for phospho p70 S6 Kinase and phospho AKT in response to anti CD3 plus anti CD28 stimulation in the presence or absence of sirolimus in vitro. Recipient effector T cell subsets will be measured at each time point by intracellular cytokine staining for interferon gamma, interleukin-4, interleukin-17, and FoxP3. Regulatory T cell subsets (approximately 44 mL) will also

be collected serially at baseline and at 1 week, 2 weeks, 3 weeks, 1 month, 6 weeks, 2 months, 100 days, 6 months, 12 months, 18 months, 24 months, and yearly thereafter post-transplant to help inform whether patients may be tolerant of their donor grafts to inform a potential future study where if patients appear to be tolerant based on increased regulatory T cell expression and decreased donor-specific reactivity, sirolimus can be systematically weaned so that patients will not unnecessarily receive immunosuppressive drugs.

CMV pp65 specific T cell responses will be measured in cases where either donor or recipient is CMV positive. These will be assessed by ELISPOT for interferon-gamma in response to pooled overlapping pp65 peptides.

Evaluation for Markers of Graft Rejection

Blood samples (using the same 44 mL blood samples described in section 8.11) will be collected serially at baseline and at 1 week, 2 weeks, 3 weeks, 1 month, 6 weeks, 2 months, 100 days, 6 months, 12 months, 18 months, 24 months, and yearly thereafter post-transplant. Forty mL of blood will also be drawn from each donor at baseline. CRP, ESR, cytokine and immunophenotyping studies will be performed to evaluate for biomarkers associated with primary graft failure, acute rejection, and chronic rejection. Other assays may be performed based on our initial findings and as new methods are introduced. Samples may be collected more frequently as necessary based on our preliminary results. Donor-derived cell-free DNA levels (10 mL blood) will also be measured to evaluate whether acute elevation predicts subsequent graft rejection[94].

Pharmacokinetic Studies

Pharmacokinetics of Pentostatin

Blood samples (approx. 5 mL each time) will be drawn on day -21 at the beginning of and at 5 and 10 minutes and 2, 6, 8, and 24 hours after the end of the pentostatin infusion to determine plasma pentostatin concentrations[95]. These data will be used to create plasma pentostatin versus time profiles.

Pharmacokinetics of Campath

Blood samples (3mL) will be drawn at days 7, 14, 21, and 28 post-transplant (+/-7days).

Pharmacogenomic Studies

Peripheral blood (10mL) will be drawn from the recipient prior to transplant to perform genotyping for single nucleotide polymorphisms in CYP3A4/5, CYP2B6, CYP2C9, and C3435T. If the original sample has insufficient DNA for analysis, then an additional sample will be obtained from SCD patients prior to transplant.

Plasma lipid analysis including VLDL, LDL and HDL particle size and number will be measured by nuclear magnetic resonance (NMR) spectroscopy using the Vantera Clinical Analyzer (LabCorp., Burlington, North Carolina) in the NIH Clinical Center. VLDL-P, LDL-P and HDL-P were quantified based on lipoprotein particle size using the amplitudes of their distinct lipid methyl group NMR signals to calculate the different lipoproteins. Lipoprotein particles were further subdivided into large, medium, and small particles based on the mean particle sizes as the weighted average of related subclasses. Apolipoprotein B will also be measured.

HLA Antibodies and C1q Testing

HLA antibodies: blood samples (5ml) will be collected from the recipient at pre-transplant (baseline) and days 30, 60, and 100 (+/-7 days). Then again at 6 months and 1 year post-transplant (+/- 3 months). In patients with DSAs (donor specific antibodies), additional HLA antibody samples will be collected weekly post-transplant (+/- 7 days) until day 30.

Blood samples (10mL) will be collected from recipients with DSAs to check C1q levels[96] pre-transplant

and post-transplant weekly until day 30, then at day 60 and day 100 (+/- 7 days), and at 6 months and 1 year (+/- 3 months). Currently, it is unclear whether C1q levels are associated with graft rejection in patients with MFI <2000. Therefore, the samples will be batched and therefore won't be used in making decisions regarding whether to move forward with transplant.

Functional Fluidics and Dr. William Eaton Collaboration to Study Red Blood Cell Biology

In addition to the red blood cell biology studies described above through our collaboration with Functional Fluidics, blood may be drawn once from donors with their research blood draw and from recipients twice at baseline and at 3 (Day 100), 6, and 12 months post-transplant as listed below to evaluate the time for red blood cells to sickle in a deoxygenated environment.

Five peripheral blood samples, 3ml each for Functional Fluidics and 1mL each to Dr. Eaton may be drawn from the recipient at the following timepoints:

Baseline (2 samples at baseline; each sample done 2 consecutive weeks after hydroxyurea is stopped prior to exchange transfusion and plerixafor autologous collection).

One sample at 3 months (Day 100) post-transplant

One sample at 6 months post-transplant.

One sample at 12 months post-transplant.

In addition, one sample may be drawn from the donor with the research blood draw.

These samples will be collected from approximately 5 patients starting in October 2020; more patients may be added depending on the initial findings and our funding opportunity. Further, the collaboration with Dr. Eaton may be ongoing.

Brain MRI/MRA with cerebral blood flow and oxygen extraction fraction

Our collaborators sought to develop more rigorous and comprehensive brain MRI tools to evaluate the spectrum of hemo-metabolic changes in SCD by evaluating quantitative measures of cerebral blood flow (CBF, rate of blood delivery to tissue, mL/100g/min), oxygen extraction fraction (OEF, ratio of oxygen consumed to oxygen delivered), the cerebral metabolic rate of oxygen consumption (CMRO2), and their relevance for signaling infarct development and response to conservative and aggressive therapies. Angiography only identifies large vessel vasculopathy, but it is plausible that smaller vessels including arterioles and capillaries develop stenotic disease, which would lead to an increase in OEF if local CBF cannot increase to compensate for anemia. Patients with SCD commonly have increased CBF[97-102] in a manner that depends on several variables, including the balance of HbS fraction and oxygen carrying capacity[103], extent of vasculopathy[104], and autoregulatory reserve capacity (e.g.: how close microvasculature is to maximal blood volume and flow from vasodilation[105, 106]. CBF and OEF have been proposed to be indicators of cerebral ischemia risk[106, 107]. OEF co-localizes with infarcts in children[108], and has been considered as a promising metric in both children and adults with SCD[107, 109]. Our collaborators have reported that in contrast to two healthy volunteers, CBF and OEF were elevated at baseline and both parameters significantly improved approximately one year after successful haploidentical HSCT for SCD[110]. The addition of OEF and CBF testing will add only 8 to 10 minutes to the MRI scan time. No data will be sent to our collaborators until a Data Transfer Agreement is executed. OEF and CBF testing will be optional; if those research tests are not added, a standard brain MRI will be performed. The brain MRI will be completed at baseline, 1 year and 2 years post-transplant. The baseline and 2 year post-transplant MRIs may be completed under the 000479 protocol for patients enrolled on both protocols.

8.19 Fertility Surveys

Fertility questionnaires will be provided to patients at baseline, 1 year and 2 years post-transplant.

The fertility surveys are titled: Fertility Survey (female), Changes in Sexual Function Questionnaire (male/female), Priapism survey (male) and the International Index of Erectile dysfunction (male) (see

appendix I). After two years, the surveys are optional at annual visits. For patients that co-enroll onto the 000697 protocol, the questionnaires will be completed electronically into the 000697 REDCap system. For patients that do not enroll onto the 000697 protocol, the questionnaires may be completed with pencil and paper. Compensation for the patient's time will be provided if the questionnaires are completed.

HUMAN SPECIMEN USE, DISPOSITION, TRACKING AND STORAGE OF SAMPLES AND DATA

During the course of participating on this study, blood, tissue and data will be collected for correlative laboratory research studies. Specimens collected strictly for research purposes will not be read by a pathologist.

Biospecimen management: Specimens and their derivatives (e.g., genomic material, cell lines) will be coded and stored in conformity with DIR Policy (e.g., BSI). Coded biospecimens may be sent to collaborators outside of the NIH in accordance with applicable NIH and DIR Policies for sharing research resources, including an executed material transfer agreement. Biospecimens with subject personal identifiers may be sent to associate investigators and collaborators outside of the NIH only after appropriate institutional approvals such as: an executed reliance agreement with the NIH Intramural IRB, or an extension of the NIH FWA through an Individual Investigator Agreement.

Data Management: The principal investigator, associate investigators, research nurses and/or a data manager will assist with the data management efforts. Data will be abstracted from Clinical Center progress notes as well as intake forms and the case report forms. Laboratory data from NIH will be reviewed using CRIS. The principal investigator, associate investigators/research nurses and/or a contracted data manager will assist with the data management efforts to ensure that data are verifiable and evaluable. Data will be abstracted from Clinical Center progress notes as well as from progress notes forwarded from the subjects' home physician.

The PI will be responsible for overseeing entry of data into an in-house password protected electronic system and ensuring data accuracy, consistency and timeliness. Laboratory values from referring home physicians will be entered into the system. The principal investigator, associate investigators/research nurses and/or a contracted data manager will assist with the data management efforts to ensure that data are verifiable and evaluable.

Research data will be prospectively collected by authorized Investigator personnel and entered into an NHLBI, 21 CFR 11 compliant, database which will consist of the study specific set of electronic CRFs (e-CRFs) used for capturing, managing and reporting clinical research data.

The database will maintain complete data records on each research subject. Subjective and objective patient experiences during the duration of the study will be documented in the patient medical record notes. These protocol notes will serve as the primary source material from which data will be collected in the database. Any pertinent supplementary information obtained from outside laboratories, outside hospitals, radiology reports, laboratory reports, or other patient records will be used as additional sources for data collection.

We will maintain the confidentiality of identifiable private information collected in this Clinical Trial and protect the privacy of the individual human subjects. Primary data containing individually identifiable information obtained during the conduct of the protocol will be kept in secure network drives or in approved alternative sites that comply with NIH information security standards. Neither individual personal identifiers nor the key linking coded data to individuals will be released without prior IRB approval and an executed confidential disclosure agreement (CDA) or data transfer agreement (DTA). Identifiable data will not be sent outside NIH without prior IRB approval or appropriate conditions for disclosure outlined in the executed CDA or DTA.

Storage: All samples will be stored in the laboratory of Dr. Fitzhugh. Collected samples will be de-identified prior to storage in the laboratory of the principal investigator following current NHLBI DIR BSI Policy.

Efforts to ensure protection of patient information include;

Each sample is assigned a unique number.
Vials holding patient samples are labeled with the sequential laboratory accession ID number that does not contain any personal identifier information.
An electronic database is used to store patient information related to the coded samples
The laboratory is located in a controlled access building and laboratory doors are kept locked. Visitors to the laboratory are required to be accompanied by laboratory staff at all times.

Hard copy records or electronic copies of documents containing patient information are kept in the locked laboratory or other controlled access locations.

Christopher Gamper, MD, and Jonathan Powell, MD, affiliated with the Johns Hopkins University, will receive de-identified patient samples to assess cytokine and lymphocyte function as described in section 8.11 and William D. Figg, Sr. PharmD, MBA, CRC/NCI/OCD will receive recipient samples to assess cyclophosphamide and pentostatin pharmacokinetic studies as described in section 8.13 and cyclophosphamide pharmacogenomics studies as described in section 8.13. As of May 26, 2023, we have analyzed cyclophosphamide pharmacokinetics and found no differences in clearance, nor any data to suggest cyclophosphamide clearance impacts graft rejection rates; we will therefore stop measuring cyclophosphamide pKs. All other research samples will be stored in the principal investigator's laboratory. Samples will never be labeled with the patient's name. Samples will be assigned a unique code known only to the principal and associate investigators, which will serve as a link to the patient's clinical information collected as part of this research protocol. Therefore confidentiality is protected. Samples will only be sent to collaborators after execution of an MTA.

Upon execution of an MTA, de-identified peripheral blood, peripheral blood stem cell contents obtained from the discarded bag, and clinical data may be sent to Dr. Allistair Abraham at Children's National Medical Center to compare donor/recipient lymphocyte contribution and NK cell phenotypes, to Dr. Elizabeth Stenger at Emory University to evaluate additional cytokines that may be associated with graft rejection, and to Dr. Patrick Hines at Functional Fluidics to evaluate the effect of transplant on blood flow adhesion to a substrate (i.e. vascular cell adhesion protein-1 (VCAM-1) or P selectin) and red blood cell mechanical fragility.

Data sharing and future use of data

Research data may be shared with qualified non-collaborator recipients following publication of the primary research results after removal of PII. Refusal of a research subject participant to permit future use of data--other than required in the protocol or by the FDA--will be honored. Limitations in data sharing and future use of data due to contractual obligations (e.g., CRADAs) or intellectual property proceedings (such as patent filings) will be honored.

Future use of biospecimens

Following analyses of biospecimens for primary research purposes, remaining samples suitable for future research will be stored in the Principal Investigator's laboratory freezers in a coded manner to ensure data protection.

Any future research use of biospecimens not defined in the protocol in which NHLBI investigators are engaged in research (e.g., they are undertaking research activities and hold the key that identifies research subjects) requires IRB review and approval. Coded biospecimens (NHLBI investigators hold the key that identifies research subjects) to be shared outside of NIH for future research use where results will not be returned to the Principal Investigator does not require IRB review or approval. ***Tracking:*** Samples will be ordered and tracked through CRIS Research Screens. Should a CRIS screen not be available, the NIH form 2803-1 will be completed and will accompany the specimen and be filed in the medical record.

***Tracking**:* Samples will be ordered and tracked through CRIS Research Screens. Should a CRIS screen not be available, the NIH form 2803-1 will be completed and will accompany the specimen and be filed in the medical record.

***End of study procedures**:* Samples from consenting subjects will be stored until they are no longer of scientific value. At the completion of the protocol (termination), samples and data will be maintained in a repository for future research.

Loss or destruction of samples: Should we become aware that a major breech in our plan for tracking and storage of samples has occurred, the IRB will be notified.

Loss or destruction of data: Should we become aware that a major breech in the plan to protect patient confidentiality and trail data has occurred, the clinical director and IRB will be notified.

Privacy and Confidentiality: All efforts, within reason, will be made to keep subjects' and private identifiable information (PII) private. Using or sharing ("disclosure") such data must follow federal privacy rules. Under certain circumstances, the United States Office of Human Research Protections (OHRP), The US Food and Drug Administration (FDA), and the NIH Intramural Institutional Review Board (IRB), will be able to inspect and copy confidential study-related records which identify participants by name. Therefore, absolute confidentiality cannot be guaranteed.

CLINICAL EVALUATION AND PLAN – SCD PATIENT

Study visits may be conducted electronically per Principal Investigator's (PI) discretion . In these scenarios, only NIH approved electronic technology and telehealth platforms will be used to communicate with subjects and will allow the PI and approved Associate Investigators (AIs) to obtain medical history, perform a limited physical exam, and assess for adverse events.

Pre-study evaluations test results (screening/baseline) may be used from any IRB approved NHLBI protocol within the specified timelines below:

Screening evaluations: performed within 3 months of signing consent for this treatment protocol.

History, physical examination, height and weight
ECOG performance status
High resolution molecular HLA- A, -B, -Cw,- DRB1, and -DQB1 typing of patient and as many family members as possible and/or necessary to confirm haploidentical matching of the donor (HLA testing is usually completed in advance on another IRB approved protocol and could be months or years before the patient consents to this protocol. The viability of the samples are per the HLA Lab SOP.)
Hemoglobin electrophoresis and/or flow cytometric analysis of hemoglobin A, F and SS, SC, S-β–thal0, or other sickle genotypes as appropriate (for both patient and donor).
PT, PTT, d-dimer, CBC with differential, reticulocyte count, type & antibody screen
Acute care panel, hepatic panel, mineral panel, creatinine kinase, uric acid, LDH, cystatin C, total protein
HLA antibody screen (Initial screen usually done in advance on another IRB approved protocol). This test is sometimes repeated again closer to transplant.
Chest radiograph
Pulmonary function test & 6 minute walk test
Brain MRI/MRA
Transthoracic ECHO (pulmonary hypertension screening by tricuspid regurgitant velocity (TRV) analysis using standard transthoracic echocardiography)
Antibody screen for HBV, HCV, HIV, EBV, CMV

Baseline Evaluations: performed within 3 months of signing consent for this treatment protocol unless specified below

All evaluations will be done under NIH blood draw limitations and in accordance with NIH guidelines.
Pre-study evaluations test results (baseline) may be used from any IRB approved NHLBI protocol within the specified timelines below:

Consider PPD/Quantiferon gold test for patients from areas where tuberculosis is prevalent and malaria screening for patients from areas where malaria is endemic.
Antibody screen for HIV-1/HCV/HBVNAT, HTLV-I/II, Anti-adenovirus antibody, adenovirus PCR, Anti-varicella IgG. West Nile, T. Cruzi, HSV Type 1/2, HAV, toxoplasma, syphilis, Babesia testing.
Iron & transferrin
Prealbumin, osmolality serum, CRP, ESR
Folate, Vitamin B12, Pro Brain Naturetic Peptide, Lipid panel, apolipoprotein profile, lipoprotein profile
Extended red cell phenotyping
Haptoglobin, direct antiglobulin screen, isohemagglutinin titer
STR profile
KIR genotyping (may be done > 3 months prior to consent signing)
Sinus, chest, abdomen and pelvis CT scans as clinically indicated
Cardiac function: EKG, transthoracic ECHO (if not already completed), 24 hour holter, Troponin T or Troponin I
Cardiac MRI and myocardial fibrosis biomarkers
Abdominal Ultrasound
Lymphocyte subpopulation analysis and serum immunoglobulin levels
Baseline research samples (blood and bone marrow) as outlined in section 8.
Nutritional assessment
24 Hour urine collection for assessment of creatinine clearance, creatinine, protein, albumin, phosphate, uric acid, urine protein electrophoresis
Random urine sample for phosphate, uric acid, osmolality, protein/ creatinine ratio and albumin/creatinine ratio
Dental review
Social worker interview
Ophthalmology consultation
Interview with members of primary care team and visit to unit
Quality of life assessment
Neuropsychologic testing
Endocrine consultation (if clinically indicated)
Endocrine lab testing which will include: DEXA Scan, thyroid panel (TSH, T3,T4), insulin-like growth factor 1, morning cortisol, ACTH stimulation test, fasting glucose, fasting insulin, oral glucose tolerance test, serum fructosamine level, 25-hydroxy vitamin D, growth hormone, progesterone and in male patients, testosterone, luteinizing hormone level (LH), and follicle stimulating hormone (FSH), and in female patients, LH, FSH, anti-mullerian hormone, prolactin and estradiol, growth hormone, adrenocorticotropic hormone (ACTH).
Complete fasting: lipid profile with triglycerides, lipoprotein profile and apolipoprotein B
Abdominal MRI T2* (Dr. Gharib's group)
Gynecology consult (females)
Hepatology & Pulmonology consults (if clinically indicated)
Reproductive health questionnaire (see appendix E).
β-HCG serum pregnancy test for females of childbearing potential will be checked within 1 week of the patient signing consent.
C1q test in patients with DSAs
Transfusion medicine consult and Infectious disease consult
Possible Functional Fluidics sample & Dr. Eaton Sample

Fertility questionnaires as described in section 8.19

Inpatient Monitoring

All patients with a sickling disorder undergoing transplant will be listed as having a contraindication to the use of filgrastim (G-CSF). Labs will be drawn at the following timepoints or as clinically indicated.

Starting D-21 conditioning:

CMV/EBV PCR weekly.

While inpatient:

Once daily: CBC with differential, acute care panel, mineral panel, hepatic panel, LDH, Total protein, uric acid, creatinine kinase

Twice weekly: CMV, EBV, reticulocytes, pre-albumin, sirolimus level, cystatin C and PT/PTT

Weekly: Adenovirus, and HHV6 surveillance

Every two weeks: serum cholesterol, triglycerides

Follow-up to Day 100: Outpatient

At least every 2-3 weeks up to 100 days and when clinically indicated: CBC, reticulocyte count, hemoglobin electrophoresis to assess HbS and HbF levels, coagulation screen, acute care panel, hepatic panel, mineral panel, creatinine kinase, lactate dehydrogenase, total protein, uric acid, sirolimus level (for engrafted patients), EBV, adenovirus, HHV6 surveillance. A complete physical exam will be repeated as clinically indicated.

Weekly until day 30 (+/- 7 days): HLA antibody and C1q testing in patients with DSAs.

Day 30, day 60, and day 100 post-transplant +/- 7 days: HLA antibody testing samples. C1q levels will also be collected in patients with DSAs.

Weekly until day 100 (+/- 7 days): CMV/EBV PCR

Day 100 (+/-7 days) and if possible day 30 and day 60 (+/- 1 month): Quality of life assessments will be administered.
Day 100 (+/- 7 days): Neuropsychologic testing and ECOG score.

Day 100 (+/-2 weeks): bone marrow aspiration (core biopsy if clinically required)

At 1 week, 2 weeks, 3 weeks, 1 month, 6 weeks, 2 months, and 100 days post BMT (+/- 7 days): Graft rejection markers, Cytokine, cell-free DNA, and immunophenotyping studies will be drawn as per section 8.12.

At 2 weeks, 1 month, 2 months, and 100 days (+/- 7 days): lipoprotein profile, apolipoprotein, lipid panel

Monthly (+/- 7 days): lipid panel (serum cholesterol, triglycerides.)

Peripheral blood will be drawn on days +14 (or when subject starts to engraft), +30, +60 and +100 (+/- 7 days) to assess for donor-host chimerism in the lymphoid, myeloid, and erythroid cell lines including the use of HbS and HbA levels (for engrafted patients). Chimerism levels are collected when subjects have engrafted.

100 Days post BMT (+/- 7 days): ECHO, Troponin T or Troponin I, d-dimer, Pro-BNP, quantitative immunoglobulins, TBNKDay 100 (+/- 7 days): Functional Fluidics and Dr. Eaton samples (if applicable i.e. sample max not reached).

At 30 days and 100 days post BMT (+/- 7 days): Isohemagglutinin titer (not indicated if donor and recipient

have the same ABO blood type)

Beyond Day 100

At <u>**6 months and 1 year**</u> (+/- 3 months) post-transplant: HLA antibody testing (all recipients), C1q testing for DSA positive participants, Functional Fluidics, and Dr. Eaton samples (if applicable i.e. sample max not reached).

<u>At 6, 12, 18, 24, 36, 48 and 60 months (+/- 3 months):</u> CBC, reticulocyte count, d-dimer, HbS, HbA, and HbF levels, acute care, hepatic panel, creatine kinase, mineral panel, lactate dehydrogenase, total protein, uric acid, cystatin C, serum cholesterol, triglycerides, lipoprotein profile, apolipoprotein B, chimerism studies (for engrafted patients), Pro-BNP, transthoracic echocardiogram, 6 minute walk, and pulmonary function testing.

<u>At 6, 12, and 18 months (+/- 3 months):</u> blood will be drawn into heparinized tubes for preparation of plasma and lymphocytes for in vitro studies, and serum immunoglobulin levels and lymphocyte subpopulation analysis will be performed.

<u>At 6 months, 12 months, 18 months, 24 months, and yearly thereafter (+/- 3 months):</u> Blood will be drawn for graft rejection markers to perform cytokine, cell-free DNA, and immunophenotyping studies.

<u>At 6 months and 1 year post BMT (+/- 3 months):</u> Isohemagglutinin titer (not indicated if donor and recipient have the same ABO blood type)

Bone marrow aspirate samples will be obtained at <u>day 100 post-transplant</u> (+/- 2 weeks) and/or when full donor erythroid chimerism is attained or if clinically indicated and at <u>12 to 18 months post-transplant</u> (+/- 3 months).

Patients will be screened for CMV beyond day 100-post transplant for a minimum of 6 months.

Quality of life assessments will be administered at 1 year and 2 years (+/- 6 months) along with neuropsychological testing, and if possible at 6 months, 3 years, 4 years, and 5 years post BMT (+/- 3 months) and then it may be done annually beyond 5 years.

Neuropsychologic testing will be performed and a reproductive health questionnaire distributed (see appendix E) at 1 and 2 years post BMT (+/- 6 months).

Additional fertility surveys may be done annually.

ECOG score: 6 months, 1 year, 2 years, 3 years, 4 years, 5 years.

Cardiac MRI and fibrosis biomarker analysis will be performed at 2 years (+/- 3 months) post BMT and brain MRI/MRA will be performed at 1 and 2 years post- BMT (+/- 3 months).

<u>At 12, 24, 36, 48, and 60 months (+/- 3 months):</u> thyroid stimulating hormone, free thyroxine, insulin-like growth factor 1, morning cortisol level, fasting glucose, fasting insulin, hemoglobin A1C and serum fructosamine, 25 hydroxy-vitamin D, progesterone, growth hormone, ACTH and in male patients, testosterone level, luteinizing hormone level (LH), and follicle stimulating hormone level (FSH), and in female patients, LH, FSH, anti- mullerian hormone level, and estradiol level.

DEXA Scans will be performed every 1 to 2 years depending on the severity of the bone disease.

If a patient requires a second stem cell transplant on a different protocol before completing the 5 year follow up visit on this protocol the follow up schedule will then convert to the protocol timelines of the most recent transplant protocol.

After 5 years post-transplant, the above follow-up visits and evaluations are not mandatory,
but are encouraged. At minimum, yearly communication with the patient and the referring physician will be

attempted in order to monitor for any grade 3 adverse events that may be related to the transplant procedure. A long-term health effects protocol is in progress and once this protocol is open, all patients beyond 3 years post-transplant will be offered to transition to the long-term health effects protocol.

CLINICAL EVALUATION AND PLAN – DONOR

Study visits may be conducted electronically per Principal Investigator's (PI) discretion for existing subjects already enrolled onto the protocol. In these scenarios, only NIH approved electronic technology and telehealth platforms will be used to communicate with subjects and will allow the PI and approved Associate Investigators (Ais) to obtain medical history, perform a limited physical exam, and assess for adverse events.

Pre-study evaluations may be performed under any IRB approved NHLBI protocol. Evaluation of related donor suitability and eligibility will be done in accordance with existing Transplant and Cellular Therapy Consortium supportive care guidelines HPC procurement document. Some additional specific evaluations are below.

Pre-Study Consult and Evaluation

HLA typing of as many family members as necessary

Confirm HLA haploidentity of donor with patient

History and physical examination with provider from another transplant team.

Hemoglobin electrophoresis

Hepatitis B, C, HIV, HTLV-I/II, CMV/EBV antibodies, RPR

CBC with differential, coagulation screen, comprehensive metabolic panel

Extended red cell phenotyping, HLA Ab screening

KIR genotyping

Chest x-ray (if medically indicated)

EKG

Fit to donate: Orientation- visit to Department of Transfusion Medicine for inspection of veins to determine the need for a central line for apheresis

Consent to undergo filgrastim (G-CSF) mobilization (see below)

Donors will have a follow-up visit within 2 weeks of starting G-CSF

Donors who consent will have baseline research samples collected as outlined in section 8.0.

Transfusion Medicine Consult

No protocol related tests are required to determine donor eligibility to donate research blood samples.

MANAGEMENT OF SCD PATIENT COMPLICATIONS

The major complications are viral reactivation, acute and chronic GVHD, and relapse of the original disease. Patients with these complications will be treated along the following lines:

12.1 Viral Reactivation

Patients will be treated according to TCT consortium guidelines (see section 6.7.2)

12.2 Acute GVHD

Sirolimus may be continued and other immunosuppressive drugs may be used as clinically appropriate.

12.3 Chronic GVHD

Sirolimus may be continued and other immunosuppressive drugs may be used as clinically appropriate.

12.4 Graft Rejection

Patients with graft rejection may be treated with standard of care treatment options with or without stem cell rescue (a 2nd transplant, using a conventional conditioning regimen) or referred back to their primary physician depending on what is considered to be in the best interest of the patient. Patients who receive a second transplant under another protocol can be followed on this protocol long-term to monitor for late effects post-transplant. This transplant protocol uses a nonmyeloablative preparative regimen. Therefore, autologous recovery (hence relapse of disease) is anticipated in patients who fail to engraft (see 6.3). To avoid the rare situations where patients fail to engraft and recover autologous hematopoiesis, autologous HSCs will be collected from patients for rescue.

12.5 Relapse of Disease

Patients with disease relapse may be treated with standard of care treatment options with or without stem cell rescue (2nd transplant, using conventional conditioning regimen) or referred back to their primary physician depending on what is considered to be in the best interest of the patient.

12.6 Use of Immunosuppressive Drugs

In the event that a subject has an adverse reaction to sirolimus, alternative immunosuppressive drugs may be used as clinically appropriate.

DATA AND SAFETY MONITORING

13.1 Safety Monitoring

Principal Investigator: The safety of interventions and treatments associated with this protocol will be under continuous review by the investigative team. Accrual, efficacy, and safety data will be monitored by the PI.

NIH Intramural IRB: Prior to implementation of this study, the protocol and the proposed patient consent forms will be reviewed and approved by the properly constituted IRB operating according to the 45 CFR 46. This committee will also approve all amendments to the protocol or informed consent, and conduct continuing annual review so long as the protocol is open to accrual or follow up of subjects.

NHLBI DSMB: The NHLBI Data Safety and Monitoring Board will review the protocol at approximately 6-12 month intervals. A progress report will be forwarded to the DSMB at these times and their recommendations will be expeditiously implemented. All AEs and SAEs observed during the clinical trial and for which there is a relationship with the use of the investigational drugs, or the conduct of the study will be reported to the DSMB at the regularly scheduled DSMB meeting. The DSMB may recommend early termination of the study for considerations of safety and efficacy.

13.2 Assessment of Safety

Definitions

The definitions of the Reportable Events will be followed per Policy 801.

13.3 Documentation and Data Capture

Adverse event collection will start at the time of stem cell collection/bone marrow harvest. Grade 3 adverse events will be followed until satisfactory resolution.

Grade 3 and above AEs (serious and non-serious) will be captured in the research database from start of study treatment through final study visit.

Any serious adverse events that occur prior to the initiation of conditioning therapy will only be reported if at least possibly related to a pre-transplant procedure.

The Principal Investigator will be responsible for assessing AEs. Information on AEs will be solicited from subjects through questions from study personnel and information volunteered by the subject.

Following the completion of 5 years of follow-up care post BMT, only SAEs that are related to the bone marrow transplant procedure will be reported in the database.

13.4 Grading of Adverse Events

The AEs will be attributed (unrelated, unlikely, possibly, probably or definitely) to study medication and/or disease and graded by severity utilizing CTCAE version 4.0. A copy of the criteria can be down-loaded from the CTEP home page at http://ctep.cancer.gov/reporting/ctc.html.

**Grade**	Category	Description
**1**	Mild	Mild; asymptomatic; clinical or diagnostic observations only; intervention not Indicated
**2**	Moderate	Moderate; minimal, local or noninvasive intervention indicated; limiting age-appropriate instrumental ADL
**3**	Severe	Severe or medically significant but not immediately life-threatening; hospitalization or prolongation of hospitalization indicated; disabling; limiting self-care ADL
**4**	Life threatening	Life-threatening consequences; urgent intervention indicated
**5**	Death	Death related to AE

13.5 Attribution of Adverse Events

**Relationship**	**Attribution**	**Description**
Unrelated to investigational agent/intervention[1]	Unrelated	The AE *is clearly* ***NOT*** *related* to the intervention
	Unlikely	The AE *is* ***doubtfully related*** to the intervention
Related to investigational agent/intervention[1]	Possibly	The AE ***may be related*** to the intervention
	Probably	The AE ***is likely related*** to the intervention
	Definitely	The AE ***is clearly related*** to the intervention

[1]**NOTE**: AEs listed as 'possibly, probably, or definitely' related to the investigational agent/intervention are considered to have a suspected 'reasonable causal relationship' to the investigational agent/intervention (ICH E2A).

13.6 NIH Intramural IRB and CD Reporting

Expedited Reporting

Events requiring expedited reporting will be submitted to the IRB per Policy 801 "Reporting Research Events".

Reports to the IRB at the time of Continuing Review:

The PI or designee will refer to HRPP Policy 801 "Reporting Research Events" to determine IRB reporting requirements.

Reports to the CD:

The PI or designee will refer to NHLBI DIR Policy to determine CD reporting requirements and timelines.

Reports to the FDA:
The sponsor-representative or designee will refer to abbreviated IDE requirements of 21 CFR 812.2(b), to determine FDA reporting requirements and timelines.

HUMAN SUBJECT PROTECTIONS

14.1 Rationale for Subject Selection

All patients with confirmed sickle cell disease, as defined in section 5.1, will be considered for the protocol. Gender, ethnic background, and/or race will not be taken into consideration.

Strategies for patient recruitment: Information about the protocol will be posted on Clinicaltrials.gov, Clinical Center studies, and the NHLBI Patient recruitment websites. The protocol will also be listed in the physician's data query (PDQ).

14.2 Participation of Children

As stem cell transplant recipients

As the risk of our regimen in the haploidentical transplant setting is not known and is likely to be higher than our 6/6- HLA matched sibling transplant regimen, we will not include children on this protocol until the risk is more established.

As participants in laboratory research studies (donors)

A haploidentical pediatric donor may be the transplant subject's only option for a potentially curable treatment. Donor selection is done per standard of care. Should a transplant subject have a pediatric donor, the donors may participate in those laboratory studies that the IRB finds involves no greater than minimal risk to children provided that adequate provisions are made for soliciting the assent of the children and the permission of their parents or guardians.

14.3 Exclusion of Participants Unable to Provide Informed Consent and Participation of Adult Recipient Subjects Who Become Cognitively Impaired

All participants must be capable of providing informed consent. Participants not capable of providing informed consent, needing a legally authorized representative (LAR), will be excluded from participating in this study. The rationale for exclusion is while there may be direct benefit from participation, this is a pilot study with greater than minimal risk and this justifies requiring that each participant be able to provide informed consent.

However due to the nature of this disease, existing subjects who develop permanent cognitive impairment while still on study may remain on study. Maintaining their continued participation in this protocol is essential to increase understanding of all stages of the disease, as well as follow the natural progression of disease and/or help with identification of therapies. These subjects will receive an in depth evaluation, which would not otherwise be available to them outside of the NIH Clinical Center. The PI or the approved consenting AI will follow HRPP Policy 403 to consent the LAR.

14.4 Exclusion of Pregnant Women and Nursing Mothers

Adverse effects have been observed in animal reproduction studies related to several drugs being administered on this protocol, including sirolimus and hydroxyurea. Women of childbearing potential should be advised to avoid becoming pregnant. Women who are pregnant or breast-feeding are excluded due to the risk to the developing fetus or nursing babies. If a female patient becomes pregnant or suspects she is pregnant while on study, her treating physician should be immediately informed.

14.5 Hazards and Discomforts- Recipient

14.5.1 Related to the transplant

The mortality from conventional BMT may be as high as 40%. Although our data as well as that of others suggest a significant reduction in transplant-related mortality with nonmyeloablative PBSC transplantation, the procedure nevertheless carries significant risk. It is therefore only appropriate to carry out this experimental procedure in the context of debilitating or life-threatening conditions and with full informed

consent from the patient, donor, and immediate family. We have sought to develop a conditioning regimen which avoids the use of renally excreted drugs, and relies on the immunosuppressive effects of TBI as the basis for such an approach. The specific hazards of this study using a nonmyeloablative preparative regimen and high PBPC content graft are graft rejection, graft- versus-host disease, disease relapse, and infectious complications. The major discomforts are those of nausea, anorexia, diarrhea, fever, malaise, and intolerance of the isolation period. The 23 patients with SCD that were treated according to our previous haploidentical protocol at the NIH did not experience transplant- related mortality or severe GVHD.

The incidence of EBV-associated PTLD has been higher than expected on this protocol. As of December 20, 2022, 4 of 21 patients have developed PTLD. Two of the 4 patients responded to rituximab monotherapy; the other 2 patients required more aggressive therapy and therefore were categorized as having uncontrolled viral disease. To attempt to mitigate the risk, future patients transplanted on this protocol will be treated as those receiving umbilical cord HCT who are known to be at high risk for PTLD. According to NIH TCT Consortium EBV Management Guidelines, patients will be treated more vigorously with preemptive rituximab monotherapy. Further, according to the stopping rules already built into the protocol, the protocol will stop if the incidence of uncontrolled EBV PTLD is at most 15%.

In addition, the risk of a severe inflammatory disorder, hemophagocytic lymphohistiocytosis (HLH) is increased on this protocol. 3/21 patients have developed this complication. Two patients developed HLH following severe and refractory Evans syndrome. The third patient developed HLH related to EBV PTLD. As above, attempts have been made to mitigate the risk of Evans syndrome and EBV PTLD on this protocol.

Side effects of those drugs novel to nonmyeloablative transplantation are described in detail in the following “Boxed Warning.”

14.5.2 Related to Alemtuzumab

Boxed Warning

Hematologic Toxicity: Serious and, in rare instances fatal, pancytopenia/marrow hypoplasia, autoimmune idiopathic thrombocytopenia, and autoimmune hemolytic anemia have occurred in patients receiving Campath therapy. **Single doses of Campath greater than 30 mg or cumulative doses greater than 90 mg per week should not be administered because these doses are associated with a higher incidence of pancytopenia.**

Infusion Reactions: Campath can result in serious, and in some instances fatal, infusion reactions. Patients should be carefully monitored during infusions and Campath discontinued if indicated. **Gradual escalation to the recommended maintenance dose is required at the initiation of therapy and after interruption of therapy for 7 or more days.**

Infections, Opportunistic Infections: Serious, sometimes fatal bacterial, viral, fungal, and protozoan infections have been reported in patients receiving Campath therapy. Prophylaxis directed against *Pneumocystis carinii* pneumonia (PCP) and herpes virus infections has been shown to decrease, but not eliminate, the occurrence of these infections.

The safety and efficacy of alemtuzumab were evaluated in a multicenter, open-label, non-comparative study in 93 patients with refractory B-cell chronic lymphocytic leukemia (B-CLL) who had been previously treated with alkylating agents and had failed treatment with fludarabine, and side effects are detailed below. Previous treatment with alkylating agents and fludarabine may have contributed to both the range and severity of the side effects observed.

Infusion-related: Adverse events resulted in discontinuation of alemtuzumab therapy in 6% of the patients. The most commonly reported infusion-related adverse events include rigors in 89% of patients, drug-related fever in 83%, nausea in 47%, vomiting in 33%, and hypotension in 15%. Other frequently reported infusion-related events include rash in 30% of patients, fatigue in 22%, urticaria in 22%, dyspnea in 17%, pruritus in 14%, headache in 13%, and diarrhea in 13%. Acute infusion-related events were most common during the

first week of therapy. Antihistamines, acetaminophen, antiemetics, meperidine, and corticosteroids, as well as incremental dose escalation were used to prevent or ameliorate infusion-related events.

Infections: In the earlier studies all patients were required to receive anti-herpes and anti-PCP prophylaxis. Forty (43%) of 93 patients experienced 59 infections (one or more infections per patient) during treatment or within 6 months of the last dose. Of these, 34 (37%) patients experienced 42 infections that were of Grade 3 or 4 severity; 11 (18%) were fatal. Fifty-five percent of the Grade 3 or 4 infections occurred during treatment or within 30 days of the last dose. In addition, one or more episodes of febrile neutropenia (ANC 500 cells/μL) were reported in 10% of patients. The following types of infections were reported: Grade 3 or 4 sepsis in 12% of patients with one fatality, Grade 3 or 4 pneumonia in 15% with five fatalities, and opportunistic infections in 17% with four fatalities. Candida infections were reported in 5% of patients; CMV infections in 8% (4% of Grade 3 or 4 severity); Aspergillosis in 2% with fatal Aspergillosis in 1%; fatal Mucormycosis in 2%; fatal Cryptococcal pneumonia in 1%; Listeria monocytogenes meningitis in 1%; disseminated Herpes zoster in 1%; Grade 3 Herpes simplex in 2%; and Torulopsis pneumonia in 1%. PCP pneumonia occurred in one (1%) patient who discontinued PCP prophylaxis. In one of the earlier studies where anti-herpes and anti-PCP prophylaxis was optional, 37 (66%) patients had 47 infections while or after receiving Campath therapy.

Immunosuppression/Opportunistic Infections: Alemtuzumab induces profound lymphopenia. Anti-infective prophylaxis is recommended upon initiation of therapy and for a minimum of 2 months following the last dose of Alemtuzumab or until CD4+ counts are 200 cells/μL. The median time to recovery of CD4+ counts to 200/μL was 2 months, however, full recovery (to baseline) of CD4+ and CD8+ counts may take more than 12 months. Because of the potential for transfusion-associated GVHD in severely lymphopenic patients, irradiation of any blood products administered prior to recovery from lymphopenia is recommended.

Hematologic:

14.5.2.1 *Pancytopenia/Marrow Hypoplasia*: Alemtuzumab therapy was permanently discontinued in six (6%) patients due to pancytopenia/marrow hypoplasia. Two (2%) cases of pancytopenia/ marrow hypoplasia were fatal.

14.5.2.2 *Anemia:* Forty-four (47%) patients had one or more episodes of new onset NCI-CTC Grade 3 or 4 anemia. Sixty-two (67%) patients required RBC transfusions. In addition, erythropoietin use was reported in nineteen (20%) patients. Autoimmune hemolytic anemia secondary to Alemtuzumab therapy was reported in 1% of patients. Positive Coombs test without hemolysis was reported in 2%.

14.5.2.3 *Neutropenia*: Sixty-five (70%) patients had one or more episodes of NCI-CTC Grade 3 or 4 neutropenia. Median duration of Grade 3 or 4 neutropenia was 28 days (range: 2 – 165 days).

14.5.2.4 *Thrombocytopenia:* Forty-eight (52%) patients had one or more episodes of new onset Grade 3 or 4 thrombocytopenia. Median duration of thrombocytopenia was 21 days (range: 2 – 165 days). Thirty- five (38%) patients required platelet transfusions for management of thrombocytopenia. Autoimmune thrombocytopenia was reported in 2% of patients with one fatal case of Alemtuzumab -related autoimmune thrombocytopenia.

14.5.2.5 *Lymphopenia:* The median CD4+ count at 4 weeks after initiation of Alemtuzumab therapy was 2 (two)/μL, at 2 months after discontinuation of Alemtuzumab therapy, 207/μL, and 6 months after discontinuation, 470/μL. The pattern of change in median CD8+ lymphocyte counts was similar to that of CD4+ cells. In some patients treated with Alemtuzumab, CD4+ and CD8+ lymphocyte counts had not returned to baseline levels at longer than 1-year post therapy.

Cardiac: The following were reported in at least one patient treated on studies where Campath-1H was used as a single agent: cardiac failure, cyanosis, atrial fibrillation, cardiac arrest, ventricular arrhythmia, ventricular tachycardia, angina pectoris, coronary artery disorder, myocardial infarction, tears in artery linings of the head and neck and pericarditis. Some of these cardiac abnormalities may be irreversible. For

this reason, we will monitor subjects after the last dose of Campath- 1H and an echocardiogram and serum troponin level at the 3 month follow up visit. We will also closely monitor subjects for cardiac symptomology and ask them to immediately report any cardiac symptoms (palpitations, irregular pulse, difficulty in breathing, dizziness, swelling in the ankles, chest discomfort or pain).

Stroke: Patients with multiple sclerosis have rarely experienced strokes shortly after receiving alemtuzumab. 13 cases of ischemic and hemorrhagic stroke or arterial dissection were reported to the FDA from around the world between 2014 and 2018.

14.5.3 Related to Sirolimus:

The anticipated toxicities of sirolimus in this trial are those related to its immunosuppressive properties, such as an increased likelihood of infection, and mucosal (including mouth, gastric, small bowel, or large bowel) ulcers, which may bleed. Other possible toxicities are listed here and include those reported with > 3% and <20% incidence in patients in any Sirolimus treatment group in the two controlled clinical trials for the prevention of acute organ graft rejection:

Body as a Whole: abdomen enlarged, abscess, ascites, cellulitis, chills, face edema, flu syndrome, generalized edema, hernia, Herpes zoster infection, lymphocele, malaise, pelvic pain, peritonitis, sepsis;

Cardiovascular *System*: atrial fibrillation, congestive heart failure, hemorrhage, hypervolemia, hypotension, palpitation, peripheral vascular disorder, postural hypotension, syncope, tachycardia, thrombophlebitis, thrombosis, vasodilatation;

Digestive System: anorexia, dysphagia, eructation, esophagitis, flatulence, gastritis, gastroenteritis, gingivitis, gum hyperplasia, ileus, liver function tests abnormal, mouth ulceration, oral moniliasis, stomatitis;

Endocrine System: Cushing’s syndrome, diabetes mellitus, glycosuria, hypercholesterolemia, hyperlipidemia;

Hematologic and Lymphatic System: ecchymosis, leukocytosis, lymphadenopathy, polycythemia, thrombotic thrombocytopenic purpura / hemolytic-uremic syndrome;

Metabolic and Nutritional: acidosis, alkaline phosphatase increased, BUN increased, creatine phosphokinase increased, dehydration, healing abnormal, hypercalcemia, hyperglycemia, hyperphosphatemia, hypocalcemia, hypoglycemia, hypomagnesemia, hyponatremia, lactic dehydrogenase increased, SGOT increased, SGPT increased, weight loss;

Musculoskeletal System: arthrosis, bone necrosis, leg cramps, myalgia, osteoporosis, tetany, rhabdomyolysis;

Nervous System: anxiety, confusion, depression, dizziness, emotional lability, hypertonia, hypesthesia, hypotonia, insomnia, neuropathy, paresthesia, somnolence, stroke;

Respiratory System: dyspnea, changes in PFTs, asthma, atelectasis, bronchitis, cough increased, epistaxis, hypoxia, lung edema, pleural effusion, pneumonia, rhinitis, sinusitis, diffuse alveolar hemorrhage, angioedema;

Skin and Appendages: fungal dermatitis, hirsutism, pruritus, skin hypertrophy, skin ulcer, sweating; Special Senses: abnormal vision, cataract, conjunctivitis, deafness, ear pain, otitis media, tinnitus;

Urogenital System: albuminuria, bladder pain, dysuria, hematuria, hydronephrosis, impotence, kidney pain, kidney tubular necrosis, nocturia, oliguria, pyelonephritis, pyuria, scrotal edema, testis disorder, toxic nephropathy, urinary frequency, urinary incontinence, urinary retention.

Less frequently occurring adverse events included: mycobacterial infections, Epstein-Barr virus infections, BK virus-associated nephropathy, skin cancer, lymphoma, pericardial effusion, posterior reversible encephalopathy syndrome (PRES), and pancreatitis.

14.5.4 Related to Cyclophosphamide

Most commonly (>10%), patients may develop anorexia, nausea, vomiting, diarrhea, mucositis, myelosuppression, gonadal dysfunction, alopecia, and immunosuppression. Occasionally (1-10%), they may develop hemorrhagic cystitis, nasal stuffiness with rapid administration, flushing, rash, kidney tubular necrosis (which usually resolves with drug discontinuation) or SIADH. Rarely (<1%), patients may experience transient blurred vision, cardiac toxicity with arrhythmias, hyperpigmentation, impaired wound healing, myocardial necrosis, hepatotoxicity, weakness, hemorrhagic colitis, nail changes, bladder fibrosis, pulmonary fibrosis, and secondary malignancies.

14.5.5 Related to Pentostatin

Pentostatin is cleared by a renal mechanism (90%). As such, the pentostatin dose must be reduced for renal insufficiency (see 6.4). The primary toxicity is related to opportunistic infection due to T cell depletion or low blood counts. Common (>10%) side effects include fever, headaches, fatigue, nausea, vomiting, diarrhea, rash, pain, myelosuppression, and respiratory symptoms. Occasional (1-10%) side effects include changes in liver function tests, electrolytes, kidney function tests, blood pressure, heart rhythms; also chills, sweating, anxiety, confusion, dizziness, dry or itchy skin, or paresthesias. At higher doses, CNS toxicity may include seizures, coma, and death. Interstitial pulmonary toxicity or edema has also been described.

14.5.6 Related to Hydroxyurea

Hydroxyurea most commonly leads to myelosuppression, and blood counts will be frequently monitored during hydroxyurea administration. Less commonly, hydroxyurea can cause alopecia, dermatomyositis-like skin changes, hyperpigmentation, nail discoloration/atrophy, skin ulcers, drowsiness, anorexia, constipation, diarrhea, nausea, vomiting, and elevated hepatic enzymes. Rarely, hydroxyurea can cause edema, chills, fever, dizziness, disorientation, hallucinations, headache, malaise, seizure, facial/peripheral erythema, skin atrophy, hyperuricemia, pancreatitis, peripheral neuropathy, weakness, increased creatinine, pulmonary fibrosis, and acute diffuse pulmonary infiltrates. Secondary leukemias have been described after prolonged use in patients predisposed to developing leukemia.

14.5.7 Related to Radiation

Chest fluoroscopy, DEXA scan, and Total Body Irradiation (TBI): Recipient subjects will undergo Right Heart Catheterization with chest fluoroscopy, a DEXA radius/femur/spine-Ap exam, and Total Body Irradiation for research purposes as part of this study. If the right heart catheterization is performed using chest fluoroscopy instead of the MRI with contrast, then participants will be exposed to radiation. We anticipate that one right heart catheterization will be performed in applicable patients at 1 year post-transplant. DEXA Scans will be performed every 1 to 2 years depending on the severity of the bone disease. TBI will occur on Day-1 prior to stem cell transplantation.

Side effects of radiation have been well described[111]. The most common include nausea and mucositis. There also exists a risk of hypothyroidism, cataracts, interstitial pneumonitis, nephropathy, and an unspecified long term risk of developing secondary malignancies[112]. Importantly, the majority of the nonneoplastic effects were subclinical and/or reversible[113]. There is also a risk of sterility following TBI. Recovery of gonadal function has been reported to be 10-14% with a pregnancy incidence of <3% following TBI[114, 115]. However, these results are reported in patients who have history of hematologic malignancies, and therefore have also received prior chemotherapy. Further, the dose of TBI that they received was higher, at least 1000cGy. The incidence of sterility has not been reported in patients with nonmalignant hematologic diseases who have received lower doses of TBI. From our own experience, one patient with severe sickle cell disease who underwent an HLA- matched sibling peripheral blood stem cell transplant and received 300cGy TBI had a healthy baby 3 ½ years post-transplant. However, the risk of sterility exists, but is presumed to be lower than previously reported results. In a further attempt to decrease the risk of sterility, testicular shielding will be applied. We will discuss the option of gamete storage with subjects of child-bearing age. Studies attempting to evaluate the risk induced by radiation alone suggest that there is a higher rate of solid tumors after radiation

based regimens. Curtis et al. reported on 19,229 patients and found a cumulative incidence rate of 2.2% at 10 years, and 6.7% at 15 years, with higher doses of TBI associated with a higher risk of solid cancers. However, the more important risk factor appears to be related to the level of immunosuppression, as GVHD was also strongly linked to an increased risk of solid tumor development. In fact, some studies have shown no increased risk with radiation therapy[116, 117], but the highest risk factor was felt to be the presence of chronic GVHD and long term treatment with cyclosporine[118, 119]. Therefore the actual risk cannot be quantified for the low dose of 400cGy to be used in this trial; however the risk is presumed to be lower. Finally, the administration of the 400cGy as one fraction vs two divided fractions does carry a small increased risk of organ toxicity and mortality; however, because the total dose of radiation is low and because this single fractionated dose has been used safely in patients with sickle cell disease81 and beta thalassemia82 undergoing haploidentical HSCT, we believe this risk will be low.

The additional radiation exposure from DEXA Scans and fluoroscopy is negligible and is not expected to significantly increase the risk presented by TBI.

14.5.8 Related to Bone Marrow Harvest

Bone marrow harvest is performed under general anesthesia, and each patient will sign a separate consent for the bone marrow harvest and general anesthesia. Common risks associated with the procedure include back or hip pain, muscle pain, a new pain crisis, fatigue, headache, and bruising at the collection site. <3% of patients may experience a severe complication due to damage to nerve, bone, or muscle in their hip area.

14.5.9 Related to Antimicrobials in General

Allergic reactions, renal impairment (gentamicin, vancomycin, amphotericin, acyclovir), “red man” syndrome (vancomycin), hepatic damage (acyclovir, rifampicin).

14.5.10 Related to Bone Marrow Aspirate and Biopsy

No major risks are involved with bone marrow aspirate and biopsy. However, there is a small risk of infection, pain, bleeding, and hematoma formation at the site of the aspiration with the procedure.

14.5.11 Related to Blood Draws

No major risks are involved with blood draws. Minor complications including bleeding, pain, and hematoma formation at the site of blood draws; vasovagal reactions, thrombus formation, or infection may rarely occur.

14.5.12 Related to Cardiac Monitoring

EKG: An electrocardiogram (EKG) is a test that measures the electrical activity of the heartbeat. With each beat, an electrical impulse (or “wave”) travels through the heart. This wave causes the muscle to squeeze and pump blood from the heart. A technician will put patches (electrodes) on the chest, arms and legs. The electrodes are soft and don’t cause any discomfort when they’re put on or taken off by the technician. The machine only records the EKG. It doesn’t send electricity into the body. There’s no pain or risk associated with having an electrocardiogram

Transthoracic ECHO: The ECHO uses sound waves to visualize and evaluate the function of the heart. There are no associated risks

Holter Monitor: The Holter involves wearing a monitor for 24 hours during which time the electrical activity of the heart is recorded. There are no associated risks other than the inconvenience of wearing the apparatus.

14.5.13 Related to Central Line Placement

A catheter may be placed in a large vein of the neck, chest, or arm using local anesthetic. Patients will sign a separate consent for the line placement procedure. Only trained experienced staff will place the line in order to minimize these procedure-related risks. The risks from the procedure are low; they include bleeding, bruising, or infection at the site of insertion. Very rarely (less than 1% of the time), the line placement may nick a vein causing one lung to collapse during line insertion. If the lung collapses, a tube may have to be

inserted into the chest and remain in place until the lung re-expands. Because of this risk, patients will have a chest x-ray following the procedure to make sure the line is in the correct place and that the lung is not collapsed. Once placed, the line will remain in place until drug administration is complete.

14.5.14 Related to MRI without Contrast

MRI uses no ionizing radiation and is quite safe when performed on a properly screened population. Potential risks relate to the magnetic field's effect on patients with implanted metal objects (i.e. cerebral aneurysm clips, Cochlear implants, etc.). The magnetic field can cause twisting or movement of these objects thus causing harm to the patient. Also, the radiofrequency deposition of MRI can potentially cause burns in patients with pacemakers or other implanted coiled wires. Patients will be screened for these objects and will not undergo MRI if these objects are present and not compatible with MRI.

Although unlikely with our imaging parameters, peripheral nerve stimulation may cause discomfort. Peripheral nerve stimulation occurs when magnetic field gradient switching occurs fast enough to cause peripheral nerve depolarization. Typically, the patient may feel a twitch such as in the buttock, leg, or across the bridge of the nose. There is no physical danger from peripheral nerve stimulation. However, as gradient switching rates increase, smaller peripheral nerves (such as those that cause pain) can be excited. Precautions that will be taken include: 1) informing the subject of the possibility for peripheral nerve stimulation in the consent form, 2) empirically using parameters which maintain gradient switching rates below the pain threshold, and 3) audio feedback from the patient.

The switched gradients also generate noise in the scanner. Peak sound power produced in the magnet will be less than 140 dB and 119 dBA. The FDA limits constant noise exposure to 140 dB and 99 dBA for two hours. To stay within FDA guidelines on dBA limits all subjects will wear hearing protection in the form of earplugs and/or headphones. This is the procedure that is routinely done on the 1.5T and 3T MRI and it is well established to decrease noise levels by 20 dbA. There has been only one reported adverse effect on hearing in close to twenty years of MRI in the NIH NMR Center from noise.

There is no data that shows any significant adverse effects of exposure to static magnetic fields. There are well known minor adverse effects associated with high magnetic fields. These include nausea, metallic taste, and detection of flashes of light. All of these are associated with moving too rapidly in the magnetic field. To avoid these the subjects will be asked to walk slowly to the patient table and the table will be moved slowly into the magnet. Experiences at 3T show that these precautions eliminate these minor adverse reactions.

This protocol uses novel and custom sequences that control the MRI scanner to provide images with other characteristics than available from the product sequences provided by the vendor. The novel and custom sequences operate under the same safety limits as set by the FDA as product sequences. Therefore, there is no further risk to the subject compared to a clinical MRI.

For the subjects receiving gadolinium contrast agents, the placement of a peripheral intravenous line may result in mild discomfort, vasovagal reactions or bruising. If contrast is ordered, Gadolinium, an FDA approved medication will be used to improve MRI images. About 98% of patients receiving gadolinium have no symptoms related to the injection of this medication. Mild symptoms that may occur include: coldness in the arm at injection, a metallic taste, headache, and nausea. In an extremely small number of patients, more severe symptoms have been reported including: shortness of breath, wheezing, and lowering of blood pressure.

The US Food and Drug Administration has issued a warning that administration of gadolinium, the contrast imaging agent that may be used in this protocol, has been associated with development of a disease called nephrogenic systemic fibrosis. The syndrome is rare (approximately 200 cases reported worldwide as of December, 2006 out or several million administrations of gadolinium), but disabling and in some cases, fatal. All cases to date have occurred in patients with severe renal disease, including patients on dialysis. We will ask the patient whether they have or have had kidney disease or diabetes, whether they take diuretics

(water pills) for any medical condition and whether they have received x-ray dye or drugs recently that might affect their kidney function. Depending on their history and creatinine levels, it will be determined whether or not they may receive gadolinium.

14.5.15 Related to Right Heart Catheterization

The most common risks include bruising of the skin at the site where the catheter is inserted and excessive bleeding. Patients may experience some back discomfort or pain from having to lie still on the cardiac catheterization table for the length of the procedure. Rare complications may include abnormal heart rhythms, cardiac tamponade, infection, low blood pressure, blood clots at the tip of the catheter that can block blood flow, air embolism, pulmonary artery infarction, or pulmonary artery rupture. Patients will sign a separate consent before undergoing this procedure.

Additional risks will include those associated with the methodology used to perform the procedure, as it may be performed using chest fluoroscopy or MRI with contrast, as well as the use of inhaled nitric oxide. Please see below for the risks associated with these procedures and drugs.

14.5.16 Inhaled Nitric Oxide

Participants with elevated pulmonary artery resistance will undergo provocative testing with oxygen and nitric oxide as medically indicated. Nitric oxide inhalation is labeled for the treatment of neonates with hypoxic respiratory failure associated with pulmonary artery hypertension. However, it is widely used in the diagnosis and management of adults undergoing diagnostic cardiovascular catheterization and during critical illness. The proposed dose administered subacutely in a phase I study of healthy volunteers, inhaled nitric oxide 160 ppm, was associated with no adverse events[120]. Theoretical risks of inhaled nitric oxide include methemoglobinemia, exposure to cytotoxic nitrogen dioxide ($NO2$) that forms spontaneously in the dead space of tubing, and pulmonary edema. Observed risks during catheterization are negligible[121]. Participants with diastolic or systolic left ventricular dysfunction may experience elevation in left ventricular filling pressures during nitric oxide inhalation, manifest as dyspnea. The participant may request discontinuation of nitric oxide provocation because of dyspnea. Left ventricular filling pressures return promptly to baseline upon discontinuation of nitric oxide inhalation.

14.5.17 MRI and Gadolinium

Please see section 14.3.14 for risks associated with MRI with contrast if the right heart catheterization is performed with MRI with contrast instead of chest fluoroscopy.

14.5.18 Related to Iothalamate or Iohexol Testing

Participants may undergo iothalamate-based or equivalent GFR testing as described in section 6.3. There are 2 possible kinds of reactions to the radiographic contrast agent: dose-dependent, predictable physiochemical, intrinsic reactions (such as feelings of warmth) and rarely a dose-independent, unpredictable anaphylactoid reaction. The low contrast dose reduces the likelihood of intrinsic reactions but probably not the likelihood of an anaphylactoid reaction. Therefore, patients with allergy to iodine or iodinated contrast solutions, as manifested by urticaria or anaphylaxis, and individuals with asthma or history of asthma will be excluded. Subjects with a history of nausea or vomiting following administration of these agents will not be excluded since they do not correlate with increased chance of severe allergic reactions. The patients will sign a separate consent prior to receiving iothalamate or iohexol.

14.5.19 Related to Plerixafor

Plerixafor is an inhibitor of the CXCR4 chemokine receptor and blocks binding of its cognate ligand, stromal cell-derived factor-1a (SDF-1a). The CXCR4 and SDF-1a are recognized to play an important role in the homing of human HSCs to the marrow compartment, and the CXCR4 can help to anchor HSCs to the marrow matrix, including SDF-1α. Treatment with plerixafor resulted in leukocytosis and elevations in circulating HSCs, and the plerixafor-mobilized CD34+ cells were capable of engraftment with long-term repopulating capacity. The plerixafor blocks CXCR4 to directly interfere binding between HSCs and bone marrow niche,

while G-CSF markedly increases bone marrow cellularity with granulocyte expansion to permit egress of HSCs to peripheral blood[122]. The different mechanism of plerixafor results in less bone marrow expansion, less granulocyte stimulation, and lower peak of leukocyte counts than G-CSF[88, 122-125].

Common (>10%) side effects:

Plerixafor and G-CSF: The most common adverse reactions reported were diarrhea (37%), nausea (34%), fatigue (27%), injection site reactions (34%), headache (22%), arthralgia (13%), dizziness (11%), and vomiting (10%).

34% of patients had mild to moderate injection site reactions at the site of subcutaneous administration of plerixafor. These included erythema, hematoma, hemorrhage, induration, inflammation, irritation, pain, paresthesia, pruritus, rash, swelling, and urticarial.

Plerixafor only: Adverse events were similar, including lightheadedness (44%); nausea, bloating, or flatulence (36%); injection site discomfort or warm sensation (28%); perioral paresthesia, loose stools, or diaphoresis (20%); and headache (16%)[126]. A recent study for a single administration of plerixafor revealed mild-moderate facial paresthesia (26%) and mild arrhythmia including sinus tachycardia (56%), premature ventricular contraction (21%), and sinus bradycardia (7%)[127].

Less common (1-10%) side effects:

Other adverse reactions that occurred in < 5% of patients but were reported as related to plerixafor during HSC mobilization and apheresis included abdominal pain, hyperhidrosis, abdominal distention, dry mouth, erythema, stomach discomfort, malaise, hypoesthesia oral, constipation, dyspepsia, and musculoskeletal pain.

Rare (<1%) side effects:

Mild to moderate allergic reactions were observed in less than 1% of patients within approximately 30 min after plerixafor administration. Symptoms generally responded to treatments (e.g., antihistamines, corticosteroids, hydration or supplemental oxygen) or resolved spontaneously. Severe side effects including anaphylaxis have been reported in patients receiving plerixafor.

Vasovagal reactions, orthostatic hypotension, and/or syncope can occur following subcutaneous injections (<1%). The majority of these events occurred within 1 hour of plerixafor administration. Because of the potential for these reactions, appropriate precautions should be taken.

Other side effects may include thrombocytopenia, splenic enlargement (potential for rupture), fever, bone pain, anorexia, insomnia, muscle pain, and infusion related reaction

14.5 Hazards and discomforts- donor

14.5.1 Related to blood draws

No major risks are involved with blood draws. Minor complications including bleeding, pain, and hematoma formation at the site of blood draws, vasovagal reactions or infections may rarely occur.

14.6 Non-Significant Risk (NSR) Device Determination

The brain MRI/MRA will include novel and custom sequences that control the MRI scanner to provide images with other characteristics than available from the product sequences provided by the vendor.

The Philips 3T MRI machine used in this protocol is FDA approved and is used per FDA labelling as a data collection tool. All custom and research pulse sequences created by Vanderbilt University Medical Center (VUMC) that will be used as likewise within FDA approved specific absorption radiation (SAR) limits. Use of the research sequences will not exceed any of the limits deemed to represent Significant Risk by the FDA as defined in the guidance document, Criteria for Significant Risk Investigations of Magnetic Resonance Diagnostic

In addition, we will be use non-FDA approved MRI image reconstruction software (Phillips) and MRI image analysis software (VUMC). and The research pulse sequences, image reconstruction, and image analysis software used in this study meet the criteria for a non-significant risk device study, per FDA 21 CFR 812, because they:

• Are NOT intended as an implant and presenting a potential for serious risk to the health, safety, or welfare of a subject;

• Are NOT purported or represented to be for use supporting or sustaining human life and presenting a potential for serious risk to the health, safety, or welfare of a subject;

• Are NOT for a use of substantial importance in diagnosing, curing, mitigating, or treating disease, or otherwise preventing impairment of human health and presenting a potential for serious risk to the health, safety, or welfare of a subject; or

• Otherwise does NOT present a potential for serious risk to the health, safety, or welfare of a subject.

14.7 Risks in relation to benefit

14.7.1 For adult transplant subjects

Clinically, the approach is ethically acceptable because we are targeting a patient group with a debilitating and often lethal hematological disease, incurable with conventional treatments other than allogeneic BMT. The protocol aims to decrease the risk of transplant-related mortality, thus making more patients candidates for potentially curative therapy.

14.7.2 For pediatric donor participants involved in laboratory research studies

The inclusion of children satisfies the criteria set forth in 45 Code of Federal Regulations 46, Subpart D: 46.404 as follows:

The research does not involve greater than minimal risk. Blood specimens for research are obtained concurrently with clinically indicated sampling. Therefore, there is no risk associated with sample collection for research because research will only be performed on material obtained during standard clinical intervention.

Only those laboratory tests approved by the IRB and involving not greater than minimal risk will be conducted. Research will not include genetic testing. Therefore, there is no genetic testing-associated risk.

Adequate provisions are made for soliciting the assent of the children and permission of their parents or guardians, as set forth in 46.408.

14.7.3 For adult subjects who become cognitively impaired

Research subjects who develop cognitive impairment will continue to benefit from an in depth evaluation, which would not otherwise be available to them outside of the NIH Clinical Center.

14.8 Informed Consent

Informed consent will be conducted following OHSRP Policy 301- Informed Consent.

An IRB-approved consent form will be provided to the participant electronically or by hard copy for review prior to consenting. The initial consent process as well as re-consent, when required, may take place in person or remotely (e.g., via telephone or other NIH approved platforms). The investigational nature and objectives of this trial, the procedures, and their attendant risks and discomforts and potential benefits will be carefully explained to the participant in a private setting. The participant will be given as much time as they need to review the document and to consult with their family, friends, and personal health care providers. In addition, a study team member will be available to answer any questions.

A signed and dated informed consent document will be obtained by any investigator authorized to consent (See Key Study Personnel Page) prior to entry onto the study. Consent may be obtained with required

signatures on the hard copy of the consent or on the electronic document.

When a document that is in electronic format is used for obtaining consent, this study may use the iMed platform which is 21 CFR, Part 11 compliant, to obtain the required signatures.

During the consent process, participants and investigators may view the same approved consent document simultaneously when participant is being consented in person at the Clinical Center or both may view individual copies of the approved consent document on screens in their respective locations remotely. Signatures may be obtained either by both directly signing on the device that the consenting investigator is using (when in person) or through iMed Mobile Signature Capture (remotely) which allows texting or emailing a link to the participant. That link allows the participant to review the consent, then proceed to sign on the device they are using.

Whether hard copy or electronic, both the investigator and the participant will sign the document with a hand signature using a pen (if using hard copy), finger, stylus, or mouse (if electronic). When done remotely, if the participant prefers to sign a hard copy, they may be instructed to sign and date the consent document during the discussion and mail, secure email or fax the signed document to the consenting investigator. Whether in person or remotely, the privacy of the participant will be maintained. Finally, the fully signed informed consent document will be stored in the electronic medical record, and the participant will receive a copy of the signed informed consent document.

If the donor is a minor, the parent who signs the consent for the minor must be a legally recognized parent or guardian. Where deemed appropriate by the clinician, and the child's parent or guardian, the child will also be included in all discussions about the trial and a minor's assent will be obtained. The parent or guardian will sign on the designated line on the informed consent attesting to the fact that the child had given assent.

In cases where parents share joint legal custody for medical decisions for a child (e.g., by custody agreement or court order), both parents must give their permission regardless of the level of risk of the research. Exceptions may be made if one parent has since died, becomes incompetent, or is not reasonably available (e.g., incarcerated).

Assent Procedures

Participants 14 years of age or older will review and discuss the adult consent with the parents and research team, and sign the standard adult consent in the assent line. If a minor is between the ages of 7 and 13 years of age, then the minor will sign the minor assent form. Minors under 7 years of age will provide verbal assent for participation in this study.

Consent for Minors when they reach the age of majority

When a pediatric subject reaches age 18, continued participation will require consenting of the now adult with the standard protocol consent document to ensure legally effective informed consent has been obtained. Should sample or data analysis continue following completion of active participation and the subject has reached 18 years of age, we will attempt to contact the subject using the last known contact information to obtain consent for continued use of data or samples collected during their prior visit. Given the length of time that may have transpired for some of the subjects since their last visit for this study, we request waiver of informed consent for those individuals who after good faith efforts to contact them, we are unable to do so and for subjects who are off study.

Requirements for Waiver of Consent consistent with 45 CFR 46.116 (d):

The research involves no more than minimal risk to the subjects.

Analysis of samples and data from this study involves no additional risks to subjects.

As the research involves using identifiable private information or identifiable biospecimens, the research could not practicably be carried out without using such information or biospecimens in an identifiable

format.

Though the purpose of future studies cannot yet be known, they often involve the correlation of clinical outcomes and clinical interventions with laboratory studies. Such information would be unavailable if access to medical record numbers was unavailable.

The waiver or alteration will not adversely affect the rights and welfare of the subjects.

Retention of these samples or data does not affect the welfare of subjects.

Whenever appropriate, the subjects will be provided with additional pertinent information after participation.

We only plan to request a waiver of reconsent for those subjects who have been lost to follow-up.

At any time during participation in the protocol that new information becomes available relating to risks, adverse events, or toxicities, this information will be provided orally or in writing to all enrolled or prospective patient participants. Documentation will be provided to the IRB and if necessary, the informed consent amended to reflect relevant information.

Adult Subjects Who Become Cognitively Impaired

Subjects who become unable to consent due to complications (permanent or declining) from SCD may remain on this study. During all visits, study personnel will observe the subjects for any signs of loss of cognitive function. If observed, the PI or AI will contact the NIH Ability to Consent Assessment Team (ACAT) for evaluation as needed for the following: an independent assessment of whether an individual has the capacity to provide consent; assistance in identifying and assessing an appropriate surrogate when indicated; and/or an assessment of the capacity to appoint a surrogate. For those subjects that become incapacitated and do not have pre-determined substitute decision maker, the procedures described in NIH HRPP Policy 403 for appointing a surrogate decision maker for adult subjects who are (a) decisionally impaired, and (b) who do not have a legal guardian or durable power of attorney, will be followed.

15.0 COMPENSATION

Subjects participating in the quality of life portion of the study may receive up to $60 USD per year ($80 USD in the first year) for their time and inconvenience for completion of questionaries which vary in length (see table below). Payments will be made to the subject by debit card or direct deposit upon completion of questionnaires. If the subject is unable to complete the study, they will still receive compensation for the surveys completed.

**Financial Compensation Details**				
**Procedure**	**Frequency**	**Compensation per Test**	**Male Total**	**Female Total**
**PROMIS Quality of Life Questionnaire (male and female)**	**once / year, *D+100**	**$20**	**$20***	**$20***
**Changes in Sexual Function Questionnaire (male and female)**	**once / year**	**$20**	**$20**	**$20**

Fertility Survey (females only)	once /year	$20	N/A	$20
Priapism Impact Questionnaire (males only)	once / year	$10	$10	N/A
International Index of Erectile Function (males only)	once / year	$10	$10	N/A
TOTAL (USD)			$60	$60

*****In the first year subjects may receive up to $80 if the Day 100 PROMIS survey is completed along with the other specified surveys for that timepoint.*

16.0 STATISTICAL CONSIDERATIONS

We propose to test a novel allogeneic PBSCT regimen using haploidentical family donors, incorporating pre- transplant PC in an attempt to reduce the risk of graft rejection and one dose of post-transplant cyclophosphamide to try to decrease the risk of graft rejection and GVHD in high risk patients with SCD. The primary endpoint for each individual is a dichotomous positive/negative outcome where a positive response is defined by absence of graft rejection, and absence of severe acute GVHD (grade 3 and higher), or moderate to severe chronic GVHD evaluated 100 days post-transplant. Any other outcome is a negative (i.e. failed) response. Transplanted individuals who do not provide an endpoint outcome (primarily due to dropout or death) will be treated as having a failed response. If an enrolled participant dies before transplant for reasons unrelated to transplant or is otherwise unable to be transplanted, up to 5 recipient subjects will be replaced (and respectively up to 5 donor participants). Therefore, the accrual ceiling for this protocol is up to 98 subjects total (49 recipients and 49 donors).

Stopping Rules and Sample Size

The study is designed to determine if the primary outcome success rate shows preliminary efficacy warranting further development. Because the 11 subjects enrolled on haploidentical protocol 09-H-0225 who acutely rejected their grafts did so by 100 days post-transplant, stopping rules for graft rejection will be monitored at 100 days post- transplant. Let p denote the probability of success for the primary outcome – the absence of graft rejection and no severe GVHD at 100 days. We feel a successful transplant regimen should have a success probability of at least 70%. Formally we test the null hypothesis that p is less than or equal to 0.50 versus the alternative hypothesis that p is at least 0.70. The trial is designed to enroll 39 individuals and if 25 or more have a successful primary outcome we conclude further development may be warranted, based on efficacy (further consideration for safety concerns are presented below). With this sample size and criteria, if the true success rate is 70% or more than there is 80% power for concluding further development is possibly warranted. If the true success rate is 50% or less, then there is only a 5% probability we will erroneously conclude further development is warranted. Formally, we test the null hypothesis that $p \leq 0.50$ with alpha = 0.05 and the study has 80% power for the alternative hypothesis that $p \geq 0.70$. We use a variation of the two-stage minimax design[128, 129] which also allows for early stopping of the study if the success rate is low among early enrollees.

Two intermediate stages are included to provide early stopping opportunities if early enrollees show low success rates. If 5 or more failures are observed among the first 8 enrollees the trial will be stopped. If fewer than 5 failures are observed, then the next intermediate stage is evaluated after 15 patients. If 8 or more failures among the first 15 patients are observed, then the study will stop. If fewer than 8 failures are observed at this stage, then the study will continue to the full cohort of 39 as long as no more than 15 patients fail (if 15 or more patients fail then less than 25 of 39 patients can have success). The study will stop early (before

full enrollment of 39) if the 15th patient fails before enrollment is complete. This design is reflected in the table below.

Group Monitoring Plan

Number of patients accrued	Number of regimen failures triggering stopping
≤ 8	5
9-15	8
16-39	15

Because the majority of viral disease occurs by 100 days post-transplant[130, 131] and because acute GVHD is a major risk factor for chronic GVHD[132, 133] such that we expect patients that experience GVHD will do so by day 100 post- transplant, these stopping rules will be assessed at 100 days post-transplant. An additional safety stopping rule will include any grade III or higher adverse events (CTCAE version 4.0) considered possibly or definitely related to bone marrow harvest (with the exclusion of pain crisis). Acute chest syndrome will be included in this stopping rule.

Therefore, a stopping boundary is in place for any of the following qualifying adverse events: severe acute or chronic GVHD, viral disease not controllable by anti-viral therapy, or grade III or higher adverse events considered possibly or definitely related to bone marrow harvest (with the exclusion of pain crisis) within 100 days post- transplant. If any of these outcomes occurs, the participant will be counted as having a qualifying adverse event. A Bayesian stopping rule is proposed in which stopping the trial will be recommended if the Bayesian posterior probability that the true probability of a qualifying AE exceeds 25% is at least 75% (the prior parameters for the beta distribution are a=0.5 and b= 4.5).

Number of patients accrued	Number of qualifying AEs to trigger stopping
2-3	2
4-6	3
7-9	4
10-13	5
14-16	6
17-20	7
21-24	8
25-27	9
28-31	10
32-34	11
35-38	12
39	13

In addition to this boundary that counts any occurrence of the three qualifying AEs (severe acute or chronic GVHD, viral disease uncontrollable by anti-viral therapy, or grade III or higher adverse events considered possibly or definitely related to bone marrow harvest (with the exclusion of pain crisis), there are separate boundaries for each of the three qualifying events. If counts for any of these individual AEs reach the following boundaries there will be a recommendation to stop the study. The boundary is calculated so the Bayesian posterior probability that the true probabilities of the individual AE exceeds 15% is at least 75%

(the prior parameters for the beta distribution are a=1 and b= 4). The table showing the number of a particular type of AE that will trigger a stop recommendation is shown below.

In addition, the study will be stopped if three patients develop refractory or severe Evans syndrome or if there is a fourth case of severe, refractory HLH post-transplant.

Number of patients accrued	Number of qualifying AEs of the same type to trigger stopping
2-5	2
6-10	3
11-15	4
16-21	5
22-27	6
27-33	7
34-39	8

Stopping rules will be monitored after every 3 accrued SCD patients reach 100 days post-transplant. If the stopping boundaries are reached, accrual of subjects to the protocol will cease and no further subjects will be transplanted. Accrual to the protocol will be held at the time of any death or if 2 patients experience myelodysplastic syndrome, acute leukemia, or chronic leukemia, until independent review confirms that it is safe to continue the protocol.

Statistical analysis for secondary outcomes

The Kaplan–Meier method and cumulative incidence curves will be used to analyze incidence of acute and chronic GVHD, disease-free survival and overall survival, viral disease, relapse rate and graft rejection rate, and transplant-related mortality. The Log rank test will be used to test the difference in time to event outcomes among different types of donors although power will likely be low given relatively small numbers of events are expected.

The change of biomarkers at specific time point will be compared with paired t-test or corresponding non-parametric methods. Linear mixed models or generalized linear mixed models will be used to assess the time course of biomarkers.

Statistical considerations related to changing the radiation regimen

As of December 31, 2020, 19 patients had been transplanted on this study using a two-dose TBI regimen. With the approval of *Amendment Q* to the protocol, the radiation regimen changed to a single dose TBI regimen to improve long term graft survival. This change is expected to primarily affect the long-term patency of the graft rather than the primary outcome, i.e. successful engraftment without GVHD assessed at 100 days post-transplant. Although the change in radiation procedures is not expected to materially affect the 100 day primary outcome, separate results from the initial cohort of 19 with the two dose regimen and the subsequent cohort of 20 with the one dose regimen will be presented, as well as a test for a significant difference between the two cohorts for the primary outcome.

Further, as shown in the power analysis in Appendix H, it is unlikely this test will detect a difference in the failure probabilities between the two regimens. In this case we will accordingly present the failure probability for the combined cohorts as the primary analysis although the results will also be presented separately for the two cohorts. In our manuscript it will be acknowledged that any lack of a statistically significant difference in failure probabilities may be in part a consequence of the small sample sizes in the two cohorts.

In addition to this analysis of the primary endpoint at 100 days, secondary endpoints associated with the regimen will include:

Graft failure incidence, separated by radiation regimen

GVHD incidence, separated by radiation regimen

Disease-free survival and overall survival, separated by radiation regimen

Incidence of viral reactivation and disease, separated by radiation regimen

Duration of full donor chimerism (defined as donor myeloid chimerism and donor CD3 chimerism $\geq$95%), separated by radiation regimen

Differences by regimen in these secondary outcomes will be assessed graphically and by log-rank tests but statistical power will likely be low given the expectation of relatively few events.

Radiation Dosing Change Stopping Rule

The stopping rules described above will continue to be followed by cumulatively accruing patients with the single dose TBI and their events to those already observed among the first 19 participants. Although we do not expect the change in radiation to impact safety outside of the already established stopping rules for GVHD and viral disease we have added an additional stopping rule that will apply only to those treated after the amendment's implementation. i.e. those receiving the single dose TBI.

This stopping rule mirrors one above in that it counts severe acute or chronic GVHD, viral disease not controllable by anti-viral therapy, or grade 3 or higher adverse events considered possibly or definitely related to bone marrow harvest (with the exclusion of pain crisis) within 100 days post- transplant. If any of these outcomes occurs, the participant will be counted as having a qualifying adverse event. A Bayesian stopping rule is proposed in which stopping the trial will be recommended if the Bayesian posterior probability that the true probability of a qualifying AE exceeds 25% is at least 75% (the prior parameters for the beta distribution are a=0.5 and b= 4.5). Because 19 individuals have already been treated with the two dose regimen, this stopping rule can be applied to a maximum of 39-19 = 20 individuals who receive the single dose regimen.

Number of patients accrued	Number of qualifying AEs to trigger stopping
2-3	2
4-6	3
7-9	4
10-13	5
14-16	6
17-20	7

17 ON STUDY DATE: Date of Consent Signing

18 OFF STUDY CRITERIA

Withdrawal by the patient from the transplant procedure

Patients and their donors will be given ample opportunity to withdraw from the study prior to admission for transplant. Thereafter, the nature of the procedure does not permit safe withdrawal from the protocol. The patient and donor have the right at any time to elect not to participate in the research aspects of the protocol (donation of blood and bone marrow for non-routine tests).

Withdrawal by the physician from experimental protocol treatment

Patients with disease relapse may be taken off study treatment, based on clinical judgement. The patient will then receive alternative treatments or will be referred back to his/her referring physician depending on what is considered to be in the best interest of the patient. Subjects will continue to be monitored by our institution post- transplant for possible transplant-related complications related to the conditioning regimen, disease-free survival, overall survival, and transplant-related mortality. Patients will be taken off study after a death, if they are lost to follow-up, or per PI discretion. If a patient is taken off study prior to transplant they can rescreen later and will need to have protocol eligibility re-confirmed and subsequently reconsent onto the protocol. In these cases, the subject number will remain the same. If research samples and research procedures were already collected/performed it is not required that they be repeated. Other baseline tests that are not done for eligibility will be assessed by the PI and only repeated if required per PI.

19 PHARMACEUTICALS

19.1 Off-Label Use of Drugs

Alemtuzumab and pentostatin will be used beyond the indications specified in the Prescriber Information. These Off-Label uses will comply with prevailing community standards.

The use of these drugs for this protocol meets the requirements for exemption from the Investigational New Drug regulations, 21 CFR 312, specifically:

The investigational drug is lawfully marketed in the United States

The investigation is not intended to be reported to the FDA as a well-controlled study in support of a new indication for use of the drug product

The investigation is not intended to support a significant change in advertising to an existing lawfully marketed prescription drug product

The investigation does not involve a route of administration or dosage level or other factor that significantly increases the risks (or decreases the acceptability of the risks) associated with the use of the drug product.

The investigation will be conducted in compliance with the requirements for institutional review set forth in FDA regulations 21 CFR 56, and requirements for informed consent as set forth in FDA regulations 21 CFR 50
The investigation will be conducted in compliance with FDA regulations 21 CFR 312.7: Promotion and charging for investigational drugs.

19.2 Alemtuzumab

Generic: alemtuzumab

Classification: monoclonal antibody

Action: Monoclonal antibody directed against CD52 antigen, a surface glycoprotein expressed by lymphocytes

Availability: Campath Distribution Program; Genzyme Corporation

Supply: Available through the Campath Distribution Program (1-877-422-6728). Vials are provided through this program upon completion of a patient specific request form. Prior to submission of a drug request the patient must provide authorization for the release of medical information (NIH-527). Refer to the Pharmacy Department or Clinical Pharmacy Specialist for additional details on drug procurement.
Storage: Stored at 2 to 8 degrees Celsius (36 to 46 degrees Fahrenheit) and protected from direct sunlight.

Protect from freezing; discard if frozen.

Stability: Diluted solution for administration can be stored at room temperature (15 to 30 degrees Celsius)

or refrigerated, and should be used within 8 hours after dilution; protect solution from light.

Product Description: Injection; available in 30 mg/1 mL single use vials

Preparation: The drug product should be visually inspected for particulate matter and discoloration prior to administration. If particulate matter is present or the solution is discolored, the vial should not be used. The vial should not be shaken. Contents should be injected into 100mL sterile 0.9% sodium chloride and the bag should be gently inverted to mix the solution. Alemtuzumab should be used within 8 hours after dilution.

Route: Intravenous

19.3 Cyclophosphamide Generic: cyclophosphamide Classification: alkylating agent

Action: Alkylates and crosslinks DNA *Availability:* Commercially available *Storage:* Stored at room temperature.

Stability: Once reconstituted as directed, solutions of cyclophosphamide are stable for 24 hours at room temperature, or 6 days when refrigerated at 2-8º C.

Product description: Injection or for oral use as capsules providing 25 mg or 50 mg of cyclophosphamide.

Preparation: Cyclophosphamide powder for injection should be reconstituted with sterile water for injection to yield a concentration of 20 mg/mL as described in the product labeling. Once reconstituted, the prescribed dose will be further diluted in 100 to 250 mL of 0.9% sodium chloride injection or 5% dextrose in water for intravenous administration over 60 +/- 10 minutes.

Route: Intravenous

19.4 Pentostatin

Supply: Commercially available

Product description: Pentostatin is available as a lyophilized powder in vials containing 10 mg of drug.

Preparation: The lyophilized powder will be reconstituted according to manufacturer instructions, into a solution of a 2 mg/ml concentration. The appropriate dose of the reconstituted pentostatin solution will be further diluted with 500 ml of 0.9% sodium chloride and infused over 60 +/- 10 minutes.

Storage and Stability: Upon reconstitution, the pentostatin can be stored at room temperature but should be used within 8 hours of reconstitution.

Route of administration: Intravenous infusion. Subjects will receive one liter of 0.9% sodium chloride by intravenous infusion as pre-hydration prior to the pentostatin delivery. Pentostatin will be infused over 60 +/- 10 minutes.

19.5 Hydroxyurea

Generic: hydroxyurea

Supply: commercially available

Classification: antineoplastic agent, antimetabolite

Action: Selectively inhibits ribonucleoside diphosphate reductase, halting the cell cycle at the G1/S phase.

Storage and stability: Oral capsules should be stored at controlled room temperature 15-30ºC (59-86ºF).

Product description: Hydroxyurea is available for oral use as capsules providing 200 mg, 300 mg, 400 mg and 500 mg of hydroxyurea. The 200 mg, 300 mg, and 500 mg capsules are stocked by the NIH Clinical Center Pharmacy. *Route*: oral administration

19.6 Sirolimus

Generic: sirolimus

Classification: mammalian Target of Rapamycin (mTOR) inhibitor

Action: Inhibits T-lymphocyte activation and proliferation and also inhibits antibody production.

Availability: Commercially available

Storage: Tablets should be stored at 20º to 25º C. Cartons should be used to protect blister cards and strips

from light. Sirolimus should be dispensed in a tight, light-resistant container.

Stability: Studies on batches of sirolimus, which had been stored at 5º C and 25º C for 18 months in a package simulating the bulk storage package, were performed and did not reveal any significant changes in sirolimus stability.

Product description: Tablets (0.5 mg, 1 mg, 2 mg tablets) or oral solution (1 mg/mL).

Route: Sirolimus is administered orally.

19.7 Plerixafor

Generic: plerixafor

Classification: hematopoietic stem cell mobilizer

Action: Reversibly inhibits binding of SDF-1a to CXCR4, resulting in mobilization of hematopoietic stem and progenitor cells into peripheral blood.

Storage and stability: The intact vials of plerixafor should be stored at 25C (77F); excursions permitted to 15-30C (59-86F). The expiration date is indicated on the label. Do not use it after the expiration date.

Product description: Plerixafor is available from the Clinical Center pharmacy in single-use clear glass vials containing 24 mg plerixafor in 1.2 mL vials (20 mg/mL). It is formulated as a sterile, preservative-free, clear, colorless to pale yellow, and isotonic solution for subcutaneous injection. The quantitative composition (per vial) is:

Plerixafor	24 mg
Sodium chloride	5.9 mg

Water for injection (qs ad) to 1.2 mL (adjusted to a pH of 6.0 to 7.5 with hydrochloric acid and with sodium hydroxide)

Route: Plerixafor is administered intravenously.

Center for International Blood and Marrow Transplant Research (CIBMTR)

For the purposes of quality assurance (i.e. accreditation of the NHLBI Transplant program), anonymized data will be released to the CIBMTR according to Federally mandated policies and procedures.

21 REFERENCES

1. Platt OS, Brambilla DJ, Rosse WF, et al. Mortality in sickle cell disease. Life expectancy and risk factors for early death [see comments]. *The New England journal of medicine*. 1994;330(23):1639-44.
2. Feld JJ, Kato GJ, Koh C, et al. Liver injury is associated with mortality in sickle cell disease. *Aliment Pharmacol Ther*. Oct 2015;42(7):912-21. doi:10.1111/apt.13347
3. Wayne AS, Schoenike SE, Pegelow CH. Financial analysis of chronic transfusion for stroke prevention in sickle cell disease. *Blood*. Oct 1 2000;96(7):2369-72.
4. Rodgers GP, Dover GJ, Noguchi CT, Schechter AN, Nienhuis AW. Hematologic responses of patients with sickle cell disease to treatment with hydroxyurea. *N Engl J Med*. 1990;322(15):1037-45.
5. Charache S, Terrin ML, Moore RD, et al. Effect of hydroxyurea on the frequency of painful crises in sickle cell anemia. Investigators of the Multicenter Study of Hydroxyurea in Sickle Cell Anemia. Clinical Trial
Multicenter Study
Randomized Controlled Trial
Research Support, Non-U.S. Gov't
Research Support, U.S. Gov't, P.H.S. *The New England journal of medicine*. May 18 1995;332(20):1317-22. doi:10.1056/NEJM199505183322001
6. Ferster A, Vermylen C, Cornu G, et al. Hydroxyurea for treatment of severe sickle cell anemia: a pediatric clinical trial. *Blood*. 1996;88(6):1960-4.
7. Steinberg MH, Barton F, Castro O, et al. Effect of hydroxyurea on mortality and morbidity in adult sickle cell anemia: risks and benefits up to 9 years of treatment. Clinical Trial

Multicenter Study
Randomized Controlled Trial
Research Support, U.S. Gov't, P.H.S. *JAMA : the journal of the American Medical Association*. Apr 2 2003;289(13):1645-51. doi:10.1001/jama.289.13.1645
8. Walters MC, Patience M, Leisenring W, et al. Bone Marrow Transplantation for Sickle Cell Disease. *The New England journal of medicine*. August 8, 1996 1996;335(6):369-376.
9. Vermylen C, Cornu G, Ferster A, et al. Haematopoietic stem cell transplantation for sickle cell anaemia: the first 50 patients transplanted in Belgium. *Bone Marrow Transplant*. Jul 1998;22(1):1-6.
10. Bernaudin F, Socie G, Kuentz M, et al. Long-term results of related myeloablative stem-cell transplantation to cure sickle cell disease. *Blood*. Oct 1 2007;110(7):2749-56. doi:blood-2007-03-079665 [pii] 10.1182/blood-2007-03-079665
11. Weiden PL, Sullivan KM, Flournoy N, Storb R, Thomas ED. Antileukemic effect of chronic graft-versus-host disease: contribution to improved survival after allogeneic marrow transplantation. *The New England journal of medicine*. 1981;304(25):1529-33.
12. van den Bos C, Kieboom D, Wagemaker G. Correction of murine beta-thalassemia by partial bone marrow chimerism: selective advantage of normal erythropoiesis. *Bone Marrow Transplant*. 1993;12(1):9-13.
13. Andreani M, Manna M, Lucarelli G, et al. Persistence of mixed chimerism in patients transplanted for the treatment of thalassemia. *Blood*. 1996;87(8):3494-9.
14. Walters MC, Patience M, Leisenring W, et al. Stable mixed hematopoietic chimerism after bone marrow transplantation for sickle cell anemia. *Biol Blood Marrow Transplant*. 2001;7(12):665-73.
15. Huss R, Deeg HJ, Gooley T, et al. Effect of mixed chimerism on graft-versus-host disease, disease recurrence and survival after HLA-identical marrow transplantation for aplastic anemia or chronic myelogenous leukemia. *Bone Marrow Transplant*. 1996;18(4):767-76.
16. Kang EM, Areman EM, David-Ocampo V, et al. Mobilization, collection, and processing of peripheral blood stem cells in individuals with sickle cell trait. *Blood*. Feb 1 2002;99(3):850-5.
17. Abboud MR, Jackson SM, Barredo J, Holden KR, Cure J, Laver J. Neurologic complications following bone marrow transplantation for sickle cell disease. *Bone Marrow Transplant*. 1996;17(3):405-7.
18. Walters MC, Patience M, Leisenring W, et al. Barriers to bone marrow transplantation for sickle cell anemia. *Biol Blood Marrow Transplant*. 1996;2(2):100-4.
19. Walters MC, Sullivan KM, Bernaudin F, et al. Neurologic complications after allogeneic marrow transplantation for sickle cell anemia [see comments]. *Blood*. 1995;85(4):879-84.
20. Marijt WA, Veenhof WF, Goulmy E, Willemze R, van Rood JJ, Falkenburg JH. Minor histocompatibility antigens HA-1-, -2-, and -4-, and HY-specific cytotoxic T-cell clones inhibit human hematopoietic progenitor cell growth by a mechanism that is dependent on direct cell-cell contact. *Blood*. Dec 15 1993;82(12):3778-85.
21. Vogt MH, Goulmy E, Kloosterboer FM, et al. UTY gene codes for an HLA-B60-restricted human male-specific minor histocompatibility antigen involved in stem cell graft rejection: characterization of the critical polymorphic amino acid residues for T-cell recognition. *Blood*. Nov 1 2000;96(9):3126-32.
22. Hsieh M, Wilder, J, Fitzhugh, C, Link, B, Tisdale, JF. Results of Alternative Donor Search in Adult Patients with Severe Sickle Cell Disease (SCD) Eligible for Hematopoietic Stem Cell Transplantation (HSCT). 2007:670a.
23. Jaing TH, Sun CF, Lee WI, Wen YC, Yang CP, Hung IJ. Successful unmanipulated peripheral blood progenitor cell transplantation from an HLA haploidentical 2-locus-mismatched mother in a thalassemic patient with primary graft failure after transplantation of bone marrow and cord blood from unrelated donors. Case Reports. *Pediatr Transplant*. Mar 2008;12(2):232-4. doi:10.1111/j.1399-3046.2007.00844.x
24. Hongeng S, Pakakasama S, Chaisiripoomkere W, Ungkanont A, Jootar S. Nonmyeloablative stem cell transplantation with a haploidentical donor in a class 3 lucarelli severe thalassemia patient. Case Reports Letter. *Bone Marrow Transplant*. Aug 2004;34(3):271-2. doi:10.1038/sj.bmt.1704572
25. Walters MC, Sullivan KM, O'Reilly RJ, et al. Bone marrow transplantation for thalassemia. The USA experience. *Am J Pediatr Hematol Oncol*. Feb 1994;16(1):11-7.
26. Hobbs JR, Hugh-Jones K, Shaw PJ, Downie CJ, Williamson S. Engraftment rates related to busulphan

and cyclophosphamide dosages for displacement bone marrow transplants in fifty children. *Bone Marrow Transplant*. Dec 1986;1(2):201-8.
27. Mugishima H, Ohga S, Ohara A, Kojima S, Fujisawa K, Tsukimoto I. Hematopoietic stem cell transplantation for Diamond-Blackfan anemia: a report from the Aplastic Anemia Committee of the Japanese Society of Pediatric Hematology. *Pediatric transplantation*. Sep 2007;11(6):601-7.
28. Castro O, Hoque M, Brown BD. Pulmonary hypertension in sickle cell disease: cardiac catheterization results and survival. *Blood*. Feb 15 2003;101(4):1257-61.
29. Gladwin MT, Sachdev V, Jison ML, et al. Pulmonary hypertension as a risk factor for death in patients with sickle cell disease. *The New England journal of medicine*. Feb 26 2004;350(9):886-95.
30. Prasad R, Hasan S, Castro O, Perlin E, Kim K. Long-term outcomes in patients with sickle cell disease and frequent vaso-occlusive crises. *The American journal of the medical sciences*. Mar 2003;325(3):107-9.
31. Dick AD, Meyer P, James T, et al. Campath-1H therapy in refractory ocular inflammatory disease. *Br J Ophthalmol*. 2000;84(1):107-9.
32. Coles AJ, Wing M, Smith S, et al. Pulsed monoclonal antibody treatment and autoimmune thyroid disease in multiple sclerosis. *Lancet*. 1999;354(9191):1691-5.
33. Isaacs JD, Burrows N, Wing M, et al. Humanized anti-CD4 monoclonal antibody therapy of autoimmune and inflammatory disease. *Clin Exp Immunol*. 1997;110(2):158-66.
34. Killick SB, Marsh JC, Hale G, Waldmann H, Kelly SJ, Gordon-Smith EC. Sustained remission of severe resistant autoimmune neutropenia with Campath-1H. *Br J Haematol*. 1997;97(2):306-8.
35. Pawson R, Dyer MJ, Barge R, et al. Treatment of T-cell prolymphocytic leukemia with human CD52 antibody. *J Clin Oncol*. 1997;15(7):2667-72.
36. Newman DK, Isaacs JD, Watson PG, Meyer PA, Hale G, Waldmann H. Prevention of immune-mediated corneal graft destruction with the anti- lymphocyte monoclonal antibody, CAMPATH-1H. *Eye*. 1995;9((Pt 5)):564-9.
37. Friend PJ, Waldmann H, Hale G, et al. Reversal of allograft rejection using the monoclonal antibody, Campath- 1G. *Transplant Proc*. 1991;23(4):2253-4.
38. Calne R, Moffatt SD, Friend PJ, et al. Campath IH allows low-dose cyclosporine monotherapy in 31 cadaveric renal allograft recipients. *Transplantation*. 1999;68(10):1613-6.
39. Osterborg A, Dyer MJ, Bunjes D, et al. Phase II multicenter study of human CD52 antibody in previously treated chronic lymphocytic leukemia. European Study Group of CAMPATH-1H Treatment in Chronic Lymphocytic Leukemia. *J Clin Oncol*. 1997;15(4):1567-74.
40. Osterborg A, Fassas AS, Anagnostopoulos A, Dyer MJ, Catovsky D, Mellstedt H. Humanized CD52 monoclonal antibody Campath-1H as first-line treatment in chronic lymphocytic leukaemia. *Br J Haematol*. 1996;93(1):151-3.
41. Bowen AL, Zomas A, Emmett E, Matutes E, Dyer MJ, Catovsky D. Subcutaneous CAMPATH-1H in fludarabine-resistant/relapsed chronic lymphocytic and B-prolymphocytic leukaemia. *Br J Haematol*. 1997;96(3):617-9.
42. Kottaridis PD, Milligan DW, Chopra R, et al. In vivo CAMPATH-1H prevents graft-versus-host disease following nonmyeloablative stem cell transplantation. *Blood*. 2000;96(7):2419-25.
43. Hale G, Jacobs P, Wood L, et al. CD52 antibodies for prevention of graft-versus-host disease and graft rejection following transplantation of allogeneic peripheral blood stem cells. *Bone Marrow Transplant*. 2000;26(1):69-76.
44. Hale G, Waldmann H. Recent results using CAMPATH-1 antibodies to control GVHD and graft rejection. *Bone Marrow Transplant*. 1996;17(3):305-8.
45. Chakraverty R, Peggs K, Chopra R, et al. Limiting transplantation-related mortality following unrelated donor stem cell transplantation by using a nonmyeloablative conditioning regimen. *Blood*. 2002;99(3):1071-8.
46. Kanda Y, Oshima K, Asano-Mori Y, et al. In vivo alemtuzumab enables haploidentical human leukocyte antigen-mismatched hematopoietic stem-cell transplantation without ex vivo graft manipulation. *Transplantation*. May 27 2005;79(10):1351-7.
47. Malladi RK, Peniket AJ, Littlewood TJ, et al. Alemtuzumab markedly reduces chronic GVHD without

affecting overall survival in reduced-intensity conditioning sibling allo-SCT for adults with AML. *Bone Marrow Transplant*. May 2009;43(9):709-15.
48. Shah AJ, Kapoor N, Crooks GM, et al. The effects of Campath 1H upon graft-versus-host disease, infection, relapse, and immune reconstitution in recipients of pediatric unrelated transplants. *Biol Blood Marrow Transplant*. May 2007;13(5):584-93.
49. Siegal D, Xu W, Sutherland R, et al. Graft-versus-host disease following marrow transplantation for aplastic anemia: different impact of two GVHD prevention strategies. *Bone Marrow Transplant*. Jul 2008;42(1):51-6.
50. Powell JD, Lerner CG, Schwartz RH. Inhibition of cell cycle progression by rapamycin induces T cell clonal anergy even in the presence of costimulation. *J Immunol*. 1999;162(5):2775-84.
51. Hale DA, Gottschalk R, Fukuzaki T, Wood ML, Maki T, Monaco AP. Extended skin allo- and xenograft survival in mice treated with rapamycin, antilymphocyte serum, and donor-specific bone marrow transfusion. *Transplant Proc*. 1996;28(6):3269.
52. Hale DA, Gottschalk R, Fukuzaki T, Wood ML, Maki T, Monaco AP. Superiority of sirolimus (rapamycin) over cyclosporine in augmenting allograft and xenograft survival in mice treated with antilymphocyte serum and donor-specific bone marrow. *Transplantation*. 1997;63(3):359-64.
53. Hale DA, Gottschalk R, Umemura A, Maki T, Monaco AP. Establishment of stable multilineage hematopoietic chimerism and donor- specific tolerance without irradiation. *Transplantation*. 2000;69(7):1242-51.
54. Antin JH, Kim HT, Cutler C, et al. Sirolimus, tacrolimus, and low-dose methotrexate for graft-versus-host disease prophylaxis in mismatched related donor or unrelated donor transplantation. *Blood*. Sep 1 2003;102(5):1601-5.
55. Schleuning M, Judith D, Jedlickova Z, et al. Calcineurin inhibitor-free GVHD prophylaxis with sirolimus, mycophenolate mofetil and ATG in Allo-SCT for leukemia patients with high relapse risk: an observational cohort study. *Bone Marrow Transplant*. May 2009;43(9):717-23.
56. Kahan BD. Efficacy of sirolimus compared with azathioprine for reduction of acute renal allograft rejection: a randomised multicentre study. The Rapamune US Study Group. *Lancet*. 2000;356(9225):194-202.
57. Groth CG, Backman L, Morales JM, et al. Sirolimus (rapamycin)-based therapy in human renal transplantation: similar efficacy and different toxicity compared with cyclosporine. Sirolimus European Renal Transplant Study Group. *Transplantation*. 1999;67(7):1036-42.
58. O'Donnell PV, Luznik L, Jones RJ, et al. Nonmyeloablative bone marrow transplantation from partially HLA-mismatched related donors using posttransplantation cyclophosphamide. *Biol Blood Marrow Transplant*. 2002;8(7):377-86.
59. Luznik L, Jalla S, Engstrom LW, Iannone R, Fuchs EJ. Durable engraftment of major histocompatibility complex-incompatible cells after nonmyeloablative conditioning with fludarabine, low-dose total body irradiation, and posttransplantation cyclophosphamide. *Blood*. Dec 1 2001;98(12):3456-64.
60. Colson YL, Wren SM, Schuchert MJ, et al. A nonlethal conditioning approach to achieve durable multilineage mixed chimerism and tolerance across major, minor, and hematopoietic histocompatibility barriers. *J Immunol*. Nov 1 1995;155(9):4179-88.
61. Colson YL, Li H, Boggs SS, Patrene KD, Johnson PC, Ildstad ST. Durable mixed allogeneic chimerism and tolerance by a nonlethal radiation-based cytoreductive approach. *J Immunol*. Oct 1 1996;157(7):2820-9.
62. Brodsky RA, Luznik L, Bolanos-Meade J, Leffell MS, Jones RJ, Fuchs EJ. Reduced intensity HLA-haploidentical BMT with post transplantation cyclophosphamide in nonmalignant hematologic diseases. *Bone Marrow Transplant*. Oct 2008;42(8):523-7. doi:10.1038/bmt.2008.203
63. Bolanos-Meade J, Fuchs EJ, Luznik L, et al. HLA-haploidentical bone marrow transplantation with posttransplant cyclophosphamide expands the donor pool for patients with sickle cell disease. Clinical Trial, Phase I
Clinical Trial, Phase II
Research Support, N.I.H., Extramural. *Blood*. Nov 22 2012;120(22):4285-91. doi:10.1182/blood-2012-07-438408

64. Fitzhugh CD, Weitzel RP, Hsieh MM, et al. Sirolimus and post transplant Cy synergistically maintain mixed chimerism in a mismatched murine model. *Bone Marrow Transplant*. Oct 2013;48(10):1335-41. doi:10.1038/bmt.2013.60
65. Bubalo JS, Cherala G, McCune JS, Munar MY, Tse S, Maziarz R. Aprepitant pharmacokinetics and assessing the impact of aprepitant on cyclophosphamide metabolism in cancer patients undergoing hematopoietic stem cell transplantation. Randomized Controlled Trial
Research Support, Non-U.S. Gov't. *J Clin Pharmacol*. Apr 2012;52(4):586-94. doi:10.1177/0091270011398243
66. Joy MS, La M, Wang J, et al. Cyclophosphamide and 4-hydroxycyclophosphamide pharmacokinetics in patients with glomerulonephritis secondary to lupus and small vessel vasculitis. Controlled Clinical Trial
Research Support, N.I.H., Extramural
Research Support, N.I.H., Intramural
Research Support, Non-U.S. Gov't. *Br J Clin Pharmacol*. Sep 2012;74(3):445-55. doi:10.1111/j.1365-2125.2012.04223.x
67. Walko CM, Combest AJ, Spasojevic I, et al. The effect of aprepitant and race on the pharmacokinetics of cyclophosphamide in breast cancer patients. Randomized Controlled Trial
Research Support, N.I.H., Extramural
Research Support, Non-U.S. Gov't. *Cancer Chemother Pharmacol*. May 2012;69(5):1189-96. doi:10.1007/s00280-011-1815-5
68. Navid F, Baker SD, McCarville MB, et al. Phase I and clinical pharmacology study of bevacizumab, sorafenib, and low-dose cyclophosphamide in children and young adults with refractory/recurrent solid tumors. *Clinical cancer research : an official journal of the American Association for Cancer Research*. Jan 1 2013;19(1):236-46. doi:10.1158/1078-0432.CCR-12-1897
69. de Jonge ME, Huitema AD, Rodenhuis S, Beijnen JH. Clinical pharmacokinetics of cyclophosphamide. Research Support, Non-U.S. Gov't
Review. *Clin Pharmacokinet*. 2005;44(11):1135-64.
70. Pinto N, Ludeman SM, Dolan ME. Drug focus: Pharmacogenetic studies related to cyclophosphamide-based therapy. Research Support, N.I.H., Extramural
Research Support, Non-U.S. Gov't
Review. *Pharmacogenomics*. Dec 2009;10(12):1897-903. doi:10.2217/pgs.09.134
71. Haubitz M, Bohnenstengel F, Brunkhorst R, Schwab M, Hofmann U, Busse D. Cyclophosphamide pharmacokinetics and dose requirements in patients with renal insufficiency. Research Support, Non-U.S. Gov't. *Kidney Int*. Apr 2002;61(4):1495-501. doi:10.1046/j.1523-1755.2002.00279.x
72. Grever MR. Pentostatin: impact on outcome in hairy cell leukemia. *Hematol Oncol Clin North Am*. Oct 2006;20(5):1099-108. doi:S0889-8588(06)00108-0 [pii]
10.1016/j.hoc.2006.06.001
73. Ho AD, Suciu S, Stryckmans P, et al. Pentostatin in T-cell malignancies--a phase II trial of the EORTC. Leukemia Cooperative Group. *Ann Oncol*. Dec 1999;10(12):1493-8.
74. Kay NE, Geyer SM, Call TG, et al. Combination chemoimmunotherapy with pentostatin, cyclophosphamide, and rituximab shows significant clinical activity with low accompanying toxicity in previously untreated B chronic lymphocytic leukemia. *Blood*. Jan 15 2007;109(2):405-11. doi:blood-2006-07-033274 [pii]
10.1182/blood-2006-07-033274
75. Benveniste P, Cohen A. p53 expression is required for thymocyte apoptosis induced by adenosine deaminase deficiency. *Proc Natl Acad Sci U S A*. Aug 29 1995;92(18):8373-7.
76. Pavletic SZ, Bociek RG, Foran JM, et al. Lymphodepleting effects and safety of pentostatin for nonmyeloablative allogeneic stem-cell transplantation. *Transplantation*. Sep 15 2003;76(5):877-81. doi:10.1097/01.TP.0000084869.08639.A0
77. Miller KB, Roberts TF, Chan G, et al. A novel reduced intensity regimen for allogeneic hematopoietic stem cell transplantation associated with a reduced incidence of graft-versus-host disease. *Bone Marrow Transplant*. May 2004;33(9):881-9. doi:10.1038/sj.bmt.1704454

1704454 [pii]
78. Mariotti J, Taylor J, Massey PR, et al. The pentostatin plus cyclophosphamide nonmyeloablative regimen induces durable host T cell functional deficits and prevents murine marrow allograft rejection. *Biol Blood Marrow Transplant*. May 2011;17(5):620-31. doi:S1083-8791(10)00539-2 [pii]
10.1016/j.bbmt.2010.11.029
79. Mossoba ME, Onda M, Taylor J, et al. Pentostatin plus cyclophosphamide safely and effectively prevents immunotoxin immunogenicity in murine hosts. *Clin Cancer Res*. Jun 1 2011;17(11):3697-705. doi:1078-0432.CCR-11-0493 [pii]
10.1158/1078-0432.CCR-11-0493
80. Fitzhugh CD, Cordes S, Taylor T, et al. At Least 20% Donor Myeloid Chimerism is Necessary to Reverse the Sickle Phenotype after Allogeneic Hematopoietic Stem Cell Transplantation. *Blood*. 2017;
81. Bolanos-Meade J, Cooke KR, Gamper CJ, et al. Effect of increased dose of total body irradiation on graft failure associated with HLA-haploidentical transplantation in patients with severe haemoglobinopathies: a prospective clinical trial. *Lancet Haematol*. Apr 2019;6(4):e183-e193. doi:10.1016/S2352-3026(19)30031-6
82. de la Fuente J, Hanna R, Gamper C, et al. Augmenting Non-Myeloablative BMT with Ptcy Using Thiotepa or 400 Cgy TBI Improves Engraftment in Patients with Transfusion Dependent Thalassemia: Results of a Haploidentical Transplant Consortium for Hemoglobinopathies (ICHH). *Biology of Blood and Marrow Transplantation*. Mar 2019;25(3)
83. Ciurea SO, de Lima M, Cano P, et al. High risk of graft failure in patients with anti-HLA antibodies undergoing haploidentical stem-cell transplantation. *Transplantation*. Oct 27 2009;88(8):1019-24. doi:10.1097/TP.0b013e3181b9d710
84. Chang YJ, Zhao XY, Xu LP, et al. Donor-specific anti-human leukocyte antigen antibodies were associated with primary graft failure after unmanipulated haploidentical blood and marrow transplantation: a prospective study with randomly assigned training and validation sets. *J Hematol Oncol*. 2015;8:84. doi:10.1186/s13045-015-0182-9
85. Al Khawaja SA, Ateya ZM, Al Hammam RA. Predictors of mortality in adults with Sickle cell disease admitted to intensive care unit in Bahrain. *J Crit Care*. Dec 2017;42:238-242. doi:10.1016/j.jcrc.2017.07.032
86. DeBaun MR, Casella JF. Transfusions for silent cerebral infarcts in sickle cell anemia. Comment Letter. *The New England journal of medicine*. Nov 06 2014;371(19):1841-2. doi:10.1056/NEJMc1411133
87. Jordan LC, Kassim AA, Donahue MJ, et al. Silent infarct is a risk factor for infarct recurrence in adults with sickle cell anemia. *Neurology*. Aug 21 2018;91(8):e781-e784. doi:10.1212/WNL.0000000000006047
88. Fitzhugh CD, Hsieh MM, Bolan CD, Saenz C, Tisdale JF. Granulocyte colony-stimulating factor (G-CSF) administration in individuals with sickle cell disease: time for a moratorium? *Cytotherapy*. 2009;11(4):464-71. doi:10.1080/14653240902849788
89. Al-Khabori M, Al-Ghafri F, Al-Kindi S, et al. Safety of stem cell mobilization in donors with sickle cell trait. *Bone Marrow Transplant*. Feb 2015;50(2):310-1. doi:10.1038/bmt.2014.252
90. Lagresle-Peyrou C, Lefrere F, Magrin E, et al. Plerixafor enables safe, rapid, efficient mobilization of hematopoietic stem cells in sickle cell disease patients after exchange transfusion. *Haematologica*. May 2018;103(5):778-786. doi:10.3324/haematol.2017.184788
91. Esrick EB, Manis JP, Daley H, et al. Successful hematopoietic stem cell mobilization and apheresis collection using plerixafor alone in sickle cell patients. *Blood Adv*. Oct 9 2018;2(19):2505-2512. doi:10.1182/bloodadvances.2018016725
92. Boulad F, Shore T, van Besien K, et al. Safety and efficacy of plerixafor dose escalation for the mobilization of CD34(+) hematopoietic progenitor cells in patients with sickle cell disease: interim results. *Haematologica*. May 2018;103(5):770-777. doi:10.3324/haematol.2017.187047
93. Wielosz E, Dryglewska M, Majdan M. Antiphospholipid antibodies and kidney involvement in patients with systemic sclerosis. *Clin Rheumatol*. Aug 2009;28(8):955-9. doi:10.1007/s10067-009-1188-x
94. De Vlaminck I, Valantine HA, Snyder TM, et al. Circulating cell-free DNA enables noninvasive diagnosis of heart transplant rejection. Research Support, N.I.H., Extramural
Research Support, Non-U.S. Gov't. *Sci Transl Med*. Jun 18 2014;6(241):241ra77. doi:10.1126/scitranslmed.3007803

95. Poi MJ, Hofmeister CC, Johnston JS, et al. Standard pentostatin dose reductions in renal insufficiency are not adequate: selected patients with steroid-refractory acute graft-versus-host disease. *Clin Pharmacokinet*. Aug 2013;52(8):705-12. doi:10.1007/s40262-013-0064-7
96. Ciurea SO, Thall PF, Milton DR, et al. Complement-Binding Donor-Specific Anti-HLA Antibodies and Risk of Primary Graft Failure in Hematopoietic Stem Cell Transplantation. *Biol Blood Marrow Transplant*. Aug 2015;21(8):1392-8. doi:10.1016/j.bbmt.2015.05.001
97. Vorstrup S, Lass P, Waldemar G, et al. Increased cerebral blood flow in anemic patients on long-term hemodialytic treatment. *J Cereb Blood Flow Metab*. Sep 1992;12(5):745-9. doi:10.1038/jcbfm.1992.105
98. Prohovnik I, Hurlet-Jensen A, Adams R, De Vivo D, Pavlakis SG. Hemodynamic etiology of elevated flow velocity and stroke in sickle-cell disease. *J Cereb Blood Flow Metab*. Apr 2009;29(4):803-10. doi:10.1038/jcbfm.2009.6
99. Gevers S, Nederveen AJ, Fijnvandraat K, et al. Arterial spin labeling measurement of cerebral perfusion in children with sickle cell disease. *J Magn Reson Imaging*. Apr 2012;35(4):779-87. doi:10.1002/jmri.23505
100. Bush AM, Borzage MT, Choi S, et al. Determinants of resting cerebral blood flow in sickle cell disease. *Am J Hematol*. Sep 2016;91(9):912-7. doi:10.1002/ajh.24441
101. Juttukonda MR, Jordan LC, Gindville MC, et al. Cerebral hemodynamics and pseudo-continuous arterial spin labeling considerations in adults with sickle cell anemia. *NMR Biomed*. Feb 2017;30(2)doi:10.1002/nbm.3681
102. Juttukonda MR, Donahue MJ, Davis LT, et al. Preliminary evidence for cerebral capillary shunting in adults with sickle cell anemia. *J Cereb Blood Flow Metab*. Jun 2019;39(6):1099-1110. doi:10.1177/0271678X17746808
103. Hurlet-Jensen AM, Prohovnik I, Pavlakis SG, Piomelli S. Effects of total hemoglobin and hemoglobin S concentration on cerebral blood flow during transfusion therapy to prevent stroke in sickle cell disease. *Stroke*. Aug 1994;25(8):1688-92. doi:10.1161/01.str.25.8.1688
104. Arkuszewski M, Krejza J, Chen R, et al. Sickle cell anemia: intracranial stenosis and silent cerebral infarcts in children with low risk of stroke. *Adv Med Sci*. Mar 2014;59(1):108-13. doi:10.1016/j.advms.2013.09.001
105. Gupta A, Chazen JL, Hartman M, et al. Cerebrovascular reserve and stroke risk in patients with carotid stenosis or occlusion: a systematic review and meta-analysis. *Stroke*. Nov 2012;43(11):2884-91. doi:10.1161/STROKEAHA.112.663716
106. Derdeyn CP, Videen TO, Yundt KD, et al. Variability of cerebral blood volume and oxygen extraction: stages of cerebral haemodynamic impairment revisited. *Brain*. Mar 2002;125(Pt 3):595-607. doi:10.1093/brain/awf047
107. Jordan LC, Gindville MC, Scott AO, et al. Non-invasive imaging of oxygen extraction fraction in adults with sickle cell anaemia. *Brain*. Mar 2016;139(Pt 3):738-50. doi:10.1093/brain/awv397
108. Fields ME, Guilliams KP, Ragan DK, et al. Regional oxygen extraction predicts border zone vulnerability to stroke in sickle cell disease. *Neurology*. Mar 27 2018;90(13):e1134-e1142. doi:10.1212/WNL.0000000000005194
109. Stotesbury H, Hales PW, Kirkham FJ. The promise of noninvasive cerebral hemodynamic assessment in sickle cell anemia. *Neurology*. Mar 27 2018;90(13):585-586. doi:10.1212/WNL.0000000000005236
110. Jordan LC, Juttukonda MR, Kassim AA, et al. Haploidentical bone marrow transplantation improves cerebral hemodynamics in adults with sickle cell disease. *Am J Hematol*. Jun 2019;94(6):E155-E158. doi:10.1002/ajh.25455
111. Buchali A, Feyer P, Groll J, Massenkeil G, Arnold R, Budach V. Immediate toxicity during fractionated total body irradiation as conditioning for bone marrow transplantation. *Radiother Oncol*. 2000;54(2):157-62.
112. Deeg HJ. Acute and delayed toxicities of total body irradiation. Seattle Marrow Transplant Team. *Int J Radiat Oncol Biol Phys*. 1983;9(12):1933-9.
113. Thomas O, Mahe M, Campion L, et al. Long-term complications of total body irradiation in adults. *Int J Radiat Oncol Biol Phys*. 2001;49(1):125-31.

114. Socie G, Salooja N, Cohen A, et al. Nonmalignant late effects after allogeneic stem cell transplantation. *Blood*. May 1 2003;101(9):3373-85.
115. Sanders JE, Hawley J, Levy W, et al. Pregnancies following high-dose cyclophosphamide with or without high-dose busulfan or total-body irradiation and bone marrow transplantation. *Blood*. Apr 1 1996;87(7):3045-52.
116. Gerrard GE, Vail A, Taylor RE, et al. Toxicity and dosimetry of fractionated total body irradiation prior to allogeneic bone marrow transplantation using a straightforward radiotherapy technique. *Clin Oncol*. 1998;10(6):379-83.
117. Bhatia S, Louie AD, Bhatia R, et al. Solid cancers after bone marrow transplantation. *J Clin Oncol*. 2001;19(2):464-71.
118. Kolb HJ, Socie G, Duell T, et al. Malignant neoplasms in long-term survivors of bone marrow transplantation. Late Effects Working Party of the European Cooperative Group for Blood and Marrow Transplantation and the European Late Effect Project Group. *Ann Intern Med*. 1999;131(10):738-44.
119. Deeg HJ, Socie G, Schoch G, et al. Malignancies after marrow transplantation for aplastic anemia and fanconi anemia: a joint Seattle and Paris analysis of results in 700 patients. *Blood*. 1996;87(1):386-92.
120. Miller C, Miller M, McMullin B, et al. A phase I clinical study of inhaled nitric oxide in healthy adults. *J Cyst Fibros*. Jul 2012;11(4):324-31. doi:10.1016/j.jcf.2012.01.003
121. McLaughlin VV, Archer SL, Badesch DB, et al. ACCF/AHA 2009 expert consensus document on pulmonary hypertension a report of the American College of Cardiology Foundation Task Force on Expert Consensus Documents and the American Heart Association developed in collaboration with the American College of Chest Physicians; American Thoracic Society, Inc.; and the Pulmonary Hypertension Association. *J Am Coll Cardiol*. Apr 28 2009;53(17):1573-619. doi:10.1016/j.jacc.2009.01.004
122. Bonig H, Papayannopoulou T. Hematopoietic stem cell mobilization: updated conceptual renditions. *Leukemia*. Jan 2013;27(1):24-31. doi:10.1038/leu.2012.254
123. Liles WC, Broxmeyer HE, Rodger E, et al. Mobilization of hematopoietic progenitor cells in healthy volunteers by AMD3100, a CXCR4 antagonist. *Blood*. October 15, 2003 2003;102(8):2728-2730.
124. Hendrix CW, Collier AC, Lederman MM, et al. Safety, pharmacokinetics, and antiviral activity of AMD3100, a selective CXCR4 receptor inhibitor, in HIV-1 infection. *J Acquir Immune Defic Syndr*. Oct 1 2004;37(2):1253-62.
125. Bendall LJ, Bradstock KF. G-CSF: From granulopoietic stimulant to bone marrow stem cell mobilizing agent. *Cytokine Growth Factor Rev*. Aug 2014;25(4):355-67. doi:10.1016/j.cytogfr.2014.07.011
126. Devine SM, Vij R, Rettig M, et al. Rapid mobilization of functional donor hematopoietic cells without G-CSF using AMD3100, an antagonist of the CXCR4/SDF-1 interaction. *Blood*. Aug 15 2008;112(4):990-8. doi:10.1182/blood-2007-12-130179
127. Pantin J, Purev E, Tian X, et al. Effect of high-dose plerixafor on CD34(+) cell mobilization in healthy stem cell donors: results of a randomized crossover trial. *Haematologica*. Mar 2017;102(3):600-609. doi:10.3324/haematol.2016.147132
128. Simon R. Optimal two-stage designs for phase II clinical trials. *Control Clin Trials*. Mar 1989;10(1):1-10.
129. Jung SH, Lee T, Kim K, George SL. Admissible two-stage designs for phase II cancer clinical trials. *Stat Med*. Feb 28 2004;23(4):561-9. doi:10.1002/sim.1600
130. Teira P, Battiwalla M, Ramanathan M, et al. Early cytomegalovirus reactivation remains associated with increased transplant-related mortality in the current era: a CIBMTR analysis. *Blood*. May 19 2016;127(20):2427-38. doi:10.1182/blood-2015-11-679639
131. Uhlin M, Wikell H, Sundin M, et al. Risk factors for Epstein-Barr virus-related post-transplant lymphoproliferative disease after allogeneic hematopoietic stem cell transplantation. *Haematologica*. Feb 2014;99(2):346-52. doi:10.3324/haematol.2013.087338
132. Higman MA, Vogelsang GB. Chronic graft versus host disease. *Br J Haematol*. May 2004;125(4):435-54. doi:10.1111/j.1365-2141.2004.04945.x
133. Storb R, Prentice RL, Sullivan KM, et al. Predictive factors in chronic graft-versus-host disease in patients with aplastic anemia treated by marrow transplantation from HLA-identical siblings. *Ann Intern Med*.

Apr 1983;98(4):461-6.

APPENDIX A: ECOG PERFORMANCE STATUS SCALE

GRADE	DESCRIPTION
0	Fully active, able to carry on all pre-disease activities withoutrestriction.
1	Restricted in physically strenuous activities and able to carry out work of a light orsedentary nature, e.g. light housework, office work.
2	Ambulatory and capable of all self-care but unable to carry out any work activities. Up and about more than 50% of waking hours.
3	Capable of only limited self-care; confined to bed or chair more than 50% of waking hours.
4	Completely disabled. Cannot carry on any self-care. Totally confined to bed orchair.
5	Dead.

APPENDIX B: TRANSFUSION OF RED CELLS TO RECIPIENTS OF ABO INCOMPATIBLE MARROW

Major ABO incompatible recipient - donor

Patient	Donor	Transfused RBC = patients group
O	A, B or AB	O
A	B or AB	A or O
B	A or AB	B or O

Minor ABO incompatibility recipient - donor

Patient	Donor	Transfused RBC = donor group
A, B or AB	O	O
AB	B	B or O
AB	A	A or O

TRANSFUSION OF PLATELETS TO RECIPIENTS OF ABO INCOMPATIBLE MARROW

Give donor group or volume reduce
Additional pre- and post-transplant monitoring will be performed to monitor donor erythropoiesis and immune hemolysis.

APPENDIX C: TRANSFUSION MEDICINE GUIDELINES FOR APHERESIS PROCEDURES IN PBSC DONORS AND SICKLE CELL PATIENTS RECEIVING NONMYELOABLATIVE ALLOGENEIC PBSC TRANSPLANTS

Introduction

Sickle cell disease (SCD) patients are at risk for vaso-occlusive crises such as cerebrovascular accidents and the acute chest syndrome which occur due to the viscosity and sickling properties of sickle hemoglobin S. Pre- and peri-transplant maneuvers, such as transfusion or red cell exchanges with allogeneic red cells, may be performed to reduce the risk of ischemic events by reducing the levels of hemoglobin S. Pre-transplant coordination with the Department of Transfusion Medicine (DTM) is critical because these patients may have had prior transfusions and have developed alloantibodies. In addition, the distribution of red blood cell phenotypes in patients with SCD will reflect their ethnic heritage and may differ from that in the NIH donor pool. Recruitment of adequate numbers of compatible units thus requires careful advance planning and knowledge of the patient's phenotype and antibody screen.

Allogeneic transplant patients who receive a lymphocyte-replete PBSC graft will also be at risk for increased red cell requirements if there is an ABO incompatibility with the donor. Minor ABO incompatibility, such as O donors into A, B or AB recipients (see Appendix B), is associated with hemolysis due to production of anti-recipient isohemagglutinins by passenger lymphocytes. Major ABO incompatibility, such as A or B donors into O patients, may be associated with a delayed onset of effective donor erythropoiesis resulting in pure red cell aplasia after conversion to full donor hematopoiesis. This event appears to be most common after non-myeloablative conditioning regimens that are permissive for persistent production of anti-donor, host-type isohemagglutinins.

These patients may have a further increased red cell requirement and need for advanced planning and recruitment. Patients with major ABO incompatibility will have their peripheral blood stem cells plasma-reduced and red cell-reduced as needed per DTM guidelines in consultation with the PI/Lead Associate Investigator. Regarding patients with minor ABO incompatibility, immediate complications at the time of infusion can be managed by standard DTM policies for manipulation of the graft to remove plasma (see Appendix C). The appropriate transfusion policy in the peri-transplant period for the ABO group of red cell, plasma and platelet transfusions in patients with ABO incompatible donors is also managed according to standard DTM transplant policies. In addition, the DTM will use red cell serologic testing to carefully monitor those patients who have minor ABO incompatibility with the donor for evidence of hemolysis in the peri-transplant period using a standard operating procedure.

A potential adverse event in hematopoietic transplantation for SCD is rejection of the PBSC graft, especially in patients who have been heavily transfused. Obtaining higher numbers of donor stem cells may reduce the risk of graft rejection. To achieve this goal, the DTM will collect stem cells using a single very large volume apheresis (~4-5 donor blood volumes) on day 5 after filgrastim (G-CSF) administration, which reduces the incidence of thrombocytopenia in the donor associated with apheresis, reduces apheresis time and the time with which central venous catheters remain in place, and produces the same yields as two smaller donor blood volume procedures performed consecutively on days 5 and 6.

All sickle cell transplant candidates and their identified donors will need to have a full red cell phenotype, antibody screen, and quantitative hemoglobin electrophoresis obtained during initial evaluation, well in advance of any apheresis procedures. The DTM will enter appropriate restrictions for blood product transfusion based on this information.

Procedures for Red Cell Exchanges

Sickle cell patients who are not receiving long term transfusion therapy will be evaluated by the DTM fellow/senior staff and considered for a prophylactic red cell exchange prior to transplant for a target HbS <30% to reduce the incidence of post-transplant stroke and other events that may be associated with high hemoglobin S levels. ADSOL leukoreduced packed red cells will be used for the exchange.

These patients will have a hemoglobin electrophoresis performed to determine their initial fraction of hemoglobin S (% HbS). They must also have a full type and screen performed to identify alloantibodies and allow for recruitment of donors prior to the exchange. The patient total blood volume will be computed from an algorithm using the COBE computer, and the volume of replacement PRBC required for the exchange estimated by utilizing this computer in conjunction with the initial HbS content and the desired end hematocrit and HbS concentration. For this protocol, the calculation of the COBE computer may be verified using the following calculations for determination of the volume of replacement PRBCs needed for the red cell exchange.

Blood volume x patient hct = Patient's Total Packed Red Cell Volume (PRCV) (PRCV) x
% HbS = Patient Total Packed RBC Volume of HbS (PRCV-S)
The volume of ADSOL PRBC needed to bring the residual fraction of red cells to 30% is 1.25 exchange volumes. (= 1.25 x (PRCV-S))

Since increasing the hematocrit in patients with high levels of HbS may precipitate vaso-occlusive crises, the red cell exchange will replace the red cells that are removed with an equal volume of infused red cells. Patients who are significantly anemic may have further transfusions given after the exchange to bring their final hematocrit up to 35%. In these cases, the target % HbS should be 30% after the final transfusions bring the hematocrit up to 35%.
The target for the % HbS after the exchange (before additional transfusions) is 35/Hct x 30%, where Hct is the patient hematocrit before the exchange. After the exchange the patient will then receive a volume of red cells equal to approximately (0.35-Hct)(wt)(70 ml/kg).

Patients undergoing red cell exchange may experience citrate toxicity from the anticoagulant used in the apheresis procedure and contained in the ADSOL red cells. A citrate infusion rate will be calculated by the DTM fellow/senior staff based on the flow rate of returned red cells plus 2/3 of the citrate infusion rate. Patients who receive more than 1.2 mg of citrate per kilogram per minute will receive intravenous calcium through the return line at a rate of 0.5 mg of calcium ion per 21 mg of citrate.

Donor apheresis procedures
(Completed as a standard of care procedure on protocol 20-H-0099)

Donor stem cell mobilization with filgrastim (G-CSF)

After medical evaluation and clearance for suitability as an allogeneic donor, each donor will undergo mobilization with G-CSF, usually as an outpatient. The G-CSF will be administered in a dose of 10 to 16 ug/kg/day for 6-7 days, subcutaneously. The doses for days 1-4 may be given at any time of day, but the doses for day 5 and if necessary, day 6 must be given early in the morning, at least one hour prior to starting apheresis. Predictable side effects of G-CSF, including headache, bone pain, and myalgia, will be treated with analgesics as necessary. Prophylactic treatment of these side effects with the same medications may be elected. Other side effects will be evaluated and treated accordingly. The use of other PBSC mobilizing agents i.e. plerixafor will be determined per DTM SOPs in consultation with the PI.

Donor stem cell collection

The target CD34 dose is 10 x 10^6/kg, and the minimum is 5 x 10^6/kg. Donors will receive calcium chloride prophylaxis to prevent citrate toxicity in accordance with standard DTM policies.

The volume processed per apheresis procedure will be determined by DTM medical staff on the day of apheresis, based on peak CD34 cell mobilization response to filgrastim (G-CSF) and the CD34 cell dose needed, based on kilogram weight of recipient. This will range from 15 to 35 liters processed per day for 1 to 3 days, not to exceed a total of 75 liters over 3 days. In pediatric subjects, defined as less than 40 kg, a maximum of 8 blood volumes will be processed per day for up to 1-3 days.

The goal is to provide a sufficient number of CD34 cells to ensure engraftment and test the efficacy of this modality against disease relapse.

Filgrastim (G-CSF) administration.

G-CSF will be administered according to a vial-based algorithm to reduce wastage, improve patient compliance, and increase the total G-CSF dose to lighter weight donors in order to improve CD34 yields.

Donor Weight	Total filgrastim (G-CSF) Dose (range)
38-48 kg	600 mcg (12.5 to 15.8 mcg/kg)
49-56 kg	780 mcg (13.9 to 15.9 mcg/kg)
57-60 kg	900 mcg (15.0 to 15.8 mcg/kg)
61-67 kg	960 mcg (14.3 to 15.7 mcg/kg)
68-108 kg	1080 mcg (10.0 to 15.9 mcg/kg)
> 109 kg	1200 mcg (11.0 or less)

Ex vivo processing of PBPC and lymphocytes

The target cell doses for the PBPC graft are outlined in the section above.

For this protocol, there will be no T cell depletion of the PBPC or bone marrow. The PBPC and lymphocyte products will be cryopreserved in 5% DMSO/pentastarch for later thawing and infusion. In cases of RBC incompatibility, product manipulations will be done prior to cryopreservation. For minor ABO or other red cell incompatibility, PBPC products will undergo plasma removal, with resuspension in an infusible isotonic solution, according to standard operating procedures in the DTM Cell Processing Laboratory.

All products will be prepared for infusion by standard operating procedures of the DTM Cell Processing Laboratory.
All products will be prepared for infusion by SOPs of the DTM Cell Processing Laboratory.

APPENDIX D: AUTOLOGOUS PERIPHERAL BLOOD STEM CELL COLLECTION
(Completed after patient is signed onto this protocol)

Hydroxyurea will be held at least 2 weeks prior to plerixafor administration.

Prophylactic red blood cell exchange or simple red cell transfusions will be given to patients prior to plerixafor administration targeting HbS<30% to reduce the incidence of vaso-occlusive crises and other events that may be associated with high HbS levels.

Patients undergoing HSC mobilization will receive a single-dose subcutaneous administration of plerixafor (Mozobil®) at 240 µg/kg. If the CD34+ cell yields are not sufficient for a minimal target dose in each protocol, a second plerixafor administration can be performed at the same dose for mobilization and apheresis the following day. If the CD34+ cell counts are still not sufficient after a second day of collection then subsequent collections can be completed with a minimum of 2-3 weeks between collections to allow for recovery.

Leukapheresis will start approximately 4-12 hours after plerixafor is given. Mononuclear cells will be collected using an automated cell separator or apheresis device. Whole blood is withdrawn from one venipuncture site at a rate of 60-80 mL/min and then conveyed to the separator. If IV access is inadequate, central lines will be inserted prior to apheresis. Leukapheresis will be performed by the Apheresis Unit under supervision of the medical staff of the Department of Transfusion Medicine at the NIH Clinical Center

After successful blood withdrawal, blood is separated into cells and plasma by centrifugation. The light density mononuclear cells are collected into a component bag, and the remaining cells and plasma are re-infused into the patient via a second venipuncture site. Acid Citrate Dextrose formula A (ACD-A) is used as the anticoagulant at a whole blood to anticoagulant ratio of 13:1. Prophylactic intravenous calcium infusions are used in all procedures. Maximal extracorporeal blood volume during the procedure ranges from 300-400 mL.-Sufficient volumes of blood will be processed to obtain target CD34+ cells of 2.0x10e6 CD34+ cells/kg (minimum of 1.5x10e6 cells/kg).

The harvested peripheral blood apheresis products will be cryopreserved using a controlled rate freezer.

After successful mobilization and stem cell collection, patients will remain in the hospital for 1-3 days to monitor for side effects. The total inpatient duration is about 1 week. 3-10 days after discharge from hospital, patients will be evaluated in outpatient clinic. Laboratory and other testing will be obtained as clinically indicated.

APPENDIX E: SAMPLE CALENDAR

Sun	Mon	Tue	Wed	Thu	Fri	Sat
						1
2	*3*	*4*	*5*	*6 Day –21*<br>*Pentostatin Infusion*<br>*Oral cyclophospha-mide dose*	*7 Day –20*<br>*Oral cyclophosphamide dose*	*8 Day –19*<br>*Oral cyclophosphamide dose -*
9 Day –18<br>*Oral cyclophospha-*<br>*mide dose*	*10 Day –17*<br>*Pentostatin Infusion*<br>*Oral cyclophospha-*<br>*mide dose*	*11 Day –16*<br>*Oral cyclophospha-*<br>*mide dose*	*12 Day –15*<br>*Oral cyclophospha-*<br>*mide dose*	*13 Day –14*	*14 Day –13*<br>*Pentostatin Infusion*<br>*Oral cyclophosphamide dose*	*15 D-12*<br>*Oral cyclophosphamide dose-*
16 Day –11<br>*Oral cyclophospha-*<br>*mide dose*	*17 Day –10*<br>*Oral cyclophospha-mide dose*	*18 Day –9*<br>*Pentostatin Infusion*<br>*Oral cyclophospha-mide dose*	*19 Day –8*<br>*Oral cyclophospha-mide dose*	*20 Day –7*<br>*Campath Infusion*	*21 Day –6*<br>*Campath Infusion*	*22 Day –5*<br>*Campath Infusion*
23 Day –4<br>*Campath Infusion*	*24 Day –3*<br>*Campath Infusion*	*25 Day –2*	*26 Day –1*<br>*Radiation*	*27 Day 0*<br>***Stem Cell Infusion***	*28 Day +1*	*29 Day +2*
30 Day +3<br>*Cyclophosphamide Infusion*	*31 Day +4*<br>*Sirolimus starts*					

APPENDIX F: REPRODUCTIVE QUESTIONNAIRES IN PATIENTS WITH SEVERE CONGENITAL ANEMIAS BEFORE AND AFTER TRANSPLANT

Objectives

To obtain detailed medical, surgical, medication, social, family, and other relevant history, through the use of questionnaires, to complement endocrine related laboratory testing before and after hematopoietic stem cell transplant (HSCT)

To accumulate possible qualitative changes before and after stem cell transplant in the hypothalamic, pituitary, adrenal, and gonadal organs, with the emphasis on reproductive potential.

To estimate the magnitude and need of fertility related issues among transplanted patients.

Background and Scientific Rationale

Male and female patients with sickle cell disease (SCD) or thalassemia frequently suffer from endocrine and reproductive health problems, which can have multiple and complex etiologies. The underlying cause(s) can not only be related to the diseases themselves, but are frequently also a consequence of the treatment(s), which the patients have received in the past or are receiving currently. This area is under- studied in the literature; searching for "sickle cell" and "fertility" yielded only 87 entries in PubMed with 19 relevant reports. Searching for "sickle cell" and "reproduction" yielded 1464 entries, spanning 1960's to 2013. 258 entries were about prenatal or newborn screening, 297 were about pregnancy management or pregnancy outcomes, 29 were about contraception, and only 39 entries were relevant to the reproductive potential in sickle cell population. Thus these reports (19+39) are limited to address the causes of infertility (male or female factors), the effects of hydroxyurea, blood transfusion (and iron overload), iron chelators on gonadal function, or what magnitude and effects does chronic vaso-occlusion have on gonadal reserve. Half of the combined 58 articles discussed priapism, but only on medication or surgical management[1-3]– not on fertility potential. There were only case reports of fertility or fertility rates in women[4,5], but again are limited in addressing the causes of fertility potential with SCD and/or treatment[6].

Additionally, the literature about effects of hydroxyurea on fertility in male patients with SCD is mixed. Some report irreversible reduction in sperm production even with hydroxyurea discontinuation [7,8], while others report modest recovery[9]. There are no reports on the effects of hydroxyurea on gonadal function in women with SCD.

Currently HSCT is the only cure for patients with SCD and thalassemia. The most common type of HSCT employs full or myeloablative conditioning, which renders 60-85% of all transplant recipients infertile [10-12]. Reduced intensity or non-myeloablative regimens such as the one on this protocol theoretically should be less toxic on gonadal function. Different drug combinations and varying dosing schedule make the results from other transplant reports difficult to apply to this study[13,14]. Furthermore, our regimen uses total body irradiation, where testes are partially shielded but not ovaries, and alemtuzumab which targets CD52 on lymphocytes and spermatocytes[15]. These are unique features that make studying post-transplant endocrine effects (fertility potential) especially important. Our protocol also employs cyclophosphamide and pentostatin, so the results of this questionnaire could be compared to our patients who undergo HLA-matched sibling HSCT and don't receive cyclophosphamide and pentostatin. Those patients are already completing this questionnaire per protocols 03-H-0170 and 14-H- 0077).

When we previously approached the reproductive endocrinology group in NICHD about how best to follow our patients, they offered to evaluate our patients on as needed basis and pointed out their limitation – namely only for female patients as they are medically and surgically trained as obstetricians and gynecologists. There is currently no andrology support at this time. In order to determine whether the measures we initiate in association with HSCT contribute to negatively impact the patients' endocrine health in general and their reproductive health in particular, we propose to start with a questionnaire, designed for

male and female patients, which complements the endocrine related laboratory testing we are currently performing in this protocol (sections 10.1.22 and 10.4). Our interest is focused on determining whether our treatment (HSCT and associated medications) worsen the patients' endocrine balance, and if yes, to what extent. By ruling out other potential confounding factors, we will be able to better understand the role that our treatment may play.

Methods

There are several publicly available fertility related questionnaires (see web links after references). They include questions that are standard in the reproductive science field, as they address the patients' family, social, medical, and surgical histories, their medications, and prior treatments. With regard to the social history part of the questionnaire, a patient's lifestyle may provide clues or suggest any endocrine or reproductive health problems, as would be the case e.g. if patients were exposed to hazardous agents at work, or if they used certain recreational drugs.

There are no questionnaires that are specific for SCD or any other disorder; all the questionnaires are general – one for all patients. Thus, we reviewed all the available questionnaires, took all the standard questions, and added relevant questions for SCD and hemoglobinopathies. Our implementation method includes the following:
The questionnaire is voluntary. Subjects 18 years old and older are our intended focus: male questionnaire (3 pages), female questionnaires (3 pages).
For prospective patients, we plan to administer the questionnaire pre-HSCT, 1 year post-HSCT, and 2 year post-HSCT. The remaining 38 subjects (44 accrual ceiling – 6 transplanted subjects) should allow us to identify if there are any qualitative changes.
For patients who have already been transplanted, we also plan to administer the questionnaire annually for 2 times with the annual follow-up visits (for example, year 3 and 4 or year 6 and 7 depending on the patient). The answers from these patients would help to estimate the need of fertility related issues post-transplant.
The questionnaires are to be administered by the protocol providers or research coordinators with the patients to ensure uniformity. The questionnaires are not meant be filled out by the patients alone.
While it is common to obtain detailed history from partners of patients, we are omitting this aspect for several reasons. This effort is a pilot study with fact finding intent, thus we would like to focus on the patients first. Many of our patients currently do not have a stable partner to consider family planning, and we want to avoid re-administering the questionnaire if the partner were to change. Partner consenting to this addendum, possible impact of participating in questionnaire on the ongoing relationship, and confidentiality also make administering questionnaires to partners impractical for our team at this time.

Potential outcomes/results and data collection/analysis

On our HLA-matched sibling HSCT protocol, 2 male subjects reported decreased libido and erections in the first 6 months after transplant. As a result, we have sent them to for endocrine evaluation and related lab testing. We also had 1 female subject who has been trying to get

pregnant, and we have sent her to see medical and reproductive endocrinology for evaluation. Since often these patients have many transplant or medical issues, fertility related history is often neglected. This questionnaire helps to create a more complete set of data of fertility related issues/symptoms before and after transplant.

As we have done for the 3 patients listed in 4.1 of this section, when we identify area(s) of need in the patients in medical or reproductive endocrinology, we will inform the patients and refer them for suitable consultation either here at the Clinical Center or near patients' home residence, depending on the patients' input. If social work, psychologic, and other types of counseling is needed, we will find the most suitable consultation and/or referral.

Responses for men and women will be cataloged separately and proportion of patients with certain SCD treatment (e.g. how many red cell transfusions), symptoms (e.g. priapism or oligomenorrhea), or previous medical illness (e.g. infections) will be tabulated.

4.3.1.Responses from prospective patients will estimate the magnitude of fertility related needs or patterns before and after transplant, how/if they change after transplant short and long term.

4.3.2.Responses from already transplanted patients will add to the estimate of fertility need after transplant, and make the long term data immediately available.

Much of the data will be descriptive. The history portions of the questionnaires are intended to be compared qualitatively, and likely will be analyzed non-parametrically, similarly to this report[16].

When possible we will try to correlate the history portions with the laboratory testing, focusing on the reproductive aspect of the hypothalamic-pituitary-adrenal-reproductive axes. Some examples include does lower libido correlate with lower sex hormone levels, what are FSH and LH levels in women with irregular menses and how do they change over time?

References:

Bivalacqua TJ, Musicki B, Kutlu O, Burnett AL. New Insights into the pathophysiology of sickle cell disease-associated priapism. *The journal of sexual medicine.* 2012;9(1):79-87.

Broderick GA. Priapism and sickle-cell anemia: diagnosis and nonsurgical therapy. *The journal of sexual medicine.* 2012;9(1):88-103.

Burnett AL,Bivalacqua TJ, Champoin HC, Musicki B. Long-term oral phosphodiesterase 5 inhibitor therapy alleviates recurrent priapism. *Urology*. 2006;67(5):1043-1048.

Ballas SK, McCarthy WF, Guo N, et al. Exposure to hydroxyurea and pregnancy outcomes in patients with sickle cell anemia. *J Natl Med Assoc.* 2009;101(10):1046-1051.

Italia KY, Jijina FF, Chandrakala S, et al. Exposure to hydroxyurea during pregnancy in sickle-beta thalassemia: a report of 2 cases. *Journal of clinical pharmacology.* 2010;50(2):231-234.

Dovey S. Krishnamurti L, Sanfilippo J, et al. Oocyte cryopreservation in a patient with sickle cell disease prior to hematopoietic stem cell transplantation: first report. *Journal of assisted reproduction and genetics.* 2012;29(3):265-269.

Jones KM, Niaz MS, Brooks CM, et al. Adverse effects of a clinically relevant dose of hydroxyurea used for the treatment of sickle cell disease on male fertility endpoints. *International journal of environmental research and public health*. 2009;6(3):1124-1144.

Lukusa AK,Vermylen C, Vanabelle B, et al. Bone marrow transplantation or hydroxyurea for sickle cell anemia: long-term effects on semen variables and hormone profiles. *Pediatric hematology and oncology.* 2009;2(4):186-194.

Berthaut I, Guignedoux G, Kirsch-Noir F, et al. Influence of sickle cell disease and treatment with hydroxyurea on sperm parameters and fertility of human males. *Haematologica.* 2008;93(7):988- 993.

Thibaud E, Rodriguez-Macias K, Trivin C, Esperou H, Michon J, Brauner R. Ovarian function after bone marrow transplantation during childhood. *Bone marrow transplantation.* 1998;21(3):287-290.

Matsumoto M, Shinohara O, Ishiguro H, et al. Ovarian function after bone marrow transplantation performed before menarche. *Archives of disease in childhood.* 1999;80(5):452-454.

Sanders JE, Hawley J, Levy W, et al. Pregnancies following high-dose cyclophosphamide with or without high-dose busulfan or total-body irradiation and bone marrow transplantation. *Blood.* 1996;87(7):3045-3052.

Walters MC, Hardy K, Edwards S, et al. Pulmonary, gonadal, and central nervous system status after bone marrow transplantation for sickle cell disease. *Biology of blood and marrow transplantation: journal of the American Society for Blood and Marrow Transplantation.* 2010:16(2):263-272.

Vlachopapadopoulou E, Kitra V, Peristeri J, et al. Gonadal function of young patients with beta-thalassemia following bone marrow transplantation. *Journal of pediatric endocrinology & metapolism: JPEM.* 2005;18(5):477-483.

Focarelli R, Francavilla S, Francavilla F, Della Giovampaola C, Santucci A, Rosati F. A sialoglycoprotein, gp20, of the human capacitated sperm surface is a homologue of the leukocyte CD52 antigen: analysis of the effect of anti-cd52 monoclonal antibody (CAMPATH-1) on capacitated spermatozoa. *Molecular human reproduction.* 1999;5(1):46-51.

Gallo AM, Wilkie D, Suarez M, et al. Reproductive decisions in people with sickle cell disease or sickle cell trait. *Western journal of nursing research.* 2010;32(8):1073-1090.

Web links to publicly available fertility questionnaires
http://www.cdc.gov/reproductivehealth/Global/PDFs/RHAToolkitQuestionnaireUpdatedSeptember2011_FINAL_Tag508.pdf
http://shirenaturalfertility.com.au/wp-content/uploads/2011/06/First-Visit-Form.pdf
http://coe.ucsf.edu/ivf/forms/FORM_411-Male_Reproductive_Health_Questionnaire.pdf
http://urology2008-2012.ucsf.edu/patientGuides/pdf/maleInf/MaleIntake.pdf
http://www.racha.org.kh/rc2008/105/RPH-024-Eng.pdf
http://www.utswmedicine.org/hospitals-clinics/pob-2/ob-gyn/reproductive-health-questionnaire.pdf
www2.massgeneral.org/Vincent/images/ivf_infertilityquestionnaire1.pdf
infertility.wustl.edu/wp-content/uploads/2013/07/Medical-History-Questionnaire.pdf
www.uwmedicine.org/patient-care/our-services/medical-services/gynecology/documents/male- infertility-history.pdf
Obgyn.ucla.edu/workfiles/new_patients/patient_history_obgyn.pdf
www.uamshealth.com/upload/docs/clinics/women%20health/fertility-history-uams.pdf
www.shadygrovefertility.com/your_first_visit

APPENDIX G

DONOR SCHEDULE OF EVENTS

Procedure (X)= if clinically indicated	Pre-Apheresis		Post-Apheresis
	Baseline Testing	Once eligibility is confirmed	
Consent Form for Donor Screening (97-H-0041 or 20-H-0099)	X		
Consent Form for 20-H-0099 (standard of care GCSF & Apheresis) & 17-H-0069 (research samples)		X	
Procedures/Consults			
Baseline: Fit to donate history & physical exam with member of alternate team (non-transplant team) & Post Apheresis: Follow up with the member of primary transplant team)	X		X
Vital Signs, Height (pre-apheresis)/Weight	X		X
Venous Assessment (by apheresis team)	X		
DTM donor assessment consult (email DTM fellows)	X		
EKG	X		
Chest X ray	(X)		
Follow up Visit (within 2 weeks of starting GCSF)			X
GCSF (5 doses (6 if requires 2nd day))		X	
HLA Typing			
HLA typing: HLA- A, -B, -Cw, DR, DQ (low resolution) as many family members as possible	X		
HLA confirmatory A, B, C, DRB1 (confirm haploidentity of donor with patient)	X		
HLA Antibody Screen (Class 1, Class 2, DR, DQ)	X		
HLA High resolution (NGS) DR, DQ	X		
Sequence Based HLA A, B, C	X		
KIR Genotyping		X	
Clinical Labs			
CBC with Differential	X		X
Hgb electrophoresis	X		
Retic count	X		
Coagulation Screen	X		
STR Profile	X		
Type and Antibody Screen, Direct Antiglobulin Serum, Isohemagluttin Titers	X		
Extended Red Blood Cell Phenotyping	X		
Complete metabolic panel: (Acute care panel, hepatic panel, mineral panel)	X		X
Total protein, Creatinine kinase, LDH, Uric acid	X		
Iron and Transferrin	X		
β-HCG (Serum) (females only)	X		
HBV (Anti-Hbc-IgM, Anti-HBs antibody, HBs Ag, Anti-HBc antibody) & HIV-1/HCV/HBV NAT	X		
Malaria (smear & PCR) as needed based on travel hx.	(X)		
Toxoplasma (IgG & IgM), Anti Varicella IgG, CMV Antibodies (IgG, IgM) & Anti EBV Panel (no PCR)	X		
HCV, HIV (anti-HIV 1/2) & Anti-HTLV-I/II	X		
Anti-HAV antibody total & Anti HAV IgM	X		
Syphilis, Anti-HSV Type 1/2 (IgG/IgM), Babesia	X		
West Nile & T.Cruzi	X		
PROVIDER ADVERSE EVENT FORM			
AI to complete post-GSCF PBSC collection provider assessment form			X
RESEARCH SAMPLES			
Markers of Graft Rejection		X	
Cytokines		X	
Cytokine/Lymphocyte Function [included in the same sample for graft rejection markers]		X	
Functional Fluidics Sample (possibly depending on current # of patients)		X	
Eaton Studies (possibly depending on current # of patients)		X	
Stem Cells from collection		X	

RECIPIENT SCHEDULE OF EVENTS D+30 to 5 Years

**(X) = as clinically indicated**<br><br>**Procedure**	**D+30**	**Follow Up to D+100 (outpatient) (at least every 2-3 weeks up to D+100)**	**D+42 (6wks)**	**D+60 (2 mos)**	**D+90 (3 mos)**	**D+100**	**6 months[j]**	**12 mo/1yr[j]**	**18 mo/1.5yr[j]**	**24 mo/2yr[j]**	**36mo/3yr[j]**	**48 mo/4yr[j]**	**60mo/5yr[j]**	**Beyond 5 Yr***
**LAB TESTS**														
**CBC with differential**		X					X	X	X	X	X	X	X	X
**Reticulocyte count[d]**	X													
**Hgb electrophoresis**	X	X	X	X		X[i]	X	X	X	X	X	X	X	X
**Acute care panel, hepatic panel, mineral panel, creatinine kinase, LDH, total protein, uric acid, cystatin-C**		X	X				X	X	X	X	X	X	X	X
**Coagulation Screen[a, d]**		X	X											
**Sirolimus Levels [d]**	X		X	X	X	X	X	X	X	X	X	X	X	X
**d-dimer**						X[i]	X	X	X	X	X	X	X	X
**Pro-BNP [m]**						X	X	X	X	X	X	X	X	X
**Complete Lipid Profile & Triglycerides, Cholesterol[b]**	X[i]			X[i]	X[i]	X[i]	X	X	X	X	X	X	X	X
**Lipoprotein Profile & Apolipoprotein B[b]**	X			X[i]		X[i]	X	X	X	X	X	X	X	X
**Pre-albumin[d]**	X													
**Troponin T or I**						X[i]								
**Endocrine Testing[e,]**								X		X	X	X	X	X
**VIRAL TESTING**														
**EBV[f]**	X		X			X								
**Adenovirus PCR [g]**	X	X	X	X	X	X								
**HHV6[g]**	X	X	X	X	X	X								
**CMV[f]**	X	X	X	X	X	X	X							
**TRANSFUSION MEDICINE LABS**														
**Isohemagglutinin titer[c]**	X[m]					X[i]	X	X						
**PROCEDURES/STUDIES**														
**PFTs/6MWT[h]**							X	X	X	X	X	X	X	X
**Physical Exam**	(X)	(X)	(X)	(X)	(X)	(X)	(X)	(X)	(X)	(X)	(X)	(X)	(X)	(X)
**Brain MRI/MRA**								X		X				
**ECHO**						X[m]	X	X	X	X	X	X	X	X
**Influenza Vaccine[k]**						X	X	X	X	X	X	X	X	X
**DEXA Scan[l]**								X[l]		X[l]	X[l]	X[l]	X[l]	X[l]

RECIPIENT SCHEDULE OF ASSESSMENTS - TABLE A (continued)

* Annual visits optional beyond 5 years.

Coagulation Screen: PT/PTT
FASTING Lipid profile, Lipoprotein profile & Apolipoprotein B at 2 weeks, 1 month, 2 months & 100 days (+/-7days). Serum Cholesterol & triglycerides monthly starting monthly (+/-7days) in follow up to D+100.
Isohemagglutinin titer not indicated if recipient and donor have the same ABO type.
To be done twice weekly while patient is an inpatient: Reticulocyte count, Pre-albumin, sirolimus level and coagulation screen. Sirolimus levels (for engrafted patients) at least every 2-3 weeks or as clinically indicated up to day 100.
Endocrine Testing: Thyroid panel (TSH, T3, T4), Insulin -like growth factor 1, Morning Cortisol, fasting glucose, hgb-A1c, fasting insulin, serum fructosamine level, Hgb A1Cand 25 hydroxy Vitamin D. Growth Hormone, Adrenocorticotropic hormone, Progesterone. Also FSH, LH (males & females), Testosterone (males only; Total & Free), Anti-mullerian hormone (AMH) & Estradiol (females only). Prolactin (females).
CMV/EBV: CMV PCR, EBV PCR. CMV PCR to be baseline & weekly to D+100. CMV monitoring will continue for at least 6 months post-transplant. Thereafter, as clinically indicated. EBV to be done baseline & at least weekly until discharge from hospital and then at least q 2weeks until D+100.
HHV6/Adenovirus PCR to be done at baseline & at least weekly until discharge from hospital. Then at least q2-3 weeks until Day +100.
PFTs will also include 6 min walk test.
i. +/- 7 days
j. +/- 3 months
Influenza vaccine: will be offered to recipients when at least 6 months post-transplant as seasonally indicated.
Dexa scan in all patients pre – transplant and every 1-2 years post – transplant depending on how severe their bone disease is.
Pro-BNP to be done with each timepoint ECHO

RECIPIENT SCHEDULE OF RESEARCH STUDIES

	Baseline** (Pre-Transplant)	D-21	D-20	D-14	D-13	DAY 0	D+7	D+14	D+21	D+28	D+30	D+42 (6weeks)	D+60/2mos	3 months	D+100	6 months	12 month/1year	18 month/1.5yr	24 month/2yr	36 month/3yr	48 month/4yr	60 month/5yr	Beyond 5 yr*
Cytokine/Lymphocyte Function[h]	X															X[q]	X[q]						
Markers of Graft Rejection, Cytokines	X						X[o]	X[o]	X[o]		X[o]	X[o]	X[o]		X[o]	X[q]	X[q]	X[q]	X[q]	X[q]	X[q]	X[q]	X*
ESR, CRP (High sensitivity)	X						X[o]	X[o]	X[o]		X[o]	X[o]	X[o]		X[o]	X[q]	X[q]	X[q]	X[q]	X[q]	X[q]	X[q]	X*
Bone Marrow Samples[a]	X														X[a]		X[aq]	X[aq]					
Stem Cells from Back up (Autologous) Collection	X																						
Chimerism Studies[b]				X[b]							X[o]		X[o]		X[bo]	X[bq]	X[bq]	X[bq]	X[bq]	X[bq]	X[bq]	X[bq]	X*
Pharmacogenomic Sample[e]	X																						
Pentostatin PKs[d]		X	X																				
Campath PKs[k]							X[o]	X[o]	X[o]	X[o]													
Cardiac MRI & Cardiac Fibrosis Markers	X																		X[q]				
Right Heart Catheterization[f]	(X)														X[f]								
PROMIS 57 (QOL)[l]	X										X[j]		X[j]		X[j]	X[j]	X[p]		X[p]	X[q]	X[q]	X[q]	X*
Neuropsych Testing[g]	X														X[gq]		X[gp]		X[gp]				
Fertility Questionnaires	X																X		X	X	X	X	X*
HLA Antibodies[n]	X[n]						X[o]	X[o]	X[o]		X[o]		X[o]		X[o]	X[q]	X[q]						
C1q Testing[r]	X						X[o]	X[o]	X[o]		X[o]		X[o]		X[o]	X[q]	X[q]						
Functional Fluidics	X													X		X	X						
Eaton studies	X													X		X	X						
Serum Quantitative Immunoglobins	X										X[o]		X[o]		X[o]	X[q]	X[q]	X[q]	X[q]	X[q]	X[q]	X[q]	X*
Lymphocyte Subpopulation (TBNK)	X										X[o]		X[o]		X[o]	X[q]	X[q]	X[q]	X[q]	X[q]	X[q]	X[q]	X*
Plerixafor Mobilized Cells (autologous back up collection)[m]	X[m]																						

(X) as clinically indicated

* Annual visits optional beyond 5 years.
** After the protocol consent for 17-H-0069 has been signed.
Bone Marrow aspirate (up to 25ml) collected at baseline, D+100 and/or when full donor erythroid chimerism is attained, 12 to 18 months post-transplant or as clinically indicated. Used to evaluate graft rejection/tolerance and evaluate for bone marrow morphology & cytogenetics. Bone marrow core sample to be done at baseline and then only as clinically indicated.
Chimerism Studies: First sample will be drawn on Day +14 (or when subject starts to engraft), then D+30, 60, 100 and every 6-12months thereafter.
Cyclophosphamide PKs (3ml each time) : to be drawn just prior to the start of the infusion and then 0.5 hours, 1 hour, 4 hours, 6 hours, 12 hours, 24 hours **after the start** of the cyclophosphamide infusion. All timepoints have (+/-5 minutes).
Pentostatin PKs (5ml each time): to be drawn just prior to the start of the infusion, 5 minutes, 10 minutes, 2 hours, 6 hours, 8 hours, and 24 hours after the **end** of the pentostatin infusion. All timepoints have (+/-5 minutes).
Pharmacogenomic sample: Approx 10ml. drawn just prior to transplant. If the original sample has insufficient DNA for analysis, then an additional sample will be obtained from SCD patients prior to transplant.
Right heart catheterization: for those diagnosed with pulmonary hypertension prior to transplant **may** undergo right heart catheterization at least 1 year post transplant.
Neuropsychologic testing to be done at baseline, 1 year, 2 years (+/-6months) post BMT. In addition a brief monitoring battery is administered at D+100 (+/-3months) post BMT.
Cytokine/Lymphocyte function: collected pre transplant & at 6 and 12 months post-transplant – these samples are included in the 44ml of blood drawn for the graft rejection markers. NOTE: Engrafted patients who discontinue sirolimus/other immunosuppression agent will have **additional samples collected** just prior to and 6 months after stopping sirolimus/other immunosuppression agent. In patients that develop graft rejection or GVHD- recipient lymphocytes/serum will be collected once within 2 weeks of the onset of clinical symptoms, once just prior to discontinuing immunosuppression or at 12 months post-transplant (whichever is longer) and once 6 months after immunosuppression is discontinued or at 18 months post-transplant whichever is longer. Levels may continue to be followed every 6 to 12 months thereafter based on the results during the first 18 months.
Day 100 timepoint has a window of +/-7days.
j. +/- 1month
Campath PKs: samples are drawn D+7, D+14, D+21 and D+28 post-transplant (+/-7days).
PROMIS 57: will be administered either electronically or with pencil and paper pre-BMT, day 100, one year, and 2 years post BMT (+/-1month). If possible, the questionnaire will also be completed on day 30, day 60, 6 months, three years, four years, and five years post BMT (+/- 1 month). Then annually after 5 years post-HSCT if possible. Fertility questionnaires may also be completed annually after 5 years post HSCT.
Research blood samples may be drawn before plerixafor administration and 2 hours after plerixafor to study cytokine levels and CD3, CD34, and total nucleated cell counts.
Separate sample to the HLA antibody screen for clinical testing. In patients with DSAs, 5mL blood will also be collected weekly post-transplant (+/- 7 days) until day 30 for HLA antibodies.
For all recipients HLA antibodies will be collected on days 30, 60, 100 (+/-7 days) and then at 6 months and 1 year post-transplant (+/- 3 months).
o. +/- 7days
p. +/-6 months
q. +/-3 months
r. Blood samples (10mL) will be collected from recipients with DSAs to check C1q levels pre-transplant and post-transplant weekly until day 30, then at day 60 and day 100 (+/- 7 days), and at 6 months and 1 year (+/- 2 months)
s. Four peripheral blood samples, 3ml each will be drawn from the recipient at the following timepoints: (1.) Baseline (2 samples at baseline; one sample done 2 consecutive weeks after hydroxyurea is stopped prior to red blood cell exchange and plerixafor autologous collection (2.) One sample 6 months post-transplant (3.) One sample 12 months post-transplant. This will be done for approximately 11 patients starting October 2020.

RECIPIENT SCHEDULE OF EVENTS PRE TRANSPLANT TO D+21

(x) = as clinically indicated<br><br>Procedure	Screening	Baseline	>48 hrs to D-21	At least 1day 6efore e D- 21	D-21	D-20	D-19	D-18	D-17	D-16	D-15	D-14	D-13	D-12	D-11	D-10	D-9	D-8	D-7	D-6	D-5	D-4	D-3	D-2	D-1	DAY 0	D+1	D+2	D+3	D+4	D+5	D+7	D+14	D+21
Consent Forms[a]	X	X																																
Med hx & Physical[b]	X																																	
Vital Signs, Ht/Wt	X																																	
ECOG	X																																	
LAB TESTS																																		
CBC w/diff[c]	X				X			X				X				X		X	X	X	X	X	X	X	X	X	X	X	X	X	X	X	X	X
Hgb electrophoresis[d]	X																																	
Iron & Transferrin		X																																
β-HCG (Serum)[e]		X																																
Coagulation Screen[f, g]	X																	X																
d-dimer	X																																	
Reticulocyte count[g]	X																	X																
Pre-albumin[g]		X																X																
CMP[h] (Acute care panel, hepatic panel, mineral panel)		X			X			X				X				X		X	X	X	X	X	X	X	X	X	X	X	X	X	X	X	X	X
Total protein, Creatinine kinase, LDH, Uric acid[h]		X														X		X	X	X	X	X	X	X	X	X	X	X	X	X	X	X	X	X
Serum Creatinine[i]					X			X				X				X																		
Cystatin-C [g, i]		X			X			X				X				X																		
Complete Lipid Profile[j] & Triglycerides[k]		X																X k													X k		X K	X[k]
Lipoprotein Profile & Apolipoprotein B		X																															X	
Osmolality Serum		X																																
Troponin T or I		X																																
24 hr Urine[l]		X																																
Random Urine[m]		X																																
Folate, Vitamin B12, Pro Brain Naturetic Peptide		X																																
Isohemagglutinin Titer		X																																
TRANSFUSION MEDICINE LABS																																		
Extended Red Cell Phenotyping		X																																
Haptoglobin		X																																
Type & Antibody Screen	X																																	
Direct Antiglobulin Screen		X																																

(x) = as indicated	Screening	Baseline	>48 hrs to D-21	At least1 day Before D-21	D-21	D-20	D-19	D-18	D-17	D-16	D-15	D-14	D-13	D-12	D-11	D-10	D-9	D-8	D-7	D-6	D-5	D-4	D-3	D-2	D-1	DAY 0	D+1	D+2	D+3	D+4	D+5	D+7	D+14	D+21
**HLA TYPING**																																		
HLA Typing[n]	X	X																																
HLA Abx Screen[n]	X	X																																
KIR Genotyping		X																																
STR Profile		X																																
**ANTIBODY SCREEN**																																		
HBV[o]	X																																	
HCV[p]	X																																	
HIV ½ Antibody	X																																	
HHV6[q]		X																	X							X						X	X	X
Adenovirus PCR [q]		X																	X							X						X	X	X
Anti-Adenovirus Antibody		X																																
HTLV-I/II		X																																
CMV[r]	X				X							X							X							X						X	X	X
EBV[q]	X				X							X							X							X						X	X	X
Toxoplasma[w]		X																																
Syphilis, Babesia		X																																
HIV-1/HCV/HBV NAT		X																																
Anti Varicella IgG		X																																
West Nile		X																																
T.Cruzi		X																																
Anti-HSV Type ½		X																																
Anti-HAV[o]		X																																
PPD or quantiferon testing (TB)		(X)																																
Malaria		(X)																																
**PROCEDURES/ STUDIES**																																		
Exchange transfusion[u]			XX	XX																														
Autologous Backup collection[v]		X[v]																																
Chest X ray	X																																	
PFTs[s]	X																																	
CT scans[x]		(X)																																
Brain MRI/MRA	X																																	
ECHO[y]	X																																	
EKG		X																																
24 hr Holter		X																																
Abdominal US		X																																
Abdominal MRI[z]		X																																
CVAD Line																		X																
Liver biopsy[aa]		(X)																																
DEXA Scan		X																																

(x) = as indicated	Screening	Baseline	>48 hrs to D-21	At least 1day befor e D-21	D-21	D-20	D-19	D-18	D-17	D-16	D-15	D-14	D-13	D-12	D-11	D-10	D-9	D-8	D-7	D-6	D-5	D-4	D-3	D-2	D-1	DAY 0	D+1	D+2	D+3	D+4	D+5	D+7	D+14	D+21
**ENDOCRINE TESTING**																																		
**Endocrine Labs[t]**		X																																
**ACTH Stim Test**		X																																
**OGTT**		X																																
**CONSULTS**																																		
**Endocrine Consult**		(X)																																
**Nutrition Consult**		X																																
**Dental Consult**		X																																
**Radiation Oncology Consult + Sim**		X																																
**Social Work**		X																																
**Ophthalmology Consult**		X																																
**Infections Disease Consult**		X																																
**DTM Consult[bb]**		X																																
**Pulmonary Consult**		(X)																																
**Hepatology Consult**		(X)																																
**Gynecology Consult (females)**		X																																
**Sperm/testicular tissue or oocyte banking if requested**		(X)																																
**Pre transplant class & unit tour**		X																																
**TREATMENTS/ WASHOUTS**																																		
**Initiate HU[cc]**		X																																
**Stop Iron Chelation Tx[dd]**			X																															
**Stop HU Tx[dd]**				X																														
**Pentostatin**					X				X				X				X																	
**Olanzapine**					X	X	X		X	X	X		X	X	X		X	X	X															
**Oral Cyclophosphamide [ee]**					X	X	X	X	X	X	X	X	X	X	X	X	X	X																
**Campath[ee]**																			X	X	X	X	X											
**TBI[ff]**																									X									
**Stem Cell Infusion**																										X								
**IV Cyclophosphamide**																													X					
**Sirolimus**																														X	X	X	X	X
**Penicillin VK[gg]**																										X								
**Sirolimus Levels [hh]**																														X	X	X	X	X

HU=hydroxyurea; CVAD = Central venous access device (central venous line); Abx = antibody; LDH = lactate dehydrogenase; CMP = Complete metabolic profile

Recipient to sign 97-H-0041/20-H-0099 for pre-transplant evaluation, 17-H-0069 (Treatment Consent)
Determine baseline symptoms
CBC with Differential: to be done daily once patient is an inpatient.
Hemoglobin electrophoresis **and/or** flow cytometric analysis of hemoglobin A, F and SS, SC, S-β $thal^0$, or other sickle genotypes as appropriate (for both patient and donor).
β-HCG serum test for pregnancy-child bearing potential females to be done within 1 week of signing the protocol consent.
Coagulation Screen: PT/PTT
To be done twice weekly while patient is an inpatient: Reticulocyte count, Pre-albumin, Cystatin-C and coagulation screen.
To be done daily while inpatient.
Pentostatin: can lead to renal impairment, therefore serum creatinine & Cystatin C levels need to be obtained prior to each Pentostatin dose (i.e. ideally the day before each dosing D-22, D-18, D-14, D-10). See pg19 of protocol. Ensure CBC and CMP labs also done at these time points.
Complete Lipid Profile, Lipoprotein profile and Apolipoprotein B – these labs all require patient is FASTING. At 2 weeks window of (+/-7days).
Serum cholesterol and triglycerides (Lipid Profile) to be done every 2 weeks as part of the inpatient monitoring.
24 hr urine collection (ensure serum Creatinine done within 24 hours of the collection): Creatinine clearance, Creatinine, Protein, Albumin, Phosphorus (Inorganic), Uric Acid & UPEP
Random Urine: Urinalysis, protein/Creatinine ratio, Albumin/Creatinine ratio, Phosphorous (inorganic), Uric Acid, Osmolality urine
HLA typing: High resolution HLA DR, DQ. Sequence Based HLA- A, -B, -Cw. HLA A, B, C and DRB1, and DQB1 typing of patient and as many family members as possible and/or necessary to confirm haploidentical matching of the donor. HLA Antibody Screen: Class 1 A, B, C Class 2 DRB1, DQB1. Confirmatory HLA: A, B, C DRB1.
HBV Testing: Anti-Hbc IgM, Anti-HBs antibody, HBsAg, Anti-HBc antibody. HBV DNA if indicated. HAV: HAV Antibody total, HAV IgM.
HCV: Anti-HCV Antibody
HHV6/Adenovirus/EBV to be done at baseline & weekly until discharge from hospital. Then at least q2weeks until Day +100. Thereafter as clinically indicated.
CMV PCR to be baseline & weekly (i.e. starting at D-21) until inpatient D-8. Once inpatient to be done twice weekly and then as outpatient up to D+100 at least once weekly. CMV monitoring will continue for at least 6 months post-transplant Thereafter, as clinically indicated.
PFT evaluation includes 6 min walk test
Endocrine Testing: Thyroid panel (TSH, T3, T4), Insulin -like growth factor 1, Morning Cortisol, fasting glucose, fasting insulin, serum fructosamine level. Hgb A1c and 25 hydroxy Vitamin D. Growth Hormone, Adrenocorticotropic hormone, Progesterone. Also FSH, LH (males & females), Testosterone (males only; Total & Free), Anti-mullerian hormone (AMH) & Estradiol (females only). Prolactin (females).
Exchange transfusion: must be done just prior to starting preparative conditioning regimen for a target HgbS<30% prior to D-21.
Backup Collection of autologous stem cells: must be done pre transplant – this is will be done via Plerixafor mobilization or via bone marrow harvest in OR. Note: BM harvest requires overnight admission s/p procedure. At the post-collection follow up visit the provider will complete the post-plerixafor PBSC collection adverse event form.
Toxoplasma: Anti-Toxoplasma IgG & IgM
CT scans: include sinus, chest, abdomen and pelvis (only as clinically indicated).
Echocardiogram: Done at baseline to screen for pulmonary hypertension (HTN). If pulmonary HTN – then CONSULT with Pulmonology required.
Abdominal MRI w/Dr. Gharib's group (T2*) after consent is signed.
Liver Biopsy (if indicated per hepatology).
DTM consult and also venous assessment. Ensure coordination for assessment for exchange transfusions & back up auto collection.

cc. Hydroxyurea will be initiated or continued prior to the conditioning regimen if applicable.
dd. WASHOUTS: (1) Iron chelation tx must be stopped >48 hours prior to starting the conditioning regimen.(2) HU (hydroxyurea) must be stopped at least 1 day prior to conditioning regimen.
ee. Campath: target is ALC<100 & ANC without grade 3 toxicity. See pg 19 of protocol. Cyclophosphamide dose may be omitted or reduced depending on whether the depletion target has been achieved or whether there is any myeloid toxicity.
ff. TBI: Gonadal shielding will be used in males unless refused.
gg. Penicillin VK from Day 0 until pneumococcal vaccination is complete post-transplant.
hh. Sirolimus levels twice a week while patient is inpatient and then for engrafted patients at least every 2-3 weeks or as clinically indicated up to day 100.

APPENDIX H: POWER ANALYSIS OF ABILITY TO DETECT A DIFFERENCE IN PRIMARY OUTCOME BY RADIATION REGIMEN

This analysis examines the likelihood of seeing a significant difference in the primary outcome between the cohort of 19 with a two-does radiation regimen and the cohort of 20 with a single dose regimen.

In the 18 individuals thus evaluated for the 100 day primary endpoint (one of the 19 has not yet reached 100 days although the results are similar for whichever outcome this person has) there have been 2 failures thus far - an empirical failure probability of 11%. What failure probabilities for the 20 individuals with the one dose regimen are consistent with moderate or high statistical power for detecting a difference between the two cohorts?

The table below shows the power of detecting a significant difference in the 100 day failure probabilities for the two cohorts as a function of the true underlying probability of failure in the new cohort of 20 people (the cohort of 18 is already known to have an empirical failure probability of 11%). Power was calculated using a two-sided version of Fisher's exact test with a 5% level of significance.

Prob.	Power
0.10	0%
0.20	1%
0.30	11%
0.40	40%

The table indicates that if the true probability of 100 day failure for the proposed radiation regimen is 0.20, then there is only a 1% chance we will find a statistically significant difference in failure probability between the existing and new cohort. If the true failure rate is 0.30 then statistical power increases to 11%. We do not really expect to have failure probabilities as high as 30% or higher so we think it unlikely the two regimens will show evidence of different primary outcome probabilities.

APPENDIX I : Reproductive/Fertility Questionnaires

1. CSFQ-F-C U01 COALESCE (Females)
2. CSFQ-F-C U01 COALESCE (Males)
3. Fertility Survey U01 COALESCE (Females)
4. International Index Of Erectile Function U01 COALESCE (Males)
5. Priapism Impact Survey U01 COALESCE (Males)

Note: *Due to formatting irregularities, the above surveys are also attached in the PROTECT study application as stand-alone documents for reference.*

1. Changes in Sexual Functioning Questionnaire (CSFQ-F-C) for Females

Please complete the survey below.

Thank you!

This is a questionnaire about sexual activity and sexual function. By sexual activity, we mean sexual intercourse, masturbation, sexual fantasies and other activity.

Today's Date

____

Compared with the most enjoyable it has ever been, how pleasure enjoyable or pleasurable is your sexual life right pleasure
now?

○ 1-No enjoyment or
○ 2-Little enjoyment or
○ 3-Some enjoyment or pleasure
○ Much enjoyment or pleasure
○ Great enjoyment or pleasure

How frequently do you engage in sexual activity (sexual intercourse, masturbation, etc.) now?

○ 1-Never
○ 2-Rarely (once a month or less)
○ Sometimes (more than once a month, up to twice a week)
○ Often (more than twice a week)
○ Every day

How often do you desire to engage in sexual activity?

○ 1-Never
○ Rarely (once a month or less)
○ Sometimes (more than once a month, up to twice a week)
○ Often (more than twice a week)
○ Every day

How frequently do you engage in sexual thoughts (thinking about sex, sexual fantasies) now?

○ 1-Never
○ 2-Rarely (once a month or less)
○ Sometimes (more than once a month, up to twice a week)
○ Often (more than twice a week)
○ Every day

Do you enjoy books, movies, music or artwork with sexual content?

○ 1-Never
○ 2-Rarely (once a month or less)
○ Sometimes (more than once a month, up to twice a week)
○ Often (more than twice a week)
○ Every day

How much pleasure or enjoyment do you get from pleasure thinking about and fantasizing about sex? pleasure

○ 1-No enjoyment or
○ 2-Little enjoyment or
○ Some enjoyment or pleasure
○ Much enjoyment or pleasure
○ Great enjoyment or pleasure

How often do you become sexually aroused?

○ 1-Never
○ Rarely (once a month or less)
○ Sometimes (more than once a month, up to twice a week)
○ Often (more than twice a week)
○ Every day

Are you easily aroused?

○ **1-Never**
○
○
○

Rarely (much Jess than half the time) 3-Sometimes (about half the time)
4-Often (much more than half the time) 5-Always

Do you have adequate vaginal lubrication during sexual activity?
Sometimes (about half the time)
Often (much more than half the time) 5-Always

1-Never
2-Rarely (much less than half the time)

How often do you become aroused and then lose interest?
3-Sometimes (about half the time)
2-Often (much more than half the time) 1-Always

5-Never
4-Rarely (much less than haffthe time)

How often do you experience an orgasm?
2-Rarely (much less than half the time) 3-Sometimes (about half the time)
4-Often (much more than half the time) 5-Always

1-Never

Are you able to have an orgasm when you want to?
Rarely (much less than half the time) 3-Sometimes (about half the time)
4-Often (much more than half the time) 5-Always

1-Never

How much pleasure or enjoyment do you get from your pleasure orgasms?
pleasure
Some enjoyment or pleasure
Much enjoyment or pleasure
Great enjoyment or pleasure

1-No enjoyment or
2-Little enjoyment or

How often do you have painful orgasm?
4-Rarely (once a month or less)
3-Sometimes (more than once a month, up to twice a week)
2-Often (more than twice a week) 1-Every day

5-Never

2. Changes in Sexual Functioning Questionnaire (CSFQ-M-C) for Males

Please complete the survey below.

Thank you!

This is a questionnaire about sexual activity and sexual function. By sexual activity, we mean sexual intercourse, masturbation, sexual fantasies and other activity.

Today's Date

____

Compared with the most enjoyable it has ever been, how pleasure enjoyable or pleasurable is your sexual life right pleasure now?

1-No enjoyment or
2-Little enjoyment or
3-Some enjoyment or pleasure
Much enjoyment or pleasure
Great enjoyment or pleasure

How frequently do you engage in sexual activity (sexual intercourse, masturbation, etc.) now?
Sometimes (more than once a month, up to twice a week)
Often (more than twice a week)
Every day

1-Never
2-Rarely (once a month or less)

How often do you desire to engage in sexual activity?
Rarely (once a month or less)
Sometimes (more than once a month, up to twice a week)
Often (more than twice a week)
Every day

1-Never

How frequently do you engage in sexual thoughts (thinking about sex, sexual fantasies) now?
Sometimes (more than once a month, up to twice a week)
Often (more than twice a week)
Every day

1-Never
2-Rarely (once a month or less)

Do you enjoy books, movies, music or artwork with sexual content?
Sometimes (more than once a month, up to twice a week)
Often (more than twice a week)
Every day

1-Never
2-Rarely (once a month or less)

How much pleasure or enjoyment do you get from pleasure thinking about and fantasizing about sex?
pleasure
Some enjoyment or pleasure
Much enjoyment or pleasure
Great enjoyment or pleasure

1-No enjoyment or
2-Little enjoyment or

How often do you have an erection related or unrelated to sexual activity?
Sometimes (more than once a month, up to twice a week)
Often (more than twice a week)
Every day

1-Never
2-Rarely (once a month or less)

Do you get an erection easily?
2-Rarely (much Jess than half the time) 3-Sometimes (about half the time)

1-Never

4-Often (much more than half the time) 5-Always

Are you able to maintain an erection? ◯ 1-Never
2-Rarely (much less than half the time) 3-Sometimes (about half the time)
4-Often (much more than half the time) 5-Always

How often do you experience painful, prolonged erections? ◯ 5-Never
4-Rarely (once a month or less)
3-Sometimes (more than once a month, up to twice a week)
2-Often (more than twice a week) 1-Every day

How often do you have an ejaculation? ◯ 1-Never
Rarely (once a month or· less)
Sometimes (more than once a month, up to twice a week)
Often (more than twice a week)
Every day

Are you able to ejaculate when you want to? ◯ 1-Never
Rarely (much less than half the time) 3-Sometimes (about half the time)
4-Often (much more than half the time) 5-Always

How much pleasure or enjoyment do you get from your pleasure orgasms? ◯ 1-No enjoyment or
2-Little enjoyment or
pleasure
Some enjoyment or pleasure
Much enjoyment or pleasure
Great enjoyment or pleasure

How often do you have painful orgasm? ◯ 5-Never
4-Rarely (once a month or less)
3-Sometimes (more than once a month, up to twice a week)
2-Often (more than twice a week) 1-Every day

3. Fertility Survey (Females)

Please complete the survey below.

Thank you!

Introductory Questions

In this survey, we hope to learn more about your reproductive and sexual history and experiences before and after bone marrow transplant.

How old were you when you first got your period?

(______________________________ (years))

Before transplant, did you get your period?

○ Ye

s No ○

Do you currently take hormone therapy for birth control, period regulation, or hormone replacement/ovarian failure?

○ Yes
○ No

Why do you take hormones?

Birth control Period regulation
Ovarian failure/hormone replacement
(Check all that apply.)

Which female hormone do you take for birth control?

Estrogen pills (Ex: Premarin)
Estrogen patches
Birth control pills
Progesterone pills (Provera or Prometrium)
Progesterone-releasing IUD
Other
(Check all that apply.)

Please specify which female hormone you take for birth control. ______________

Which female hormone do you take for period regulation?

○ Pills containing only estrogen
○ Patches containing only estrogen

Cream containing only estrogen ○
Pills containing estrogen and progesterone Patches containing ○ estrogen & progesterone Vaginal ring containing estrogen & progesterone Pills containing only progesterone ○
Depo Provera ("the shot") ○
Intrauterine Device (IUD) containing progesterone Implant containing progesterone ○
Other ○
○
○
○

Please specify which female hormone you take for period regulation. ______________

Which female hormone do you take for ovarian failure/hormone replacement?
estrogen Cream containing only estrogen
Pills containing estrogen and progesterone Patches containing estrogen & progesterone Vaginal ring containing estrogen & progesterone Pills containing only progesterone
Depo Provera ("the shot")
Intrauterine Device (IUD) containing progesterone Implant containing progesterone
Other

- ◯ Pills containing only estrogen
- ◯ Patches containing only
- ◯
- ◯
- ◯
- ◯
- ◯
- ◯
- ◯
- ◯
- ◯

Please specify which female hormone you take for ovarian failure/hormone replacement.

Have you ever been diagnosed with a sexually transmitted infection?

- ◯ Yes
- ◯ No

Were you diagnosed with a sexually transmitted infection before transplant, after transplant, or before and after transplant?

- ◯ Before
- ◯ After
- ◯ Before and after

Have you ever been diagnosed with pelvic inflammatory

s disease?

- ◯
- ◯ Ye
- No

Have you been diagnosed with pelvic inflammatory disease before transplant, after transplant, or before and after transplant?

- ◯ Before
- ◯ After
- ◯ Before and after

Have you ever been diagnosed with polycystic ovary syndrome (PCOS)?

- ◯ Yes
- ◯ No

Were you diagnosed with PCOS for the first time before

e or after transplant?

- ◯
- ◯ Befor
- After

Have you ever been diagnosed with diabetes?

s No

- ◯ Ye
- ◯

Were you diagnosed with diabetes before or after transplant?

- ◯ Before
- ◯ After

Have you ever been diagnosed with endometriosis or fibroids?

- ◯ Yes
- ◯ No

Were you diagnosed with endometriosis or fibroids before or after transplant?

- ◯ Before
- ◯ After

Have you ever been diagnosed with a rheumatologic disorder (rheumatoid arthritis, lupus, or autoimmune hepatitis)?

- ◯ Yes
- ◯ No

Were you diagnosed with a rheumatologic disorder before or after transplant?

- ◯ Before
- ◯ After

Before transplant, were you offered fertility preservation treatment?
I don't know

- ◯ Yes
- ◯ No
- ◯

Before transplant, did you undergo fertility preservation treatment?
I don't know

◯ Yes
◯ No
◯

After transplant, were you offered fertility preservation treatment?
I don't know

◯ Yes
◯ No
◯

After transplant, did you do fertility preservation treatment?
I don't know

◯ Yes
◯ No
◯

Before transplant, were you ever pregnant?

s No

◯
◯ Ye
◯

After transplant, did you ever become pregnant?

s No

◯
Ye

MENSES BEFORE BMT

Before transplant, how frequent were your periods?
Every 2-3 months
Every 3-6 months 2-3 times a year Other

◯ Every month
◯
◯
◯
◯

Please specify the frequency of your periods before the transplant. ______

Why were your periods not regular?
I had premature ovarian insufficiency (menopause) I don't know

◯ Birth control or hormones suppressed my period
◯
◯

During transplant, did your periods stop?

s No

◯ Ye
◯

How were your periods stopped?
contraception Lupron

◯
◯ Menstrual suppressing

What was the contraception?
Intrauterine Device (IUD)

◯
◯ Depo-Provera

MENSES AFTER BMT if PATIENT HAD MENSES BEFORE BMT

After transplant, did your period start again?

s No

◯
◯
Ye

How long after transplant did your period restart?

Years after transplant. Months after transplant.

______ ______

How often do you get your period now?
Every 2-3 months
Every 3-6 months 2-3 times a year Other

◯ Every month
◯
◯
◯
◯

Please specify how often you get your period now.

____

Do you know why your period never started after transplant?
Other

◯ Birth control pills
◯ Premature ovarian insufficiency (menopause)
◯

Please explain why your period never started after transplant. ______________________________

MENSES AFTER BMT if PATIENT DID NOT HAVE MENSES BEFORE BMT

After transplant, did you start your periods?

s No

Ye
◯
◯

How long after transplant did your period start?

Years after transplant. Months after transplant.

_____ _____

How often do you get your period now?
Every 2-3 months
Every 3-6 months 2-3 times a year Other

◯ Every month
◯
◯
◯
◯

Please specify how often you get your period.

____

Do you know why your period never started again after transplant?
Other (explain)

◯ Birth control pills
◯ Premature ovarian insufficiency (menopause)
◯

Please explain why your period never started again after transplant. ______________________________

FERTILITY PRESERVATION

Before transplant, did you take a medicine called depot leuprolide or Lupron?
I don't know

◯ Yes
◯ No
◯

Did you freeze eggs/embryos or ovarian tissue?

s No

◯
◯ Ye

What did you freeze?

☐ Eggs Embryos
☐ Ovarian tissue
☐ (Check all that apply.)

How many eggs were frozen? ◯ N =
I don't know ◯

Please note the number of eggs that were frozen. ____________________

How many embryos were frozen? ◯ N = _______
I don't know ◯

Please note the number of embryos that were frozen. ____________________

How many times did you have eggs harvested before transplant? ____________________

Were you taking hydroxyurea at the time of your egg harvest?
◯ Yes
◯ No

Did it cause a disruption in the fertility preservation process?
◯ Yes
◯ No

Did you stop taking hydroxyurea to prepare for your egg harvest?
◯ Yes
◯ No

Did you have any of the following problems during the fertility preservation process (including taking the medicines and the actual procedure to harvest eggs)?
☐ Painful crisis
☐ Acute chest syndrome
☐ Ovarian hyperstimulation syndrome
☐ Other
☐ None
(Check all that apply.)

Please specify the problem during the fertility preservation process. ____________________

BARRIERS TO FERTILITY PRESERVATION

On a scale from 1 to 5 with 1 being not a problem and 5 being a significant problem, which of the following issues either prevented or complicated fertility preservation treatment for you? (Please use a scale of 1 to 5, 1 = not a problem, 5 = a significant problem)

Freezing eggs, embryos or ovarian tissue was not offered to me.
◯ 1
◯ 2
◯ 3
◯ 4
◯ 5

I declined to freeze eggs, embryos or ovarian tissue.
◯ 1
◯ 2
◯ 3
◯ 4
◯ 5

I had health problems or was sick in a way that prevented fertility preservation.
◯ 1
◯ 2
◯ 3
◯ 4
◯ 5

My family was already complete.

1
2
3
4
5

I could not afford egg, embryo or ovarian tissue freezing.

1
2
3
4
5

I could not afford the annual rates to keep eggs, embryos or ovarian tissue frozen.

1
2
3
4
5

I didn't think it was necessary to freeze eggs, embryos or ovarian tissue.

1
2
3
4
5

I thought I was already infertile from sickle cell disease, so freezing eggs, embryos or ovarian tissue wouldn't be possible.

1
2
3
4
5

I tried to freeze eggs, embryos, or tissue, but it didn't work.

1
2
3
4
5

PREGNANCY BEFORE BMT

Before transplant, how many times have you been pregnant?

1
2
3
4
5

For each pregnancy before transplant, please answer the following questions.

How did you become pregnant?

- I got pregnant naturally and did not require medical assistance
- My frozen eggs
- My frozen embryos
- Donor eggs and my partner's sperm
- My eggs and donor sperm
- IVF with donor eggs and my partner's sperm
- Other

Please specify how you became pregnant.

____

What was the outcome of first pregnancy?
term Livebirth, preterm Stillbirth Miscarriage Ectopic pregnancy

Livebirth, full

Did you have any complications with first pregnancy?

Hypertension/Pre-eclampsia/Eclampsia
Blood clot
Infection
Bleeding Preterm
Labor C-section
Intrauterine growth restriction or small for gestational age fetus (small baby)
Gestational hypertension
Gestational diabetes Other
(Check all that apply.)

Please specify the complication with first pregnancy.

____

How did you become pregnant the second time?
medical assistance My frozen eggs
My frozen embryos
Donor eggs and my partner's sperm My eggs and donor sperm
IVF with donor eggs and my partner's sperm Other

I got pregnant naturally and did not require

Please specify how you became pregnant the second time. ______________________________

What was the outcome of second pregnancy?
term Livebirth, preterm Stillbirth Miscarriage Ectopic pregnancy

Livebirth, full

Did you have any complications with second pregnancy?

Hypertension/Pre-eclampsia/Eclampsia
Blood clot
Infection
Bleeding Preterm
Labor C-section
Intrauterine growth restriction or small for gestational age fetus (small baby)
Gestational hypertension
Gestational diabetes Other
(Check all that apply.)

Please specify complication with second pregnancy.

____

How did you become pregnant the third time? ◯ I got pregnant naturally and did not require medical assistance My frozen eggs
My frozen embryos
Donor eggs and my partner's sperm My eggs and donor sperm
IVF with donor eggs and my partner's sperm Other

Please specify how you became pregnant the third time.

What was the outcome of third pregnancy? ◯ Livebirth, full term Livebirth, preterm Stillbirth Miscarriage Ectopic pregnancy

Did you have any complications with third pregnancy?

Hypertension/Pre-eclampsia/Eclampsia
Blood clot
Infection
Bleeding Preterm
Labor C-section
Intrauterine growth restriction or small for gestational age fetus (small baby)
Gestational hypertension
Gestational diabetes Other
(Check all that apply.)

Please specify complication with third pregnancy.

How did you become pregnant the fourth time? ◯ I got pregnant naturally and did not require medical assistance My frozen eggs
My frozen embryos
Donor eggs and my partner's sperm My eggs and donor sperm
IVF with donor eggs and my partner's sperm Other

Please specify how you became pregnant the fourth time.

What was the outcome of fourth pregnancy? ◯ Livebirth, full term Livebirth, preterm Stillbirth Miscarriage Ectopic pregnancy

Did you have any complications with fourth pregnancy?

Hypertension/Pre-eclampsia/Eclampsia
Blood clot
Infection
Bleeding Preterm
Labor C-section
Intrauterine growth restriction or small for gestational age fetus (small baby)
Gestational hypertension
Gestational diabetes Other
(Check all that apply.)

Please specify complication with fourth pregnancy.

____

How did you become pregnant the fifth time?

I got pregnant naturally and did not require medical assistance
My frozen eggs
My frozen embryos
Donor eggs and my partner's sperm My eggs and donor sperm
IVF with donor eggs and my partner's sperm Other

Please specify how you became pregnant the fifth time.

____

What was the outcome of fifth pregnancy?

Livebirth, full term Livebirth, preterm Stillbirth Miscarriage Ectopic pregnancy

Did you have any complications with fifth pregnancy?

Hypertension/Pre-eclampsia/Eclampsia
Blood clot
Infection
Bleeding Preterm
Labor C-section
Intrauterine growth restriction or small for gestational age fetus (small baby)
Gestational hypertension
Gestational diabetes Other
(Check all that apply.)

Please specify complication with fifth pregnancy.

____

PREGNANCY AFTER BMT

After transplant, how many times have you been pregnant?

1
2
3
4
5

For each pregnancy after transplant, please answer the following questions.

How did you become pregnant after transplant? ◯ I got pregnant naturally and did not require medical assistance My frozen eggs

My frozen embryos ◯

My eggs and donor sperm ◯

IVF with donor eggs and my partner's sperm Intrauterine insemination with donor sperm Intrauterine insemination with my partner's sperm Other ◯ ◯ ◯ ◯ ◯

Please specify how you became pregnant after transplant. ____________________

What was the outcome of first pregnancy after transplant?

◯ Livebirth, fullterm

◯ Livebirth, preterm

Stillbirth Miscarriage Ectopic pregnancy ◯ ◯ ◯

Did you have any complications with first pregnancy after transplant?

☐ Hypertension/Pre-eclampsia/Eclampsia

☐ Blood clot

☐ Infection

☐ Bleeding Preterm

☐ Labor C-section

☐ Intrauterine growth restriction or small for gestational age fetus (small baby)

☐ Gestational hypertension

☐ Gestational diabetes Other

(Check all that apply.)

Please specify the complication with first pregnancy after transplant. ____________________

How did you become pregnant the second time after require transplant? ◯ I got pregnant naturally and did not medical assistance

My frozen eggs ◯

My frozen embryos ◯

My eggs and donor sperm ◯

IVF with donor eggs and my partner's sperm Intrauterine insemination with donor sperm Intrauterine insemination with my partner's sperm Other ◯ ◯ ◯ ◯

Please specify how you became pregnant the second time after transplant. ____________________

What was the outcome of second pregnancy after transplant?

◯ Livebirth, fullterm

◯ Livebirth, preterm

Stillbirth Miscarriage Ectopic pregnancy ◯ ◯ ◯

Did you have any complications with second pregnancy after transplant?

Hypertension/Pre-eclampsia/Eclampsia
Blood clot
Infection
Bleeding Preterm
Labor C-section
Intrauterine growth restriction or small for gestational age fetus (small baby)
Gestational hypertension
Gestational diabetes Other
(Check all that apply.)

Please specify the complication with second pregnancy after transplant.

How did you become pregnant the third time after require transplant?

- I got pregnant naturally and did not medical assistance
- My frozen eggs
- My frozen embryos
- My eggs and donor sperm
- IVF with donor eggs and my partner's sperm
- Intrauterine insemination with donor sperm
- Intrauterine insemination with my partner's sperm
- Other

Please specify how you became pregnant the third time after transplant.

What was the outcome of third pregnancy after transplant?

- Livebirth, fullterm
- Livebirth, preterm
- Stillbirth
- Miscarriage
- Ectopic pregnancy

Did you have any complications with third pregnancy after transplant?

Hypertension/Pre-eclampsia/Eclampsia
Blood clot
Infection
Bleeding Preterm
Labor C-section
Intrauterine growth restriction or small for gestational age fetus (small baby)
Gestational hypertension
Gestational diabetes Other
(Check all that apply.)

Please specify the complication with third pregnancy after transplant.

How did you become pregnant the fourth time after require tranplant?

- I got pregnant naturally and did not medical assistance
- My frozen eggs
- My frozen embryos
- My eggs and donor sperm
- IVF with donor eggs and my partner's sperm
- Intrauterine insemination with donor sperm
- Intrauterine insemination with my partner's sperm
- Other

Please specify how you became pregnant the fourth time after transplant? ____________

What was the outcome of fourth pregnancy after transplant?
Stillbirth Miscarriage Ectopic pregnancy

◯ Livebirth, fullterm
◯ Livebirth, preterm
◯
◯
◯

Did you have any complications with fourth pregnancy after transplant?

Hypertension/Pre-eclampsia/Eclampsia
Blood clot
Infection
Bleeding Preterm
Labor C-section
Intrauterine growth restriction or small for gestational age fetus (small baby)
Gestational hypertension
Gestational diabetes Other
(Check all that apply.)

Please specify the complication with fourth pregnancy after transplant. ____________

How did you become pregnant the fifth time after require transplant?
My frozen eggs
My frozen embryos
My eggs and donor sperm
IVF with donor eggs and my partner's sperm Intrauterine insemination with donor sperm Intrauterine insemination with my partner's sperm Other

◯ I got pregnant naturally and did not medical assistance
◯
◯
◯
◯
◯
◯
◯

Please specify how you became pregnant the fifth time after transplant? ____________

What was the outcome of fifth pregnancy after transplant?
Stillbirth Miscarriage Ectopic pregnancy

◯ Livebirth, fullterm
◯ Livebirth, preterm
◯
◯
◯

Did you have any complications with fifth pregnancy after transplant?

Hypertension/Pre-eclampsia/Eclampsia
Blood clot
Infection
Bleeding Preterm
Labor C-section
Intrauterine growth restriction or small for gestational age fetus (small baby)
Gestational hypertension
Gestational diabetes Other
(Check all that apply.)

Please specify the complication with fifth pregnancy after transplant. ____________

WHY NO PREGNANCY QUESTION BEFORE BMT

Have you tried getting pregnant?

◯
◯ Ye

s No

How long did you try getting pregnant before BMT?

Years Months

______ ______

Which of the following best describes your attempts of getting pregnant before BMT?
IVF Other

◯ Unassisted
◯ Medical Assistance with
◯

What is your other attempt of getting pregnant?

____

If you have never attempted to get pregnant, which of the following best describes the reason?

Thought I was infertile Didn't
have a partner
Felt I was too young at the time
Delayed due to school or work goals
Couldn't afford fertility treatment or evaluation Was
not interested in having a family
Reason not listed
(Check all that apply.)

What is the reason for never attempting to get pregnant? ____________________

WHY NO PREGNANCY QUESTION AFTER BMT

Have you tried getting pregnant?

◯ Ye
◯

s No

How long did you try getting pregnant after BMT?

Years Months

______ ______

Which of the following best describes your attempts of getting pregnant after BMT?
IVF Other

◯ Unassisted
◯ Medical Assistance with
◯

What is your other attempt of getting pregnant after BMT? ____________________

If you have never attempted to get pregnant after transplant, which of the following best describes the reason?

Thought I was infertile Didn't
have a partner
Felt I was too young at the time
Delayed due to school or work goals
Couldn't afford fertility treatment or evaluation Was
not interested in having a family
Reason not listed
(Check all that apply.)

What is the reason for never attempting to get pregnant after BMT? ____________

INFERTILITY

Has anyone in your family experienced infertility?

◯ Ye
◯

s No

Who has experienced infertility?

☐ Mother
☐ Grandmother
☐ Aunt
☐ Cousin

(Check all that apply.)

Has a doctor ever evaluated you for infertility?

Ye

s No

Has a doctor ever diagnosed you with infertility?

Ye

s No

Have you ever been told a cause for infertility in your case?

A problem with my tubes

A problem with my hormones

A problem with my uterus (like fibroids or endometriosis)

Bone marrow transplant

No problem was ever identified Other

What was the other cause for infertility in your case?

____

4. International Index Of Erectile Function

Please complete the survey below.

Thank you!

The purpose of this questionnaire is to capture data regarding the impact of erectile dysfunction. This form is for participants 18 years old and older. If the participant's provider is a pediatrician, we defer to the pediatrician's judgment in determining the appropriateness of this form.

1) Date ______________________________

Precede all questions listed below with the phrase, "Over the past 4 weeks,

2) How often were you able to get an erection during activity sexual activity?
Never

- 0. No sexual
- 1. Almost never/
- 2. A few times (Much less than half the time)
- 3. Sometimes (About half the time)
- 4. Most times (Much more than half the time)
- 5. Almost always/ Always

3) With sexual stimulation, how often have your erections activity been hard enough to allow for penetration (entering Never
your partner)?
time)

- 0. No sexual
- 1. Almost never/
- 2. A few times (Much less than half the
- 3. Sometimes (About half the time)
- 4. Most times (Much more than half the time)
- 5. Almost always/ Always

4) When you attempted sexual intercourse, how often were intercourse you able to penetrate (enter) your partner?

- 0. Did not attempt
- 1. Almost never/ Never
- 2. A few times(Much less than half the time)
- 3. Sometimes (About half the time)
- 4. Most times (Much more than half the time)
- 5. Almost always/ Always

5) During sexual intercourse, how often were you able to intercourse maintain an erection after penetration (entering your partner)?
time)

- 0. Did not attempt
- 1. Almost never/ Never
- 2. A few times(Much less than half the
- 3. Sometimes (About half the time)
- 4. Most times (Much more than half the time)
- 5. Almost always/ Always

6) During sexual intercourse, how difficult has it been intercourse to maintain your erections until completion of intercourse?

- 0. Did not attempt
- 1.Extremely difficult
- 2. Very difficult
- 3. Difficult
- 4. Slightly Difficult
- 5. Not difficult

7) How many times have you attempted sexual intercourse?

- 0. No attempts
- 1. One or two attempts
- 2. Three to four attempts
- 3. Five to six attempts
- 4. Seven to ten attempts
- 5. Eleven + attempts

8) When you attempted sexual intercourse, how often was intercourse it satisfactory to you?

- 0. Did not attempt
- 1. Almost never/ Never
- 2. A few rimes (Much less than half the time)
- 3. Sometimes (About half the time)
- 4. Most times (Much more than half the time)
- 5. Almost always/ Always

9) How much have you enjoyed sexual intercourse?

- 0. No intercourse
- 1. No enjoyment
- 2. Not very enjoyable
- 3. Fairly enjoyable
- 4. Highly enjoyable
- 5. Very highly enjoyable

10) When you had sexual stimulation or intercourse, how stimulation/intercourse often did you ejaculate?

- 0. No sexual
- 1. Almost never/ Never
- 2. A few times (Much less than half the time)
- 3. Sometimes (About half the time)
- 4. Most times (Much more than half the time)
- 5. Almost always/ Always

11) When you had sexual stimulation or intercourse, how stimulation/intercourse often did you have the feeling of orgasm or climax?

- 0. No sexual
- 1. Almost never/ Never
- 2. A few times (Much less than half the time)
- 3. Sometimes (About half the time)
- 4. Most times (Much more than half the time)
- 5. Almost always/ Always

12) How often have you felt sexual desire?

- 0. No sexual stimulation/intercourse
- 1. Very low/none at all
- 2. Low
- 3. Moderate
- 4. High
- 5. Very high

13) How would you rate your level of sexual desire?

- 1. Very low/ none at all
- 2. Low
- 3. Moderate
- 4. High
- 5. Very High

14) How satisfied have you been with your overall sex stimulation/intercourse life?

- 0. No sexual
- 1. Very dissatisfied
- 2. Moderately dissatisfied
- 3. About equally satisfied and dissatisfied
- 4. Moderately satisfied
- 5. Very satisfied

15) How satisfied have you been with your sexual stimulation/intercourse relationship with your partner?

- 0. No sexual
- 1. Very dissatisfied
- 2. Moderately dissatisfied
- 3. About equally satisfied and dissatisfied
- 4. Moderately satisfied
- 5. Very satisfied

16) How do you rate your confidence that you can get and low keep an erection?

- 1. Very
- 2. Low
- 3. Moderate
- 4. High
- 5. Very high

5. Priapism Impact Questionnaire

Please complete the survey below.

Thank you!

The purpose of this questionnaire is to capture data regarding the impact of priapism. This form is for participants 18 years old and older. If the participant's provider is a pediatrician, we defer to the pediatrician's judgment in determining the appropriateness of this form.

Date	______________________________
Have you ever experienced priapism?	◯ Yes ◯ No
I worry about my overall health has been:	◯ 1. None<br>◯ 2. Minimal<br>◯ 3. Slight<br>◯ 4. Moderate<br>◯ 5. Substantial<br>◯ 6. Extreme<br>◯ 7. Very Extreme
My distress about my priapism has been:	◯ 1. None<br>◯ 2. Minimal<br>◯ 3. Slight<br>◯ 4. Moderate<br>◯ 5. Substantial<br>◯ 6. Extreme<br>◯ 7. Very Extreme
The effect of priapism on my daily activities has been:	◯ 1. None<br>◯ 2. Minimal<br>◯ 3. Slight<br>◯ 4. Moderate<br>◯ 5. Substantial<br>◯ 6. Extreme<br>◯ 7. Very Extreme
The negative effect of priapism on my feelings have been:	◯ 1. None<br>◯ 2. Minimal<br>◯ 3. Slight<br>◯ 4. Moderate<br>◯ 5. Substantial<br>◯ 6. Extreme<br>◯ 7. Very Extreme
The effect of priapism on my sexual satisfaction has been:	◯ 1. None<br>◯ 2. Minimal<br>◯ 3. Slight<br>◯ 4. Moderate<br>◯ 5. Substantial<br>◯ 6. Extreme<br>◯ 7. Very Extreme

The effect of priapism on my relationship with my partner has been:

- ◯ 1. None
- ◯ 2. Minimal
- ◯ 3. Slight
- ◯ 4. Moderate
- ◯ 5. Substantial
- ◯ 6. Extreme
- ◯ 7. Very Extreme

The effect of priapism on my sexual confidence has been:

- ◯ 1. None
- ◯ 2. Minimal
- ◯ 3. Slight
- ◯ 4. Moderate
- ◯ 5. Substantial
- ◯ 6. Extreme
- ◯ 7. Very Extreme

The level of pain in my penis caused by my priapism has been:

- ◯ 1. None
- ◯ 2. Minimal
- ◯ 3. Slight
- ◯ 4. Moderate
- ◯ 5. Substantial
- ◯ 6. Extreme
- ◯ 7. Very Extreme

The abnormal shape of my penis caused by priapism has been:
Minimal

- 1. None
- ◯ 2.
- ◯
- ◯ 3. Slight
- ◯ 4. Moderate
- ◯ 5. Substantial
- ◯ 6. Extreme
- ◯ 7. Very Extreme

Physical discomfort caused by my priapism has been:

- ◯ 1. None
- ◯ 2. Minimal
- ◯ 3. Slight
- ◯ 4. Moderate
- ◯ 5. Substantial
- ◯ 6. Extreme
- ◯ 7. Very Extreme

Having trouble getting an erection has been:

- ◯ 1. None
- ◯ 2. Minimal
- ◯ 3. Slight
- ◯ 4. Moderate
- ◯ 5. Substantial
- ◯ 6. Extreme
- ◯ 7. Very Extreme

Problems with my sexual desire have been:

- ◯ 1. None
- ◯ 2. Minimal
- ◯ 3. Slight
- ◯ 4. Moderate
- ◯ 5. Substantial
- ◯ 6. Extreme
- ◯ 7. Very Extreme
